# A synthetic cyclitol-nucleoside conjugate polyphosphate is a highly potent second messenger mimic†
†Electronic supplementary information (ESI) available: Experimental protocols and NMR spectra. See DOI: 10.1039/c9sc00445a


**DOI:** 10.1039/c9sc00445a

**Published:** 2019-04-23

**Authors:** Wolfgang Dohle, Xiangdong Su, Stephen J. Mills, Ana M. Rossi, Colin W. Taylor, Barry V. L. Potter

**Affiliations:** a Medicinal Chemistry & Drug Discovery , Department of Pharmacology , University of Oxford , Mansfield Road , Oxford , OX1 3QT , UK . Email: barry.potter@pharm.ox.ac.uk ; Tel: +44-1865-271945; b Department of Pharmacology , University of Cambridge , Tennis Court Road , Cambridge , CB2 1PD , UK

## Abstract

Reactions that form *sec*–*sec* ethers are well known, but few lead to compounds with dense functionality around the *O*-linkage. Replacement of the α-glucopyranosyl unit of adenophostin A, a potent d-*myo*-inositol 1,4,5-trisphosphate (IP_3_R) agonist, with a d-*chiro*-inositol surrogate acting substantially as a pseudosugar, leads to “d-*chiro*-inositol adenophostin”. At its core, this cyclitol-nucleoside trisphosphate comprises an ether linkage between the axial 1-hydroxyl position of d-*chiro*-inositol and the 3′-hydroxyl group of an adenosine ribose sugar. A divergent synthesis of d-*chiro*-inositol adenophostin has been achieved. Key features of the synthetic strategy to produce a triol for phosphorylation include a new selective mono-tosylation of racemic 1,2:4,5-di-*O*-isopropylidene-*myo*-inositol using tosyl imidazole; subsequent conversion of the product into separable camphanate ester derivatives, one leading to a chiral *myo*-inositol triflate used as a synthetic building block and the other to l-1-*O*-methyl-*myo*-inositol [l-(+)-bornesitol] to assign the absolute configuration; the nucleophilic coupling of an alkoxide of a ribose pent-4-ene orthoester unit with a structurally rigid chiral *myo*-inositol triflate derivative, representing the first *sec*–*sec* ether formation between a cyclitol and ribose. Reaction of the coupled product with a silylated nucleobase completes the assembly of the core structure. Further protecting group manipulation, mixed *O*- and *N*-phosphorylation, and subsequent removal of all protecting groups in a single step achieves the final product, avoiding a separate *N*6 protection/deprotection strategy. d-*chiro*-Inositol adenophostin evoked Ca^2+^ release through IP_3_Rs at lower concentrations than adenophostin A, hitherto the most potent known agonist of IP_3_Rs.

## Abstract 2

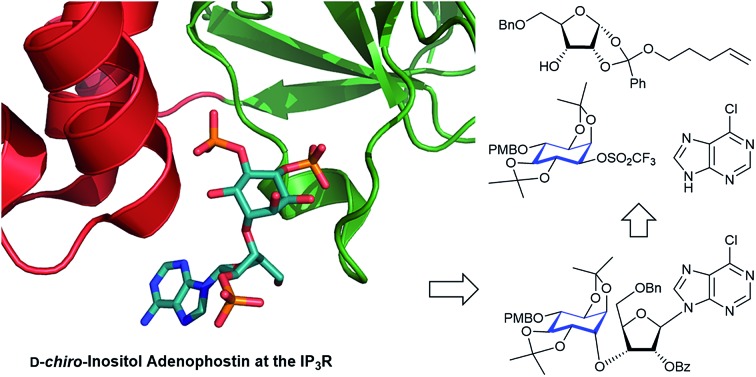
A densely functionalised phosphorylated *chiro*-inositol-nucleoside ether conjugate constructed from cyclic fragments is the most potent IP_3_ receptor ligand discovered.

## Introduction

**Fig. 1:**
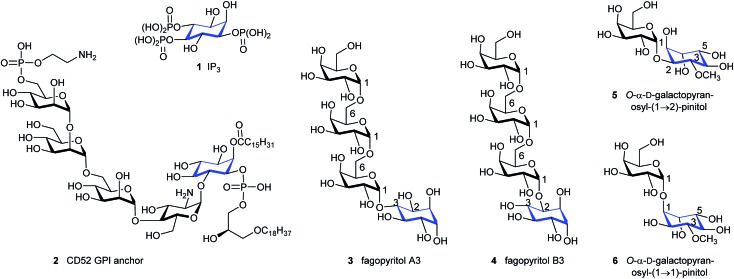
d-*myo*-Inositol 1,4,5-trisphosphate (IP_3_) **1**, CD52 GPI anchor **2** and natural products **3–6** containing d-*myo*- and d-*chiro*-inositol sugar conjugates (inositol component shown in blue).

Inositols are present in cells from every domain of life (archaea, bacteria and eukaryotes), with *myo*-inositol the most abundant of the nine inositol stereoisomers.^1^ Polyphosphorylated derivatives of *myo*-inositol are common in biology and have many functions,^2^ including roles in regulating ion-channels,^3^ phosphate levels,^4^ metabolic flux,^5^ transcription, mRNA export and translation,^6^ insulin signalling, embryonic development^7^ and stress responses.^8^d-*myo*-Inositol 1,4,5-trisphosphate (IP_3_, **1**, Fig. 1), which is an intracellular messenger that evokes Ca^2+^ signals after binding to its own receptor (IP_3_R)1a,^9^ and higher *myo*-inositol polyphosphates are subjects of intense interest.1a,^10^ Such compounds are implicated in diverse areas of biology and disease.^11^*myo*-Inositol is also a component of glycosyl-phosphatidylinositol (GPIs, see compound **2**, Fig. 1),^12^ which anchor proteins to the cell surface and are present in all eukaryotic cells and some bacteria.^13^ Other stereoisomers of inositol also have biological activities,1*a* but these are less common or have not been widely investigated.^14^ Beyond GPIs, derivatives of *myo*-inositol can make simple conjugated derivatives with sugars; these are much less common and only a few natural products are known. In general, an equatorial hydroxyl group of an inositol is linked to the glycosidic C1 of a sugar. Examples of the rarer inositol derivatives are fagopyritol (**3** & **4**) and pinitol derivatives (**5** & **6**) which contain a d-*chiro*-inositol-galactose conjugate, and are isolated from various plant sources (Fig. 1).^15^ Most of the conjugates have a galactose unit connected *via* its glycosidic C1 to an equatorially configured oxygen at either C3 (**3**), C2 (**4** & **5**) or C5 (not shown) of the d-*chiro*-inositol unit, with further conjugates sometimes added at the primary C6 hydroxyl group of galactose (see compounds **3** & **4**, Fig. 1). Only one example was found where the axial configured oxygen at C1 of d-*chiro*-inositol forms an axial/axial configured glycosidic bond (see compound **6**, Fig. 1).

**Fig. 2:**
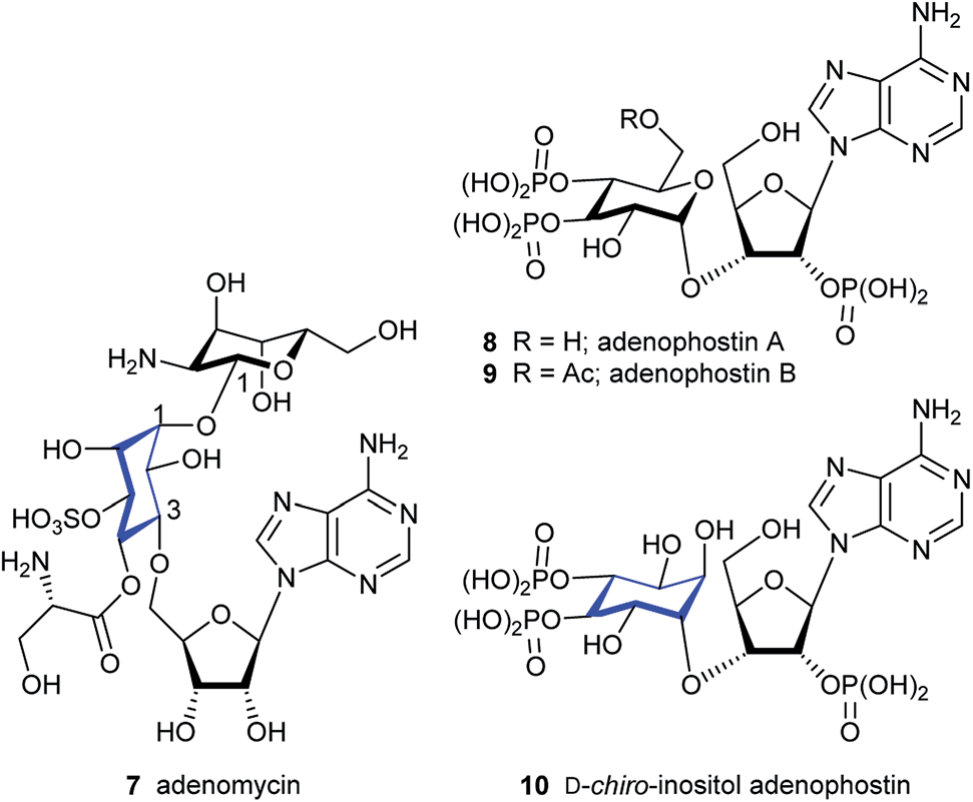
Adenomycin **7**, adenophostin A **8**, adenophostin B **9** and d-*chiro*-inositol adenophostin **10**.

Complex, but feasible, synthetic routes to such compounds and GPIs require conjugation at the sugar C1 glycosidic centre. GPIs are sugar conjugates, where a d-*myo*-inositol derivative is usually oxygen-linked from its C6 position to a d-glucosamine unit at its glycosidic C1 position. A phospholipid moiety at C1 of the d-*myo*-inositol unit enables GPIs to insert into the plasma membrane. The difficulty of isolating and purifying GPIs from biological sources lead to the development of methodologies for the synthesis of GPIs, GPI-anchored proteins and glycoproteins.^16^–^19^ To our knowledge, *sec*–*sec* inositol-hexose sugar conjugates are rare, apart from those at the glycosidic centre. Only one example of a pseudo-disaccharide could be found^20^ and there were no examples of inositol-pentose sugar conjugates. However, one example of a *prim-sec* ether linkage is known, for the antibiotic adenomycin **7** (Fig. 2).^21^ This compound contains one adenosine unit linked through its primary C5′ hydroxyl group to the C3 position of an l-*chiro*-inositol derivative to form a *prim-sec* ether bond. Additionally, a hemi-acetal is located at the glycosidic C1 of an l-gulosamine linked to the l-*chiro*-inositol C1. Interestingly, contrary to glycosidic connections in GPIs and in **3–6**, the latter linkage in **7** is reported to be β-glycosidic.^21^ Given the extensive biology of *myo*-inositol sugar conjugates and growing interest in the other eight inositol isomers,1*a* there is a need to generate analogues to construct *prim-sec* or more particularly *sec*–*sec* ether linkages. In the latter case, an example of particular interest can be drawn from the Ca^2+^-signalling field in the form of the adenophostins **8** & **9** (Fig. 2).^22^ These are naturally occurring fungal metabolites with a phosphorylated glucose component that resembles part of the IP_3_ pharmacophore and which are more potent than IP_3_**1** in evoking Ca^2+^ release through IP_3_ receptors (IP_3_Rs).

**Fig. 3:**
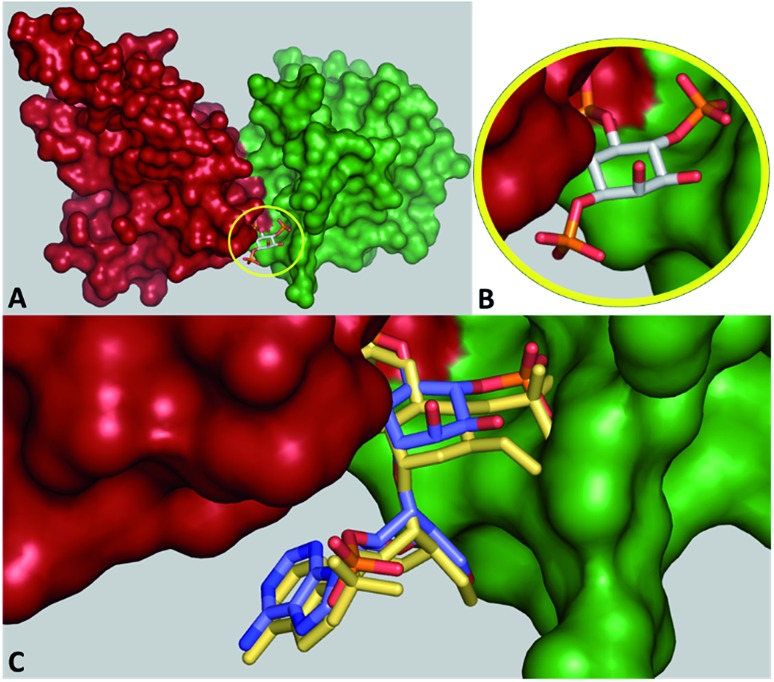
(A) IP_3_-binding core (IBC) of IP_3_ receptors with IP_3_**1** bound (PDB ; 1N4K). The IBC-α is shown in red, the IBC-β in green and IP_3_**1** in stick representation (carbon atoms in grey). (B) Close-up view of (A). (C) Model of d-*chiro*-inositol adenophostin **10** (carbons in light blue stick representation) with adenophostin A **8** (all atoms in yellow stick representation) binding to the IBC.

IP_3_Rs are intracellular Ca^2+^ channels that allow IP_3_, produced when extracellular stimuli promote hydrolysis of phosphatidylinositol 4,5-bisphosphate, to release Ca^2+^ from intracellular stores.^23^ The resulting Ca^2+^ signals regulate diverse cellular activities.^24^ Opening of the Ca^2+^-permeable channel of the IP_3_R is initiated when IP_3_ binds to the IP_3_-binding core (IBC, residues 224–604) of each of the four subunits of the tetrameric IP_3_R (Fig. 3A and B).^25^,^26^ Structure–activity analyses,^27^ structures of *N*-terminal fragments of IP_3_R^25^,^28^,^29^ and of complete IP_3_R,9*b* alongside mutagenesis studies have established that IP_3_ initiates IP_3_R activation by causing partial closure of the clam-like IBC. The 1- and 5-phosphates of IP_3_ interact primarily with residues in the α-domain of the IBC, while the 4-phosphate interacts with IBC-β (Fig. 3A). Adenophostin A (**8**) and adenophostin B (**9**) bind to IP_3_R with greater affinity than IP_3_ and they are ∼6–10-fold more potent than IP_3_ in evoking Ca^2+^ release.^30^,^31^ Hitherto, adenophostin A is the most potent known agonist of IP_3_R.^31^ Our model for the interaction of adenophostin A with the IBC,^32^ which is supported by mutagenesis^33^ and SAR analyses,^34^,^35^ suggests that the glucose 3′′,4′′-bisphosphate motif of adenophostin A mimics the 4,5-bisphosphate motif of IP_3_, while the 2′′-hydroxyl and 2′-phosphate of adenophostin A mimic the non-essential 6-hydroxyl and 1-phosphate groups of IP_3_.^25^,^36^ A recently published cryo-EM structure of IP_3_R1 with and without adenophostin A bound^37^ hints at substantially different binding modes for adenophostin A. However, the resolution of ligands in such structures is far from optimal and the ligand structure here is chemically incorrect.

We focused on developing a synthetic route to “d-*chiro*-inositol adenophostin” **10**. The aim was to replace the α-glucopyranosyl unit of **8** with the most similarly-configured inositol unit (d-*chiro*-inositol, acting substantially as a pseudo-sugar). The resulting ligand would allow us to study any potentially novel effects of this modification on Ca^2+^ release. Since the phosphorylated inositol-nucleoside hybrid closely resembles adenophostin A, it would establish whether nature evolved a phosphorylated glucose moiety as the best mimic of the inositol bisphosphate motif or whether the activity of adenophostin A can be still further improved by replacing glucose with a motif more similar to the cyclitol in IP_3_. Finally, and importantly, if high adenophostin A-like potency were achieved in **10**, the hybrid would offer an axial hydroxyl group at the d-*chiro*-inositol C6 position (C2 position in *myo*-inositol), not present in adenophostin A. This position could be modified (*e.g. via* extended substituents) to target the cleft between the IBC domains (Fig. 3B) and might facilitate the design of high-affinity ligands as potential antagonists by virtue of their ability to prevent closure of the IBC clam. Such a strategy cannot be envisaged for adenophostin A.

Examining all the bonds that need to be formed to assemble **10**, it became clear that its total synthesis would be challenging, especially the formation of the *sec*–*sec* ether bond between the inositol ring and the ribose sugar. Linkages are usually made between a reactive sugar glycosyl acceptor at the 1-position and inositol, both in protected form, to give inositol-sugar conjugates after deprotection (Fig. 1). Non-glycosidic conjugates of this type are quite rare. However, some years ago in the synthesis of a pseudo-disaccharide a cyclitol derivative was used as the nucleophile and a triflate on a rigid sugar unit as the electrophile.^20^ This strategy would not easily work in our case because a d-*chiro*-inositol derivative would need to react as a nucleophile ideally with a triflate at the 3-position of a xylose derivative. S_N_2 reactions using 3-trifluoromethanesulfonyl- and 3-tosyl-xylose derivatives are described, but coupled products are only achieved when using non-alkaline nucleophiles like azide or thiophenolate.^38^,^39^ With the hydrogen at C4 in xylose placed almost anti-periplanar to the leaving group, the less reactive 3-tosyl derivative undergoes mainly elimination to form an olefin when reacted with alkoxides, but cleavage of the tosyl group is also observed.^39^

We report here a divergent strategy for the synthesis of **10** that includes the first *sec*–*sec* ether formation between a cyclitol and a ribose sugar as one key feature. Others are a selective mono-tosylation of a racemic inositol diol derivative and subsequent conversion of the product into diastereoisomeric camphanate esters separable by column chromatography. One diastereoisomer provides the chiral inositol triflate to be used as a building block, and the other provides a route to l-(+)-bornesitol in order to confirm absolute configurations. Coupling of the inositol triflate with the ribose alkoxide of a pent-4-ene orthoester derivative and subsequent reaction with a silylated nucleobase assembles the core structure for phosphorylation. Pan-phosphorylation of all hydroxyl and amino groups and removal of all protecting groups inventively in a single step affords the final product **10** without recourse to an adenine *N*6-protection strategy.

## Results and discussion

### Chemistry

**Scheme 1:**
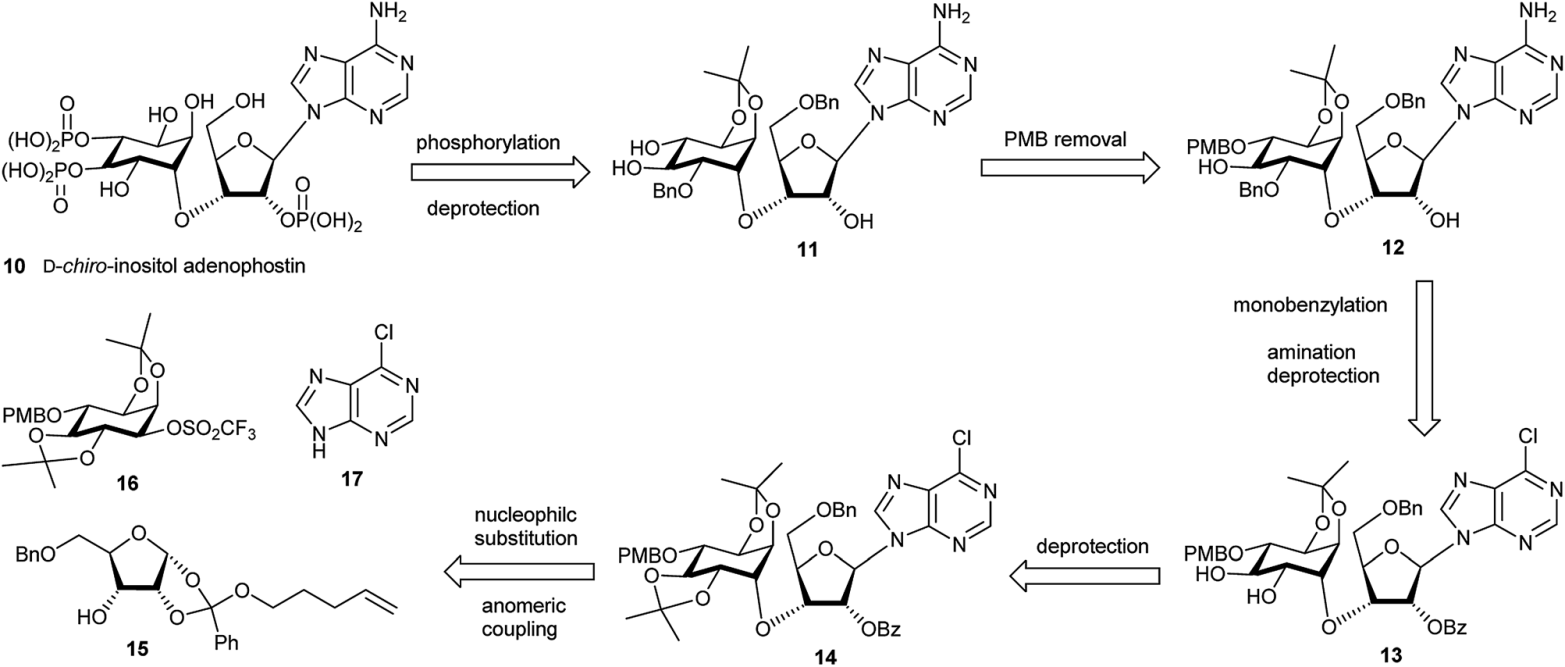
Retrosynthetic analysis of d-*chiro*-inositol adenophostin **10**.

**Fig. 4:**
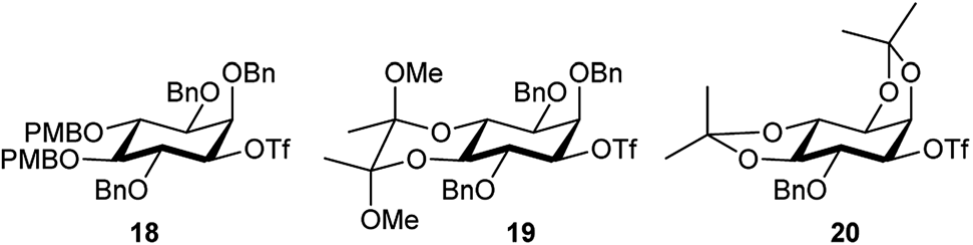
Triflates that undergo elimination.

The retrosynthetic analysis of d-*chiro*-inositol adenophostin **10** (Scheme 1), leads to **14** as the first compound containing all three components, d-*chiro*-inositol, ribose and a purine base as derivatives. An initial analysis for the formation of the *sec*–*sec* ether suggested that *myo*-inositol triflates may be used. Three such potential triflates were synthesised in turn, with increasing rigidity of the inositol ring (**18–20**, Fig. 4).

Initially, although triflate **18** in the chair conformation did not have an anti-periplanar H–Ins–OTf motif, the triflate eliminated to form a double bond in the presence of an alkoxide, demonstrating that the inositol framework needed to be more rigid. ^13^C NMR assignment indicated a quaternary carbon derived from proton abstraction located at *δ*_C_ = 154.1 and an olefinic C–H at *δ*_C_ = 97.0. The rigidity of the inositol ring was further increased to give derivative **19**. However, elimination in the presence of base was still observed and similar olefinic fingerprint signals in the ^13^C NMR were seen as above. A still more constrained structure was an obvious further choice, so no elimination should occur. A third compound **20**, provided a more rigid inositol triflate and the crude product from alkoxide treatment was purified by column chromatography. However, elimination still occurred and two products were observed in the ^1^H NMR spectrum. After a number of inositol triflates eliminated to form cyclic vinyl ethers, triflate **16** (and the most difficult to manipulate to the final product) was prepared (Scheme 5) and successfully used as a functionalised chiral *myo*-inositol intermediate for the preparation of the d-*chiro*-inositol derivative **14**. An inositol triflate with a similar protecting group pattern has been previously reported in S_N_2 reactions. However, only non-basic nucleophiles such as fluoride, azide and thioacetate were used, but no alkoxides.^40^

Although the formation of *sec*–*sec* ethers is well known, only a few examples lead to densely functionalised compounds.^20^,^41^,^42^ The main problem in the required S_N_2 type reaction is the use of highly substituted, sterically hindered starting materials. A limited number of electrophiles, such as a triflate within a rigid organic framework have been used successfully.^20^,^41^ Compound **14** could be assembled in two steps from the ribose orthoester derivative **15**. Note that although **15** is depicted as one isomer at the orthoester moiety, which is indeed the case, in fact the exact stereochemistry at the quaternary carbon has not been proven. Diverse attempts to derivatise **15** and **23** to give a crystalline product for X-ray studies were made, but all of these failed. Compound **15** was coupled to the l-*myo*-inositol triflate derivative **16**. Subsequent anomeric coupling of the orthoester group with silylated 6-chloropurine **17** gave compound **14**.

**Scheme 2:**
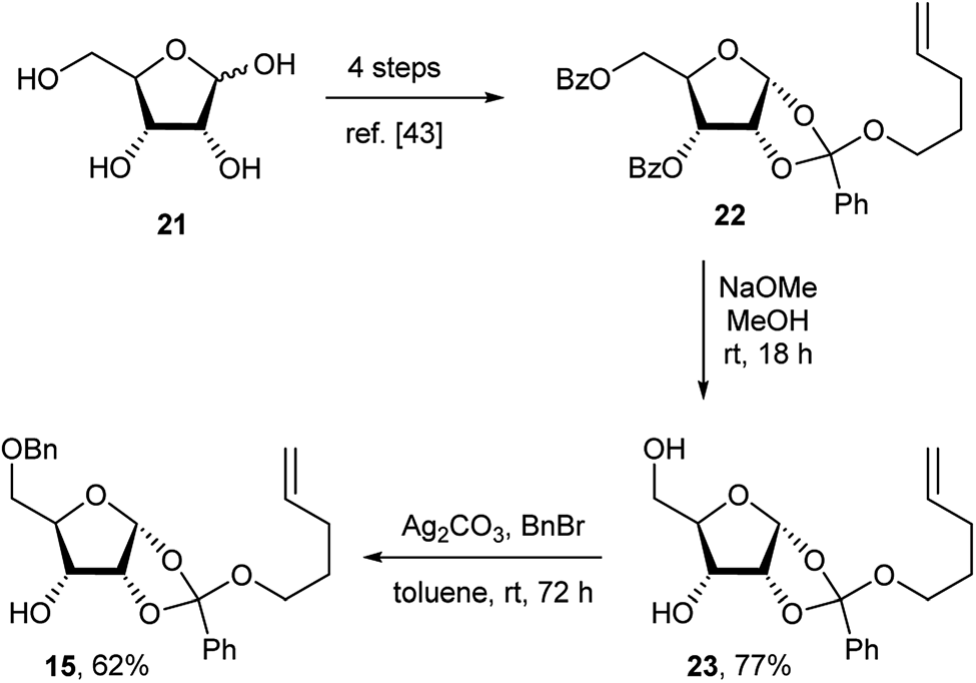
Synthesis of the d-ribose orthoester derivative **15**.

The ribose fragment of **10** was synthesised in four steps from d-(–)-ribose **21** to give the pent-4-ene orthoester **22**.^43^ Reaction of **22** with sodium methoxide in methanol furnished diol **23** in high yield.^43^ Selective benzylation of the primary hydroxyl group using silver carbonate and benzyl bromide in toluene gave compound **15** in good yield (Scheme 2).^44^

**Scheme 3:**
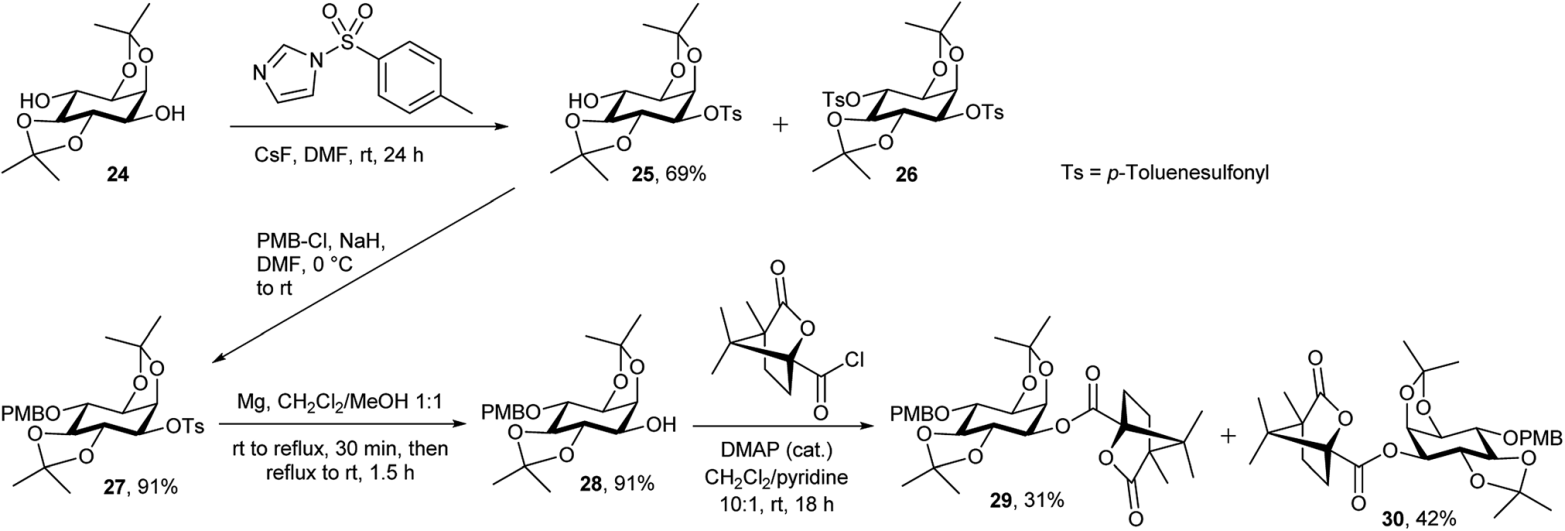
Synthesis of protected l-*myo*-inositol 3-*O*-camphanate **29** and d-*myo*-inositol 3-*O*-camphanate **30**.

**Scheme 4:**
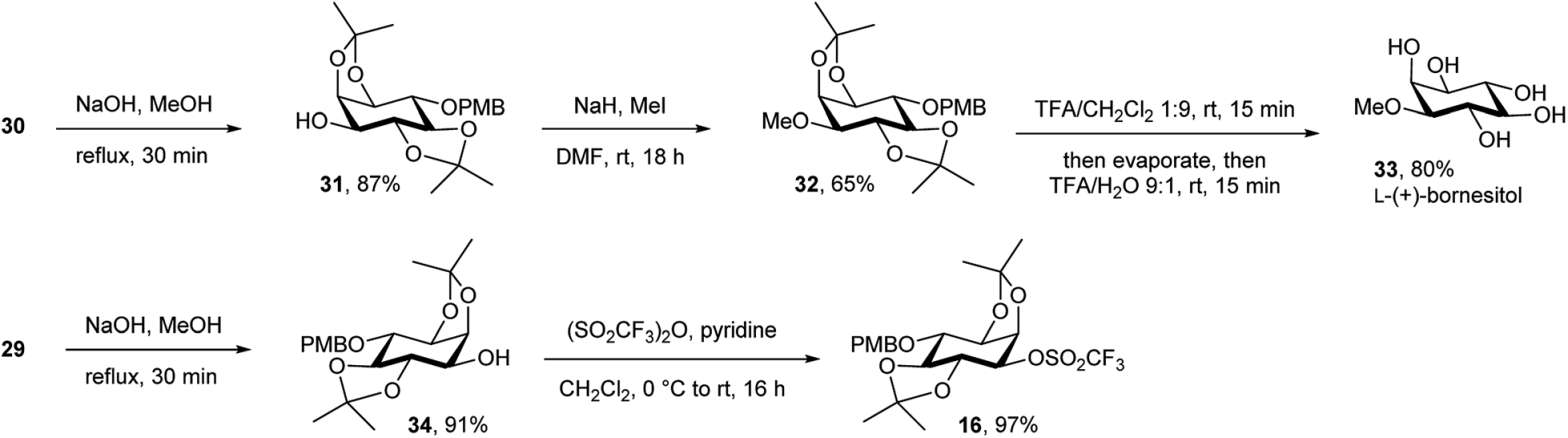
Synthesis of protected l-*myo*-inositol 3-*O*-triflate derivative **16** and l-(+)-bornesitol **33**.

Synthesis of the chiral l-*myo*-inositol triflate derivative **16** started from racemic 1,2:4,5-di-*O*-isopropylidene-*myo*-inositol **24**^45^ (Schemes 3 and 4). In order to convert an l-*myo*-inositol derivative into the d-*chiro*-inositol derivative *via* an S_N_2 reaction a triflate was required as a leaving group at C3. The hydroxyl group at C6 was protected as a *p*-methoxybenzyl (PMB) ether, but selective protection at the more reactive 3-hydroxyl group was required. Selective mono-tosylation of the hydroxyl group at C3 of **24** using tosyl chloride in pyridine lead to low yields of compound **25**.^46^ However, a new method was devised for this conversion using tosyl imidazole^47^ in the presence of cesium fluoride in DMF at room temperature. Compound **25** was obtained in good yield along with a minor amount of the bis-tosylated product **26**. Reaction of **25** with sodium hydride and *p*-methoxybenzyl chloride in DMF gave compound **27** in high yield. Subsequent removal of the tosyl group was achieved by dissolving **27** in dichloromethane followed by the addition of methanol and magnesium turnings.^48^ Compound **28** was treated with (1*S*)-(–)-camphanic chloride^49^ to afford diastereoisomers **29** and **30**, which were separated by column chromatography and both were isolated in good yield (Scheme 3).

The absolute configuration was determined using diastereoisomer **30**, which was converted in several steps *via* ester hydrolysis, methylation and protecting group removal *via***31** and **32** into the known natural product l-(+)-bornesitol **33**; the configuration of this product was confirmed by comparison to previously reported ^1^H NMR and optical rotation data.^50^ The chiral *myo*-inositol triflate **16** was then synthesised in good overall yield from **29** by ester hydrolysis and reaction of chiral alcohol **34** with triflic anhydride and pyridine in dichloromethane (Scheme 4).

**Scheme 5:**
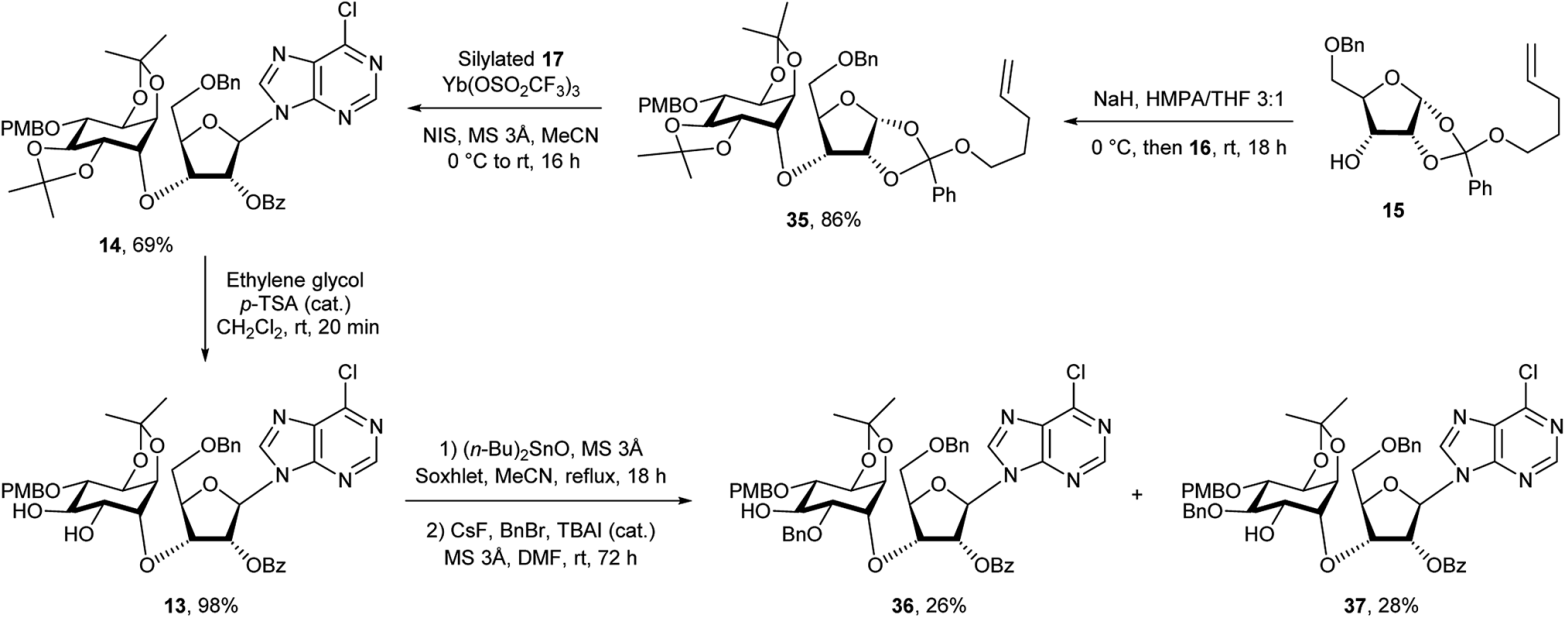
Synthesis of mono-benzylated compound **36**.

Compound **15** was first dissolved in anhydrous THF/HMPA before adding sodium hydride to generate the alkoxide. The chiral triflate **16** was then added to the alkoxide to give **35** in 86% yield, transforming the l-*myo*-inositol unit into the desired d-*chiro*-inositol derivative by inversion of configuration at C3. DMPU could also be used to replace HMPA as a co-solvent, but gave overall slightly lower yields (about 10% less). However, no reaction was observed when DMF alone was used as a solvent. Subsequent coupling of **35***via* its pent-4-ene orthoester group with silylated 6-chloropurine^51^ in the presence of an activator under anhydrous conditions gave compound **14** in good yield.43*b* The *trans*-isopropylidene group was then selectively removed by treatment with ethylene glycol and *p*-toluenesulfonic acid in dichloromethane for no longer than 20 minutes to give compound **13** in almost quantitative yield. After various attempts, we found that a one-pot reaction procedure strategy was unsuitable to mono-benzylate compound **13**, since the harsh reaction conditions resulted in complex mixtures and the desired compound **36** was not observed.^52^ Selective mono-benzylation of **13** was best achieved in a two-stage procedure.^53^ First, **13** was reacted with *n*-Bu_2_SnO in refluxing acetonitrile using a Soxhlet condenser containing activated 3 Å MS. The resulting tin acetal was isolated and directly converted into the desired mono-benzylated compound **36** in 26% yield using benzyl bromide, cesium fluoride and tetra-*n*-butylammonium iodide in anhydrous DMF. However, no regioselectivity was observed and the other mono-benzylated compound **37** was isolated in a similar yield together with unconverted starting material **13** (Scheme 5).

**Scheme 6:**
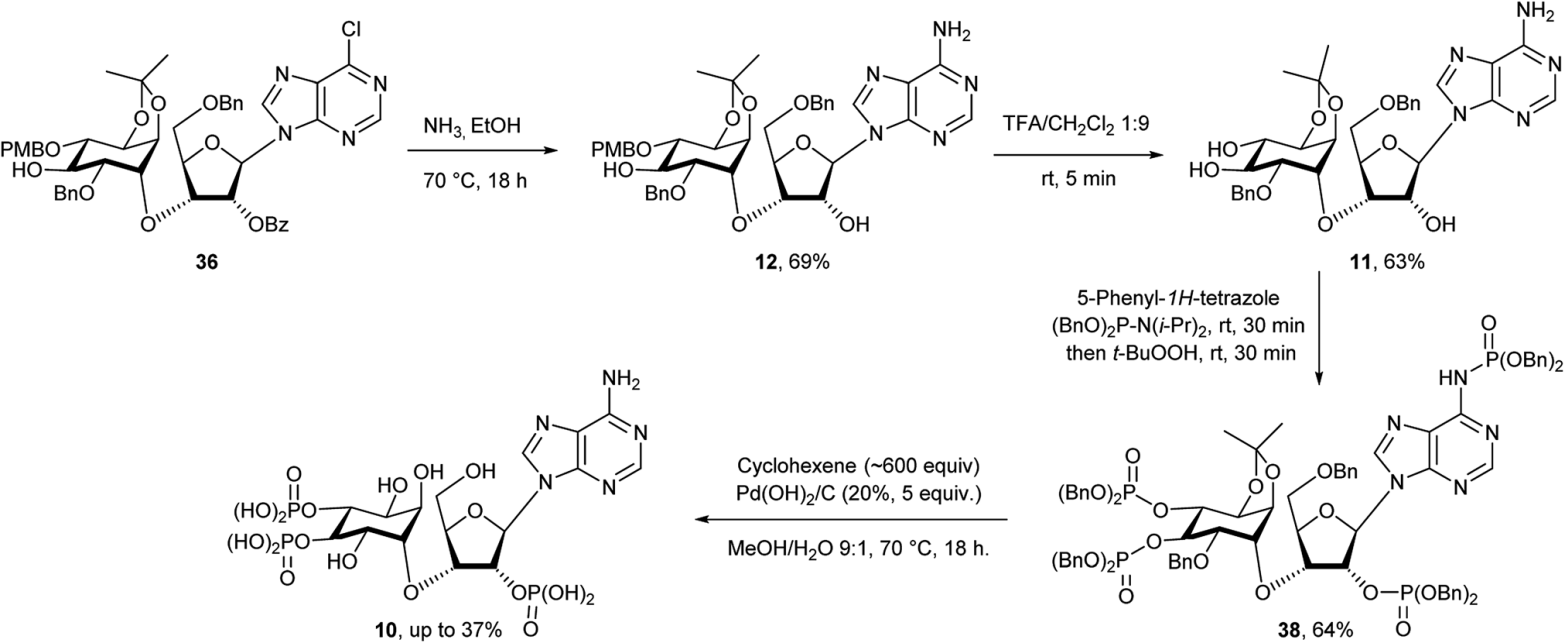
Synthesis of d-*chiro*-inositol adenophostin **10**.

Small samples of both chromatographically separable regioisomers **36** and **37** were treated with TFA/DCM to remove the *p*-methoxybenzyl group and the products then analysed by ^1^H NMR and COSY without and with the addition of D_2_O. All hydroxyl protons and the corresponding adjacent cyclitol ring protons were identified. The 1,2-diol relationship was indicated by a strong coupling between these two adjacent ring protons. For the 1,3-diol relationship that coupling was not found. Compound **36** led to a 1,2-diol, whereas **37** led to a 1,3-diol. Therefore, **36** was identified as the required 2-*O*-benzylated derivative that was then treated with ammonia in ethanol at 70 °C to give **12** in 69% yield with concomitant removal of the benzoyl ester (Scheme 6). After trying various methods we found that removal of the PMB protecting group was best achieved using TFA in anhydrous dichloromethane for no longer than 5 minutes.^54^ Longer reaction times and/or traces of moisture usually lead to increasing amounts of more polar by-products where the *cis*-isopropylidene protecting group had also been removed. For the final phosphorylation of triol **11** we had originally envisaged use of imidazolium triflate^33^,35d as a means of achieving *O*-selective phosphitylation over *N*-phosphitylation. While this approach has been used well in the past, we found it to be very cumbersome for the phosphorylation of low milligram quantities of triol. It was not possible to achieve complete selective phosphitylation and to completely exclude water, then titrate in the required amount of phosphitylating reagent without achieving substantial amounts of concomitant *N*-phosphitylation, although products from this could be easily separated. We therefore reasoned that since *N*-phosphates have been reported to be labile under acidic and/or hydrogenation reaction conditions^55^ such a contaminant should therefore convert into the free amino group during the final step of our synthesis. This idea thus argued for a pan-phosphitylation approach, whereby **11** was first phosphorylated with an excess of reagent that easily leads to the tris-*O*-phosphorylated mono-*N*-phosphorylated product that could be converted to the mixed tris-phosphate/mono-phosphoramidate **38** upon oxidation. To illustrate this further, the conversion of **11** to the fully deblocked **10** was carried out on a <10 mg scale. Compound **11** was treated with 5-phenyl-1*H*-tetrazole and dibenzyl diisopropylphosphoramidite^56^ in dichloromethane followed by oxidation of the product with *t*-butyl hydroperoxide to achieve the fully protected compound **38** in good overall yield (Scheme 6). The *N*-phosphate group was readily distinguished from the *O*-phosphate by its broad ^31^P NMR resonance peak at *δ* –0.9 in comparison to sharp peaks at *δ* –1.2 and –1.5 (two overlapping peaks) respectively. Subsequent treatment of **38** with hydrogen generated from cyclohexene in the presence of palladium hydroxide on carbon in methanol and water at 70 °C overnight led to an acidic reaction mixture (pH 3), as all *O*- and *N*-benzylated phosphate groups were converted to their free acids. Subsequently, this also lead to removal of the *cis*-isopropylidene group and degradation of the *in situ* generated acid-labile free *N*-phosphate by nucleophilic cleavage of its P–N bond.^55^ As a pleasing result, therefore, all different types of protecting group were removed in a single step to give d-*chiro*-inositol adenophostin **10** (Scheme 6). Yields of **10** were routinely quite variable and possibly complicated by retention of compound on the hydrogenation catalyst. The best yield achieved so far is 37%. We imagine that this mixed *O*- and *N*-phosphitylation methodology, avoiding a sometimes cumbersome extra *N*-protection strategy, will find application in many nucleotide synthesis situations where only small amounts of compound are available. For biological evaluation, **10** was purified by semi-prep HPLC (purity > 99%) and quantified by UV spectroscopy. Final structural characterisation was additionally confirmed by ^1^H–^31^P NMR correlation (see ESI†).

### Biology

**Fig. 5:**
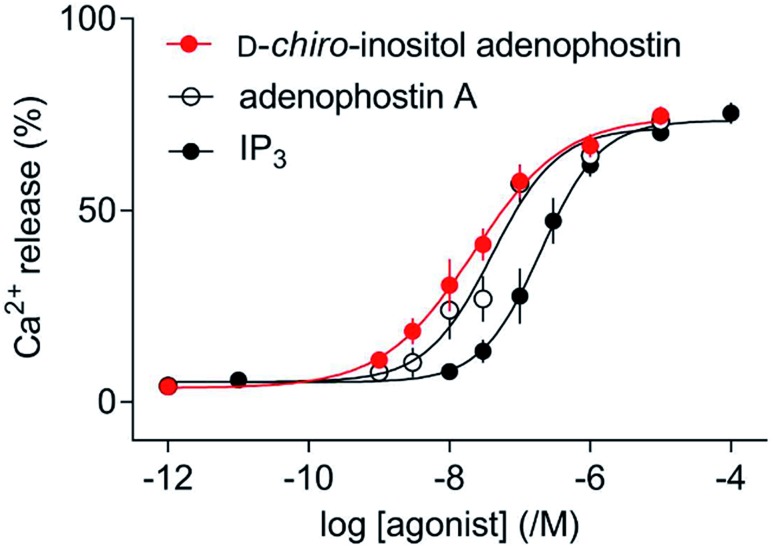
d-*chiro*-Inositol adenophostin **10** is more potent than adenophostin A **8** in evoking Ca^2+^ release through IP_3_R. Results (%, means ± SEM, *n* = 6) show the Ca^2+^ release evoked by the indicated concentrations of IP_3_**1**, adenophostin A **8** or d-*chiro*-inositol adenophostin **10** from the intracellular stores of permeabilized HEK cells expressing IP_3_R1.

d-*chiro*-Inositol adenophostin **10** was investigated as an agonist at the IP_3_R using an intracellular Ca^2+^ mobilisation assay in comparison to adenophostin A **8** and IP_3_**1**. We used a low-affinity Ca^2+^ indicator within the intracellular stores of permeabilized HEK cells expressing IP_3_R1 to record Ca^2+^ release evoked by synthetic ligands. Maximally effective concentrations of IP_3_, adenophostin A **8** or d-*chiro*-inositol adenophostin **10** caused release of a similar fraction (∼70%) of the Ca^2+^ sequestered by intracellular stores. However, adenophostin A **8** and **10** were 5.3 and 8.9-fold more potent than IP_3_, respectively. Moreover, d-*chiro*-inositol adenophostin **10** was 1.7-fold more potent than adenophostin A **8** (Fig. 5 and Table 1).

**Table 1:** Effects of IP_3_, adenophostin A **8** and d-*chiro*-inositol adenophostin **10** on Ca^2+^ release from the intracellular stores of permeabilized HEK-IP_3_R1 cells^*a*^

Compound	Ca^2+^ release			
	pEC_50_	EC_50_ (nM)	(%)	*h*
IP_3_ (**1**)	6.73 ± 0.13	186	73 ± 2	1.2 ± 0.1
Adenophostin A (**8**)	7.45 ± 0.16	35	71 ± 3	1.5 ± 0.4
d-*chiro*-Inositol adenophostin (**10**)	7.67 ± 0.14	21	72 ± 3	1.4 ± 0.6

^*a*^Results are means ± SEM (Ca^2+^ release (%), Hill coefficient (*h*) and pEC_50_) or means (EC_50_, half maximal effective concentration) from six independent experiments each performed in duplicate. There were no significant differences between the ligands in the values for Ca^2+^ release (%) or *h*. The pEC_50_ values were significantly different (*P* < 0.05), for IP_3_*vs.* adenophostin A, IP_3_*vs.*d-*chiro*-inositol adenophostin and d-*chiro*-inositol adenophostin *vs.* adenophostin A.

## Conclusion

In summary, a concise synthesis of d-*chiro*-inositol adenophostin has been achieved. Key features of the strategy include a new selective mono-tosylation reaction of a racemic *myo*-inositol diol derivative and subsequent elaboration of the product into separable camphanate derivatives of a fully protected intermediate. One such diastereoisomer is converted to l-(+)-bornesitol to confirm the absolute configuration and the other leads to the required chiral *myo*-inositol triflate that is used as a synthetic building block. Critical formation of a *sec*–*sec* ether used a rigidly structured chiral inositol triflate with an alkoxide of a suitable protected ribose derivative; subsequent reaction of the fully protected coupled product with a silylated nucleobase assembled the core structure and base amination and protecting group manipulations finally afforded a triol for phosphorylation. After phosphitylation of both the adenine amino group and inositol hydroxyl groups, removal of all protecting groups was accomplished in a single step to afford the final product. Thus, replacement of the α-glucopyranosyl unit in adenophostin A with the equivalent d-*chiro*-inositol motif leads to the trisphosphate d-*chiro*-inositol adenophostin **10**. **10** Was evaluated as an agonist for intracellular Ca^2+^ release through the IP_3_R and its EC_50_ of 21 nM was lower than that of **8** (35 nM). It is the most potent agonist of IP_3_Rs so far identified and may offer new opportunities to develop high-affinity antagonists of IP_3_R.

## Conflicts of interest

There are no conflicts to declare.

## Supplementary Material

Supplementary information
