# Supplementary material for: A synthetic cyclitol-nucleoside conjugate polyphosphate is a highly potent second messenger mimic
Source: Chem Sci. 2019 Apr 23;10(20):5382–90. doi: 10.1039/c9sc00445a (PMC6540904; doi:10.1039/c9sc00445a)
Supplement: Supplementary file 1 [file SC-010-C9SC00445A-s001.pdf]

Electronic Supplementary Material (ESI) for Chemical Science.
This journal is © The Royal Society of Chemistry 2019

Supplementary Information

A synthetic cyclitol-nucleoside conjugate polyphosphate is a highly potent second messenger mimic

Wolfgang Dohle,[a¶] Xiangdong Su,[a¶] Stephen J. Mills,[a¶] Ana M. Rossi,[b] Colin W. Taylor,[b] and Barry V. L. Potter[a]

[a]*Medicinal Chemistry & Drug Discovery, Department of Pharmacology, University of Oxford, Mansfield Road, Oxford OX1 3QT, UK.*

[b]Department *of Pharmacology, University of Cambridge, Tennis Court Road, Cambridge CB2 1PD, UK.*

[¶]*Equal Contribution*

Table of Contents

**Chemistry**	S2-S20
**NMR Spectra**	S21-S42
**Biology**	S43-S44
**Additional References**	S45

Chemistry

General Experimental. Unless otherwise noted, reactions were carried out in round-bottom flasks with magnetic stirring. Analytical thin layer chromatography (TLC) was performed with silica gel 60 F254 aluminium plates (Merck). Visualization was done under a UV lamp (254 nm) and by immersion in ethanolic phosphomolybdic acid (PMA) or aqueous potassium permanganate ($KMnO_4$), followed by heating using a heat gun. Organic solutions were concentrated by rotary evaporation at 40–50 °C. Flash column chromatography was performed on a CombiFlash Rf Automated Flash Chromatography System (Teledyne Isco, Lincoln, NE, USA) equipped with a UV detector, using RediSep Rf disposable silica gel columns or high performance GOLD silica columns.

Materials. All chemicals were purchased either from Sigma-Aldrich (Gillingham, UK) or Alfa Aesar (Heysham, UK). Organic solvents of AR grade (PE, EtOAc, $CHCl_3$, acetone, MeOH, CH_2Cl_2) were supplied by Sigma-Aldrich (Gillingham, UK) or Fisher Scientific (Loughborough, UK) and used as supplied. The petroleum ether (PE) used for column chromatography was of fractions 40−60 °C. Anhydrous solvents (Hexamethylphoshphoramide (HMPA), *N,N*-dimethylformamide (DMF), tetrahydrofuran (THF), toluene, pyridine, acetonitrile (MeCN), CH_2Cl_2, EtOH), were purchased from Sigma-Aldrich and stored under a positive pressure of N_2 after use. Super dry CH_2Cl_2 was obtained by distillation from P_2O_5 and storage over 3A molecular sieves (10% m/v) for at least 3 days.[1] D/L-1,2:4,5-diisopropylidene-*myo*-inositol, 1-(*p*-Toluenesulfonyl)-imidazole, (1S)-(-)-camphanic chloride and trimethylsilyl 6-chloropurine were synthesised according to literature procedures.[2-6]

Instrumentation. ^{1}H, ^{31}P and ^{13}C NMR spectra were recorded with a Bruker AVIII HD 400 spectrometer at 400, 162 and 100 MHz, respectively. ^{1}H and ^{31}P NMR spectra of compound **10** were recorded with a Bruker AVIII HD 500 spectrometer at 500 and 202 MHz, respectively. Chemical shifts are reported in parts per million (ppm) relative to the solvent residual peaks as internal standards. ^{1}H NMR: 7.26 ppm ($CDCl_3$); 2.05 ppm (acetone-d_6); 2.50 ppm

(DMSO-d_6); 3.31 ppm (methanol-d_4); 4.79 (D_2O). ^{13}C NMR: 77.16 ppm ($CDCl_3$); 29.84 ppm (acetone-d_6); 39.52 ppm (DMSO-d_6). Phosphorus chemical shifts are reported in ppm relative to an 85% H_3PO_4 external standard. Melting points were determined using a Stanford research systems Optimelt MPA100 melting point apparatus (Stanford Research Systems, Sunnyvale, CA, USA), and are uncorrected. Optical rotations were recorded on a Schmidt Haensch Unipol polarimeter in a cell with a path length of 1 dm (using the sodium D line, 589 nm). [α]D values are given in 10^{-1} deg cm^2 g^{-1}. Concentrations are reported in g/100 mL. Temperatures are reported in °C. High resolution time-of-flight mass spectra was performed on an Agilent single quadrupole with CTC-PAL autosampler or a Bruker Daltonics microTOF mass spectrometer using electrospray ionisation (ESI).

Experimental Procedures and Characterisation Data.

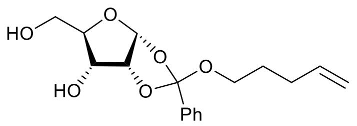

23: *β*-D-Ribofuranose-1,2-(pent-4-enyl orthobenzoate). Compound **22** (4.12 g, 7.8 mmol) was dissolved in MeOH (100 mL) and sodium methoxide (335 mg, 6.2 mmol) was added. The mixture was stirred at rt for 18 h. Carbon dioxide was passed through the solution for 30 min. The mixture was concentrated in vacuum and the resulting white residue was purified by flash column chromatography (CH_2Cl_2 and 1% Et_3N to CH_2Cl_2/EtOAc 3:2 and 1% Et_3N) to afford compound **23** as a colourless viscous oil (1.943 g, 77%). ^1H NMR (400 MHz, acetone-d_6): δ = 7.73-7.67 (2H, m, 2 x CH), 7.40-7.35 (3H, m, 3 x CH), 6.02 (1H, d, J = 3.9 Hz, H-1), 5.88-5.75 (1H, m, CH), 5.01 (1H, ddd, J = 17.2, 3.6, 1.7 Hz, CH), 4.93 (1H, ddt, J = 10.3, 2.1, 1.2 Hz), 4.85 (1H, t, J = 4.4 Hz), 4.02-3.96 (1H, m), 3.76-3.67 (2H, m), 3.58-3.48 (3H, m), 3.46 (2H, dt, J = 6.4, 1.8 Hz), 2.12 (2H, q, J = 7.3 Hz), 1.63 (2H, quin, J = 7.0 Hz).[7,8]

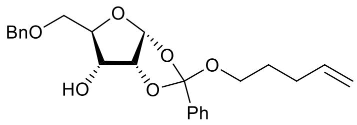

15: 5-*O*-Benzyl-*β*-D-ribofuranose-1,2-(pent-4-enyl orthobenzoate). Compound **23** (1.94 g, 6.0 mmol) was dissolved in toluene (30 mL). Then silver carbonate (5.07 g, 18.0 mmol) and benzyl bromide (2.05 g, 12.0 mmol) were added. The mixture was stirred in the dark at rt for 24 h. Et_3N (3.0 mL) was added and the reaction mixture was filtered. The solid in the filter was washed with CH_2Cl_2 (200 mL) and added to the filtrate. The organic solution was washed with water (200 mL) and brine (100 mL), dried over Na_2SO_4 and concentrated in vacuum. Purification by flash column chromatography (PE and 1% Et_3N to PE/EtOAc 4:1 and 1% Et_3N) afforded compound **15** as a colourless viscous oil (1.54 g, 62%). $[\alpha]_D^{22}$ = +36.4° (c = 0.63; acetone). ^{1}H NMR (400 MHz, acetone-d_6): δ = 7.74-7.66 (2H, m), 7.41-7.36 (3H, m), 7.35-7.24 (5H, m), 6.06 (1H, d, *J* = 3.9 Hz), 5.88-5.74 (1H, m), 5.01 (1H, dd, *J* = 17.0, 1.7 Hz), 4.93 (1H, d, *J* = 10.3 Hz), 4.86 (1H, t, *J* = 4.3 Hz), 4.50 (2H, s), 4.17 (1H, D_2O exch, d, *J* = 8.4 Hz), 4.00-3.92 (1H, m), 3.73-3.65 (2H, m), 3.52 (1H, dd, *J* = 11.3, 5.6 Hz), 3.46 (2H, t, *J* = 6.4 Hz), 2.12 (2H, q, *J* = 7.3 Hz), 1.64 (2H, quin, *J* = 7.0 Hz). ^{13}C NMR (100 MHz, acetone-d_6): δ = 139.6 (C), 139.2 (CH), 139.1 (C), 129.7 (CH), 129.0 (CH), 128.7 (CH), 128.3 (CH), 128.2 (CH), 127.1 (CH), 124.2 (C), 115.1 (CH_2), 105.1 (CH), 81.1 (CH), 80.8 (CH), 73.6 (CH_2), 72.9 (CH), 70.0 (CH_2), 62.6 (CH_2), 31.0 (CH_2), 29.6 (CH_2). HRMS (ES): m/z 413.1948, $C_{24}H_{29}O_6^+$ $[M+H]^+$ requires 413.1959.

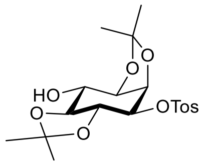

25: D/L-3-*O*-*p*-Toluenesulfonyl-1,2:4,5-di-*O*-isopropylidene-*myo*-inositol. 1-(*p*-Toluenesulfonyl)-imidazole (10.00 g, 45.0 mmol) was dissolved in DMF (60 mL) and compound **24** (11.72 g, 45.0 mmol) was added to the mixture which was stirred at rt. Cesium fluoride (6.84 g, 45.0 mmol) was then added and the mixture was stirred at rt under argon for 24 h. The solvent was evaporated in vacuum. The resulting syrup was partitioned between CH_2Cl_2 (400 mL) and water (400 mL) and the cloudy organic layer was filtered through a bed of celite

to give a colourless solution. Evaporation of the solvent provided a white solid. Purification by flash column chromatography (CH_2Cl_2/EtOAc 4:1) afforded compound **25** as a white solid (12.95 g, 69%). M.p. 157-159 °C, Lit.[9] 170-173 °C. ^{1}H NMR (400 MHz, DMSO-d_6): δ = 7.78 (2H, d, *J* = 8.4 Hz, 2 x CH), 7.43 (2H, d, *J* = 8.0 Hz, 2 x CH), 5.52 (1H, d, *J* = 4.7 Hz, OH), 5.03 (1H, dd, *J* = 10.3, 4.4 Hz, *H*-3), 4.05 (1H, t, *J* = 4.6 Hz, *H*-2), 3.87 (1H, t, *J* = 5.1 Hz, *H*-1), 3.74 (1H, t, *J* = 9.4 Hz, *H*-4), 3.52 (1H, ddd, *J* = 11.4, 10.9, 5.3 Hz, *H*-6), 3.38 (1H, dd, *J* = 10.6, 9.4 Hz, *H*-5), 2.38 (3H, s, CH_3), 1.29 (3H, s, CH_3), 1.25 (3H, s, CH_3), 1.22 (3H, s, CH_3), 0.99 (3H, s, CH_3). ^{13}C NMR (100 MHz, DMSO-d_6): δ = 144.9 (C), 133.6 (C), 129.8 (CH), 127.7 (CH), 129.8 (CH), 108.7 (CH), 82.0 (CH), 77.7 (CH), 77.7 (CH), 74.3 (CH), 73.1 (CH), 72.6 (CH), 27.8 (CH_3), 26.8 (CH_3), 26.5 (CH_3), 25.6 (CH_3), 21.0, (CH_3). Anal. Calc. for $C_{19}H_{26}O_8S$: C, 55.06; H, 6.32. Found: C, 55.1; H, 6.38.

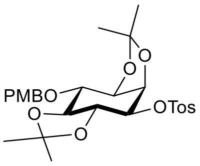

27: D/L-6-*O*-*p*-Methoxybenzyl-3-*O*-*p*-toluenesulfonyl-1,2:4,5-di-*O*-isopropylidene *myo*-inositol. Compound **25** (12.95 g, 31.2 mmol) was dissolved in DMF (150 mL). *p*-Methoxybenzyl chloride (5.5 mL, 38.1 mmol) was added followed by NaH (95 %, 1.01 g, 40.4 mmol). The reaction mixture was stirred at 0 °C for 30 min and then for 1 h at rt. Water (400 mL) was added to the mixture and the solid was filtered off. The solid was partitioned between CH_2Cl_2 (400 mL) and water (400 mL). The organic solution was filtered over a bed of celite, dried and the solvent was evaporated. The resulting solid was washed (EtOAc/PE 5:1) to give an off-white solid that was nearly pure. The bulk of the pale yellow compound was purified by crystallisation from EtOAc and the remaining yellow solid was purified by flash column chromatography (CH_2Cl_2 to CH_2Cl_2/EtOAc 20:1) to afford compound **27** as a white solid (15.30 g, 91 %) R_f = 0.32 (CH_2Cl_2/EtOAc 20:1); (m.p. 175-178 °C, from EtOAc/PE) Optimelt; (hot stage microscope m.p. 172-174 °C). Compound recrystallized from EtOAc/hexane (3:2) too. ^{1}H NMR (400 MHz, $CDCl_3$): δ = 7.83 (2H, d, *J* = 8.3 Hz, 2 x CH), 7.29 (2H, d, *J* = 8.4 Hz, 2 x CH), 7.28 (2H, d, *J* = 8.6 Hz, 2 x CH),

6.84 (2H, d, J = 8.7 Hz, 2 x CH), 4.75 (1H, dd, J = 10.4, 4.2 Hz, *H*-3), 4.69 (2H, s, OCH_2), 4.36 (1H, t, J = 4.6 Hz, *H*-2), 4.09 (1H, dd, J = 6.3, 5.6 Hz, *H*-1), 3.91 (1H, t, J = 9.5 Hz, *H*-4), 3.77 (3H, s, OCH_3), 3.59 (1H, dd, J = 10.6, 6.4 Hz, *H*-6), 3.31 (1H, dd, J = 10.6, 9.4 Hz, *H*-5), 2.42 (3H, s, CH_3), 1.34 (3H, s, CH_3), 1.32 (3H, s, CH_3), 1.28 (3H, s, CH_3), 1.18 (3H, s, CH_3). ^{13}C NMR (100 MHz, $CDCl_3$): δ = 159.2 (C), 144.8 (C), 133.9 (C), 130.0 (C), 129.6, 129.5 (CH), 128.2 (CH), 113.6 (CH), 112.5 (C), 110.3 (C), 81.3 (CH), 79.0 (CH), 78.5 (CH), 77.2 (CH), 75.9 (CH), 74.2 (CH), 71.7 (OCH_2), 55.3 (OCH_3), 30.9 (CH_3), 27.7 (CH_3), 26.8 (CH_3), 26.7 (CH_3), 25.7 (CH_3). HRMS (ES): m/z found 557.1796, $C_{27}H_{34}NaO_9S^+$ $[M+Na]^+$ requires 557.1816. Anal. Calc. for $C_{27}H_{34}O_9S$: C, 60.66; H, 6.41. Found: C, 60.6; H, 6.46.

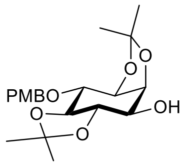

28: D/L-1,2:4,5-Di-*O*-isopropylidene-6-*O*-*p*-methoxybenzyl-*myo*-inositol. Compound **27** (7.60 g, 14.22 mmol) and magnesium powder (5.12 g, 213.3 mmol) in MeOH (150 mL) and CH_2Cl_2 (150 mL) was stirred at rt for 1.5 h in a 1 litre flask with a reflux condenser. After 15-20 min the solvent temperature increased to reflux. After the reaction had ceased, the solvents were evaporated to dryness to give a greyish solid (~40 g), which was split into two lots with each lot suspended in CH_2Cl_2 (200 mL) and 0.2 M HCl (100 mL) was added which gave a light coloured precipitate. The mixture was then filtered over a bed of celite and washed with CH_2Cl_2. The organic solvent was separated from the aqueous layer and the solvent was evaporated to give a white solid. The remaining celite/magnesium salt mixture was air dried overnight and further washed with more CH_2Cl_2 and dried ($MgSO_4$). The mixture was then purified by flash column chromatography (CH_2Cl_2 to CH_2Cl_2/EtOAc 1:1) to afford compound **28** (4.935 g, 91%; R_f = 0.36 in Et_2O). ^1H NMR (400 MHz, $CDCl_3$): δ = 7.32 (2H, d, J = 8.8 Hz, 2 x CH), 6.86 (2H, d, J = 8.7 Hz, 2 x CH), 4.73 (2H, s, OCH_2), 4.44 (1H, t, J = 4.9 Hz, *H*-2), 4.17 (1H, t, J = 6.0 Hz, *H*-1), 3.97 (1H, ddd, J = 10.2, 8.8, 4.6 Hz, *H*-3), 3.79 (3H, s, OCH_3), 3.78 (1H, t, J = 9.6 Hz, *H*-4), 3.65 (1H, dd, J = 10.6, 6.2 Hz, *H*-6), 3.38 (1H, dd,

J = 10.5, 9.5 Hz, *H*-5), 2.37 (1H, d, *J* = 8.8 Hz, OH), 1.47 (3H, s, CH_3), 1.44 (3H, s, CH_3), 1.39 (3H, s, CH_3), 1.35 (3H, s, CH_3). ^{13}C NMR (100 MHz, $CDCl_3$): δ = 159.2 (C), 130.2 (C), 129.6 (CH), 113.6 (CH), 112.3 (C), 109.9 (C), 81.4 (CH), 80.0 (CH), 78.5 (CH), 77.9 (CH), 77.6 (CH), 71.6 (OCH_2), 69.9 (CH), 55.3 (OCH_3), 27.8 (CH_3), 27.0 (CH_3), 27.0 (CH_3), 25.9 (CH_3). HRMS (ES): m/z found 403.1714, $C_{20}H_{28}NaO_7^+$ [M+Na]$^+$ requires 403.1727. Anal. Calc. for $C_{20}H_{28}O_7$: C, 63.14; H, 7.42. Found: C, 63.1; H, 7.48.

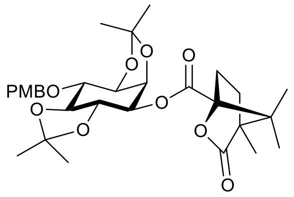

29: L-3-*O*-(1S)-Camphanoyl-6-*O*-*p*-methoxybenzyl-1,2:4,5-di-*O*-isopropylidene-*myo*-inositol. Compound **28** (9.89 g, 26.0 mmol) was dissolved in CH_2Cl_2 (100 mL) and DMAP (122 mg, 1.0 mmol), pyridine (10 mL) and (1S)-(-)-camphanic chloride (7.00 g, 32.3 mmol) and the reaction was stirred at rt for 18 h. The solvents were evaporated and the resulting residue was dissolved in CH_2Cl_2 (200 mL) and washed with water (200 mL). The organic solution was dried and the solvents were evaporated. The remaining white solid was purified by flash column chromatography (CH_2Cl_2 to CH_2Cl_2/EtOAc 4:1) to afford compound **29** as a white solid (4.53 g, 31%; R_f = 0.18 in CH_2Cl_2/EtOAc 10:1). $[\alpha]_D^{22}$ = −5.5° (c = 1.0, $CHCl_3$). ^{1}H NMR (400 MHz, $CDCl_3$): 7.31 (2H, d, *J* = 8.7 Hz, 2 x CH), 6.86 (2H, d, *J* = 8.7 Hz, 2 x CH), 5.14 (1H, dd, *J* = 10.5, 4.4 Hz, *H*-3), 4.74 (2H, s, OCH_2), 4.65 (1H, t, *J* = 4.8 Hz, *H*-2), 4.19 (1H, dd, *J* = 6.2, 5.3 Hz, *H*-1), 3.97 (1H, dd, *J* = 10.4, 9.6 Hz, *H*-4), 3.79 (3H, s, OCH_3), 3.66 (1H, dd, *J* = 10.6, 6.4 Hz, *H*-6), 3.47 (1H, dd, *J* = 10.5, 9.5 Hz, *H*-5), 2.52-2.44 (1H, m, C*H* of CH_2), 2.12-2.05 (1H, m, C*H* of CH_2), 1.96-1.89 (1H, m, C*H* of CH_2), 1.72-1.66 (1H, m, C*H* of CH_2), 1.45 (3H, s, CH_3), 1.42 (3H, s, CH_3), 1.35 (3H, s, CH_3), 1.27 (3H, s, CH_3), 1.10 (3H, s, CH_3), 1.09 (3H, s, CH_3), 0.95 (3H, s, CH_3). ^{13}C NMR (100 MHz, $CDCl_3$): δ = 177.9 (C), 166.8 (C), 159.3 (C), 130.1 (C), 129.7 (CH), 113.7 (CH), 112.6 (C), 109.9 (C), 91.1, (C), 81.3 (CH), 79.3 (CH), 78.6 (CH), 74.4 (CH), 74.4 (CH), 72.3 (CH), 71.7 (OCH_2), 55.3 (OCH_3), 54.8 (C), 54.5 (C), 30.4 (CH_2), 29.1 (CH_2), 27.7 (CH_3), 27.0 (CH_3), 26.8 (CH_3), 25.7 (CH_3), 16.4 (CH_3), 16.3 (CH_3), 9.7 (CH_3). HRMS (ES): m/z found 583.2506,

$C_{30}H_{40}NaO_{10}^{+}$ [M+Na]$^{+}$ requires 583.2514. Anal. Calc. for $C_{30}H_{40}O_{10}$: C, 64.27; H, 7.19. Found: C, 64.2; H, 7.26.

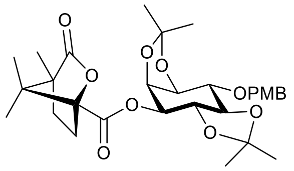

30: D-3-*O*-(1S)-Camphanoyl-6-*O*-*p*-methoxybenzyl-1,2:4,5-di-*O*-isopropylidene *myo*-inositol. Additionally, compound **30** was isolated as a white solid (6.12 g, 42%; R_f = 0.32 in CH_2Cl_2/EtOAc 10:1). $[\alpha]_D^{22}$ = −9.9° (c = 1.0, $CHCl_3$). ^{1}H NMR (400 MHz, $CDCl_3$): δ = 7.31 (2H, d, *J* = 8.7 Hz, 2 x CH), 6.86 (2H, d, *J* = 8.7 Hz, 2 x CH), 5.15 (1H, dd, *J* = 10.5, 4.4 Hz, *H*-3), 4.74 (2H, s, OCH_2), 4.60 (1H, t, *J* = 4.8 Hz, *H*-2), 4.18 (1H, dd, *J* = 6.2, 5.3 Hz, *H*-1), 4.01 (1 H, dd, *J* = 10.4, 9.6 Hz, *H*-4), 3.79 (3H, s, OCH_3), 3.66 (1H, dd, *J* = 10.6, 6.3 Hz, *H*-6), 3.46 (1H, dd, *J* = 10.5, 9.5 Hz, *H*-5), 2.50-2.43 (1H, m, C*H* of CH_2), 2.10-2.03 (1H, m, C*H* of CH_2), 1.95-1.87 (1H, m, C*H* of CH_2), 1.71-1.64 (1H, m, C*H* of CH_2), 1.45 (3H, s, CH_3), 1.42 (3H, s, CH_3), 1.34 (3H, s, CH_3), 1.26 (3H, s, CH_3), 1.11 (3H, s, CH_3), 1.04 (3H, s, CH_3), 0.98 (3H, s, CH_3). ^{13}C NMR (100 MHz, $CDCl_3$): δ = 178.1 (C), 167.1 (C), 159.3 (C), 130.2 (C), 129.7 (CH), 113.7 (CH), 112.6 (C), 110.0 (C), 91.0, (C), 81.4 (CH), 79.3 (CH), 78.6 (CH), 74.6 (CH), 74.4 (CH), 72.2 (CH), 71.8 (OCH_2), 55.3 (OCH_3), 54.9 (C), 54.7 (C), 30.6 (CH_2), 29.0 (CH_2), 27.8 (CH_3), 27.0 (CH_3), 26.9 (CH_3), 25.8 (CH_3), 16.6 (CH_3), 16.5 (CH_3), 9.7 (CH_3). HRMS (ES): m/z found 583.2527, $C_{30}H_{40}NaO_{10}^{+}$ [M+Na]$^{+}$ requires 583.2514. Anal. Calc. for $C_{30}H_{40}O_{10}$: C, 64.27; H, 7.19. Found: C, 64.2; H, 7.23.

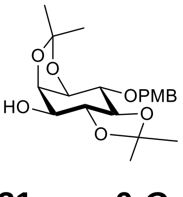

31: D-6-*O*-*p*-Methoxybenzyl-1,2:4,5-di-*O*-isopropylidene *myo*-inositol. Compound **30** (560 mg, 1.0 mmol) was dissolved in MeOH (20 mL) and heated to 80 °C. Sodium hydroxide (0.40 g, 10 mmol) was then added as a solid and the reaction mixture stirred for 30 minutes at reflux. After cooling to rt the solvent was evaporated and the residue was partitioned between CH_2Cl_2 (50 mL) and

water (40 mL) with added brine (10 mL). The layers were separated and the aqueous layer was extracted with CH_2Cl_2 (2 x 50 mL). The combined organic layers were dried by filtration through NaCl and concentrated in vacuum to give compound **31** as a white solid (332 mg, 87%). M.p. 112-114 °C. $[\alpha]_D^{22}$ = –56.6° (c = 0.8; acetone). ^{1}H NMR (400 MHz, acetone-d$_6$): δ = 7.31 (2H, d, *J* = 8.8 Hz, 2 x CH), 6.89 (2H, d, *J* = 8.8 Hz, 2 x CH), 4.70 (2H, s, OCH_2), 4.42 (1H, t, *J* = 4.7 Hz), 4.14 (1H, dd, *J* = 6.4, 5.2 Hz), 4.09 (1H, D_2O exch, d, *J* = 7.6 Hz, OH), 4.07-3.99 (1H, m), 3.81 (1H, t, *J* = 9.7 Hz), 3.78 (3H, s, CH_3), 3.65 (1H, dd, *J* = 10.6, 6.5 Hz), 3.43 (1H, dd, *J* = 10.6, 9.3 Hz), 1.39 (3H, s, CH_3), 1.37 (3H, s, CH_3), 1.35 (3H, s, CH_3), 1.29 (3H, s, CH_3). ^{13}C NMR (100 MHz, acetone-d$_6$): δ = 160.1 (C), 131.7 (C), 130.1 (CH), 114.3 (CH), 111.8 (C), 109.7 (C), 82.0 (CH), 81.4 (CH), 79.7 (CH), 79.6 (CH), 78.9 (CH), 72.1 (OCH_2), 70.0 (CH), 55.5 (OCH_3), 28.3 (CH_3), 27.3 (CH_3), 27.3 (CH_3), 26.1 (CH_3). HRMS (ES): m/z found 403.1721, $C_{20}H_{28}NaO_7^+$ [M+Na]$^+$ requires 403.1721.

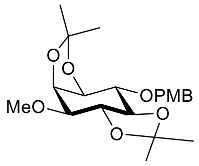

32: **D-6-*O*-*p*-Methoxybenzyl-3-*O*-methyl-1,2:4,5-di-*O*-isopropylidene *myo*-inositol.** Compound **31** (190 mg, 0.5 mmol) was dissolved in DMF (2 mL) and sodium hydride (19 mg, 0.75 mmol, 1.5 equiv) was added. The reaction mixture was stirred for 30 min at rt. Iodomethane (85 mg, 0.6 mmol, 1.2 equiv.) was added and the reaction mixture was stirred for 18 at rt. The reaction mixture was then diluted with water (100 mL) and extracted with EtOAc (2 x 100 mL). The combined organic layers were dried by filtration through NaCl and concentrated in vacuum. Purification by flash column chromatography (CH_2Cl_2 and 1% Et_3N to CH_2Cl_2/EtOAc 9:1 and 1% Et_3N) afforded compound **32** as white solid (129 mg, 65%). M.p. 92-94 °C. $[\alpha]_D^{22}$ = –44.7° (c = 0.4; acetone). ^{1}H NMR (400 MHz, acetone-d$_6$): δ = 7.31 (2H, d, *J* = 8.8 Hz, 2 x CH), 6.90 (2H, d, *J* = 8.8 Hz, 2 x CH), 4.70 (2H, s, OCH_2), 4.58 (1H, t, *J* = 4.6 Hz), 4.14 (1H, dd, *J* = 6.5, 5.0 Hz), 3.86 (1H, dd, *J* = 10.1, 9.4 Hz), 3.78 (3H, s, OCH_3), 3.74 (1H, dd, *J* = 10.1, 4.2 Hz), 3.66 (1H, dd, *J* = 10.6, 6.6 Hz), 3.46 (1H, dd, *J* = 10.5, 9.3 Hz), 3.44 (3H, s, OCH_3), 1.39 (3H, s, CH_3), 1.38 (3H, s, CH_3), 1.34 (3H, s, CH_3),

1.29 (3H, s, CH_3). ^{13}C NMR (100 MHz, acetone-d_6): δ = 160.1 (C), 131.7 (C), 130.1 (CH), 114.3 (CH), 112.1 (C), 109.9 (C), 82.0 (CH), 81.2 (CH), 79.8 (CH), 78.3 (CH), 78.0 (CH), 76.6 (CH), 72.2 (OCH_2), 57.3 (OCH_3), 55.5 (OCH_3), 28.3 (CH_3), 27.3 (CH_3), 27.3 (CH_3), 26.2 (CH_3). HRMS (ES): m/z found 417.1878, $C_{21}H_{30}NaO_7^+$ $[M+Na]^+$ requires 417.1884.

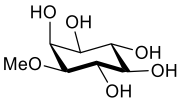

33: L-1-*O*-Methyl-*myo*-inositol (L-(+)-bornesitol). Compound **32** (99 mg, 0.25 mmol) was treated with anhydrous TFA/CH_2Cl_2 (1:9; 2 mL) for 15 min at rt. The reaction mixture was then evaporated and the residue was then treated with TFA/water (9:1; 2 mL) for another 15 min at rt. The reaction mixture was again evaporated. The residue was treated with hot CH_2Cl_2/MeOH (~5 mL; ~4:1) to give compound **33** as a white solid (39 mg, 80%). M.p. 203-205 °C, Lit.[10] 205–207 °C, Lit.[11] 204–206 °C. $[\alpha]_D^{26}$ = +32.0° (c = 0.7; H_2O), Lit.[10] $[\alpha]_D^{20}$ = +31.9° (*c* = 1, H_2O), Lit.[11] $[\alpha]_D^{20}$ = +31° (*c* = 0.7, H_2O). ^{1}H NMR (400 MHz, D_2O): δ = 4.27 (1H, t, *J* = 2.8 Hz), 3.61 (1H, t, *J* = 9.9 Hz), 3.58 (1H, t, *J* = 9.9 Hz), 3.46 (1H, dd, *J* = 10.0, 2.9 Hz), 3.40 (3H, s, OCH_3), 3.24 (1H, t, *J* = 9.4 Hz), 3.17 (1H, dd, *J* = 10.0, 2.8 Hz). ^{13}C NMR (100 MHz, D_2O): δ = 80.5 (CH), 74.4 (CH), 72.2 (CH), 71.6 (CH), 71.0 (CH), 67.6 (CH), 56.7 (OCH_3).

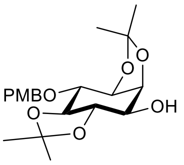

34: L-6-*O*-*p*-Methoxybenzyl-1,2:4,5-di-*O*-isopropylidene *myo*-inositol. Compound **29** (2.24 g, 4.0 mmol) was dissolved in MeOH (60 mL) and heated to 80 °C (drysyn temperature). Sodium hydroxide (0.96 g, 40 mmol) was then added as a solid and the reaction mixture stirred for 30 min at reflux. After cooling to rt the solvent was evaporated and the residue was partitioned between CH_2Cl_2 (200 mL) and water (80 mL) with added brine (20 mL). The layers were separated and the aqueous layer was extracted with CH_2Cl_2 (2 x 100 mL). The combined organic layers were dried by filtration through solid NaCl and concentrated in vacuum to give compound **34** as a white solid (1.39

g, 91%). M.p. 112-114 °C. $[\alpha]_D^{22}$ = +56.7° (c = 0.8; acetone). ^{1}H NMR (400 MHz, acetone-d_6): δ = 7.31 (2H, d, *J* = 8.8 Hz, 2 x CH), 6.89 (2H, d, *J* = 8.8 Hz, 2 x CH), 4.70 (2H, s, OCH_2), 4.42 (1H, t, *J* = 4.7 Hz), 4.14 (1H, dd, *J* = 6.4, 5.2 Hz), 4.11 (1H, D_2O exch, d, *J* = 7.6 Hz, OH), 4.07-3.99 (1H, m), 3.81 (1H, t, *J* = 9.6 Hz), 3.78 (3H, s, OCH_3), 3.65 (1H, dd, *J* = 10.6, 6.5 Hz), 3.43 (1H, dd, *J* = 10.6, 9.3 Hz), 1.39 (3H, s, CH_3), 1.37 (3H, s, CH_3), 1.35 (3H, s, CH_3), 1.29 (3H, s, CH_3). ^{13}C NMR (100 MHz, acetone-d_6): δ = 160.1 (C), 131.7 (C), 130.1 (CH), 114.3 (CH), 111.8 (C), 109.7 (C), 82.0 (CH), 81.4 (CH), 79.7 (CH), 79.6 (CH), 78.9 (CH), 72.1 (OCH_2), 70.0 (CH), 55.5 (OCH_3), 28.3 (CH_3), 27.3 (CH_3), 27.3 (CH_3), 26.1 (CH_3). HRMS (ES): m/z found 403.1719, $C_{20}H_{28}NaO_7^+$ [M+Na]$^+$ requires 403.1727.

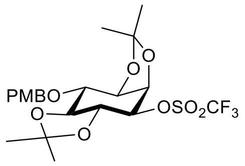

16: L-3-*O*-Trifluoromethylsulfonyl-6-*O*-*p*-methoxybenzyl-1,2:4,5-di-*O*-isopropylidene *myo*-inositol. Compound **34** (1.37 g, 3.6 mmol) was dissolved in CH_2Cl_2 (18 mL) and pyridine (2 mL) and cooled to 0 °C. Trifluoromethane sulfonic anhydride (4.3 mL, 4.3 mmol, 1M in CH_2Cl_2) was added dropwise via a dropping funnel. The reaction mixture stirred for 30 min at 0 °C, then for 2 h warming up to rt. The mixture was diluted with CH_2Cl_2 (80 mL) and washed with water (80 mL) and added brine (20 mL). The organic layer was dried by filtration through solid NaCl and concentrated in vacuum to give compound **16** as a pale beige amorphous solid (1.78 g, 97%). $[\alpha]_D^{22}$ = +23.4° (c = 0.5; acetone). ^{1}H NMR (400 MHz, acetone-d_6): δ = 7.31 (2H, d, *J* = 8.7 Hz, 2 x CH), 6.91 (2H, d, *J* = 8.7 Hz, 2 x CH), 5.54 (1H, dd, *J* = 10.4, 4.6 Hz), 4.71 (2H, s, OCH_2), 4.69 (1H, t, *J* = 4.8 Hz) 4.40-4.35 (1H, m), 4.18-4.12 (1H, m), 3.82-3.78 (2H, m), 3.79 (3H, s, OCH_3), 1.46 (3H, s, CH_3), 1.43 (3H, s, CH_3), 1.42 (3H, s, CH_3), 1.34 (3H, s, CH_3). ^{13}C NMR (100 MHz, acetone-d_6): δ = 160.2 (C), 131.3 (C), 130.2 (CH), 114.4 (CH), 113.6 (C), 111.3 (C), 85.5 (CH), 82.7 (CH), 80.5 (CH), 78.8 (CH), 76.6 (CH), 75.2 (CH), 72.3 (OCH_2), 55.5 (OCH_3), 27.9 (CH_3), 27.2 (CH_3), 27.0 (CH_3), 26.0 (CH_3). HRMS (ES): m/z 513.1387, $C_{21}H_{28}F_3O_9S^+$ [M+H]$^+$ requires 513.1401.

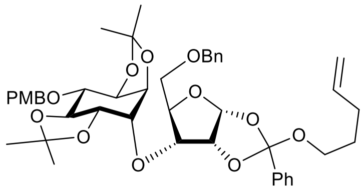

35: 3-*O*-(2′,3′:5′,6′-*O*-Diisopropylidene-4′-*O*-*p*-methoxybenzyl-1′-D-*chiro*-inositol)-5-*O*-benzyl-*β*-D-ribofuranose-1,2-(pent-4-enyl orthobenzoate). Compound **15** (680 mg, 1.65 mmol) was dissolved in THF (0.8 mL). HMPA (2.4 mL) and then NaH (95%, 125 mg, 5.0 mmol) were added and the reaction mixture was stirred until its colour had turned dark-grey (~30 min). Compound **16** (1.23 g, 2.4 mmol) was added as a solid and the reaction mixture was stirred at rt for 18 h. The THF was then evaporated and the mixture was diluted with diethyl ether (100 mL) and washed with water (100 mL). The organic layer was dried by filtration through solid NaCl and concentrated in vacuum. Purification by flash column chromatography (PE/EtOAc 9:1 and 1% Et_3N to PE/EtOAc 3:2 and 1% Et_3N) afforded compound **35** as a colourless viscous oil (1.13 g, 86%). $[\alpha]_D^{22}$ = +91.2° (c = 0.4; acetone). ^{1}H NMR (400 MHz, acetone-d_6): δ = 7.73-7.67 (2H, m, 2 x CH), 7.40-7.36 (3H, m, 3 x CH), 7.34-7.30 (6H, m, 6 x CH), 7.29-7.23 (1H, m, CH), 6.89 (2H, d, *J* = 8.7 Hz, 2 x CH), 6.06 (1H, d, *J* = 4.1 Hz), 5.88-5.75 (1H, m), 5.06 (1H, dd, *J* = 4.8, 4.3 Hz), 5.01 (1H, ddt, *J* = 17.2, 2.1, 1.6 Hz), 4.93 (1H, ddt, *J* = 10.2, 2.1, 1.2 Hz), 4.71 (2H, s, OCH_2), 4.55 (1H, d, *J* = 12.1 Hz), 4.48 (1H, d, *J* = 12.1 Hz), 4.34 (1H, dd, *J* = 2.4, 2.3 Hz), 4.31 (1H, dd, *J* = 5.5, 2.1 Hz), 4.14 (1H, t, *J* = 5.9 Hz), 4.09 (1H, t, *J* = 10.0 Hz), 4.04-4.00 (1H, m), 3.79-3.75 (1H, m), 3.78 (3H, s, OCH_3), 3.68-3.56 (4H, m), 3.49-3.43 (2H, m), 2.14-2.07 (2H, m), 1.67-1.59 (2H, m), 1.57 (3H, s, CH_3), 1.44 (3H, s, CH_3), 1.35 (3H, s, CH_3), 1.26 (3H, s, CH_3). ^{13}C NMR (100 MHz, acetone-d_6): δ = 160.1 (C), 139.7 (C), 139.3 (C), 139.1 (CH), 131.7 (C), 130.1 (CH), 129.6 (CH), 129.1 (CH), 128.6 (CH), 128.5 (CH), 128.3 (CH), 127.1 (CH), 124.6 (C), 115.2 (CH_2), 114.3 (CH), 111.9 (C), 109.9 (C), 105.0 (CH), 83.0 (CH), 82.1 (CH), 81.4 (CH), 80.0 (CH), 79.1 (CH), 78.9 (CH), 77.5 (CH), 76.4 (CH), 75.9 (CH), 73.7 (OCH_2), 72.1 (OCH_2), 69.1 (OCH_2), 62.3 (OCH_2), 55.5 (OCH_3), 31.0 (CH_2), 29.5 (CH_2), 28.0 (CH_3), 27.5 (CH_3), 27.2 (CH_3), 26.1 (CH_3). HRMS (ES): m/z found 775.3698, $C_{44}H_{55}O_{12}^+$ $[M+H]^+$ requires 775.3688.

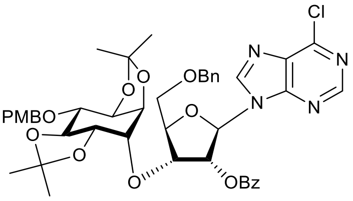

14: 6-Chloro-9-[2′-*O*-benzoyl-5′-*O*-benzyl-3′-*O*-(2″,3″:5″,6″-*O*-diisopropylidene-4″-*O*-*p*-methoxybenzyl-1″-D-*chiro*-inosityl)-*β*-D-ribofuranosyl]purine. Compound **35** (1.01 g, 1.3 mmol) was dissolved in MeCN (1.3 mL) added to freshly prepared trimethylsilylated 6-chloropurine (325 mg, 1.43 mmol) in MeCN (2.6 mL). Activated MS 3A (powder, 200 mg) was added and the mixture stirred at rt for 10 min. In the meantime, *N*-iodosuccinimide (322 mg, 1.43 mmol) and $Yb(OTf)_3$ (242 mg, 0.39) were separately dispensed in MeCN (2.6 mL) and stirred over activated MS 3A (powder, 130 mg) at rt for 10 min. The two mixtures were then combined via glass pipette at 0 °C and stirred at rt for 16 h. The reaction mixture was quenched with $Na_2S_2O_3$ (sat., 40 mL) and extracted with CH_2Cl_2 (3 x 80 mL). The combined organic layers were dried by filtration through solid NaCl and concentrated in vacuum. Purification by flash column chromatography (PE/EtOAc 9:1 and 1% Et_3N to PE/EtOAc 3:2 and 1% Et_3N) afforded compound **14** as a colourless glass (758 mg, 69%). $[\alpha]_D^{22}$ = +12.9° (c = 0.4; acetone). ^{1}H NMR (400 MHz, acetone-d$_6$): δ = 8.76 (1H, s, CH), 8.69 (1H, s, CH), 8.14-8.09 (2H, m, 2 x CH), 7.64 (1H, ddt, *J* = 7.9, 7.0, 1.3, CH), 7.53-7.47 (2H, m, 2 x CH), 7.45-7.41 (2H, m, 2 x CH), 7.40-7.35 (2H, m, 2 x CH), 7.32-7.30 1H, m, CH), 7.28 (2H, d, *J* = 8.7 Hz, 2 x CH), 6.89 (2H, d, *J* = 8.7 Hz, 2 x CH), 6.61 (1H, d, *J* = 4.1 Hz), 6.29 (1H, dd, *J* = 5.0, 4.1 Hz), 5.21 (1H, t, *J* = 5.3 Hz), 4.76 (1H, d, *J* = 11.9 Hz), 4.69 (1H, d, *J* = 11.9 Hz), 4.64 (2H, s, OCH_2), 4.59 (1H, dt, *J* = 5.4, 3.1 Hz), 4.49-4.44 (2H, m), 4.13 (1H, dd, *J* = 6.0, 5.4 Hz), 4.01 (1H, dd, *J* = 11.1, 3.1 Hz), 3.95 (1H, t, *J* = 10.0 Hz), 3.92 (1H, dd, *J* = 11.1, 3.1 Hz), 3.78 (3H, s, OCH_3), 3.70 (1H, dd, *J* = 10.0, 2.6 Hz), 3.60 (1H, dd, *J* = 10.0, 6.1 Hz), 1.33 (3H, s, CH_3), 1.26 (3H, s, CH_3), 1.25 (3H, s, CH_3), 1.01 (3H, s, CH_3). ^{13}C NMR (100 MHz, acetone-d$_6$): δ = 165.8 (C), 160.1 (C), 152.6 (CH), 145.6 (CH), 139.0 (C), 136.9 (C), 134.2 (CH), 132.9 (C), 131.7 (C), 130.9 (CH), 130.6 (C), 130.0 (CH), 129.3 (CH), 128.6 (CH), 128.5 (CH), 114.3 (CH), 111.6 (C), 110.0 (C), 88.1 (CH), 83.3 (CH), 82.9 (CH), 81.4 (CH), 78.1 (CH), 77.1 (CH), 76.1 (CH), 75.5 (CH), 74.1 (OCH_2), 73.0 (CH), 72.0 (OCH_2), 69.7 (OCH_2), 55.5

(OCH$_3$), 28.0 (CH$_3$), 27.3 (CH$_3$), 26.5 (CH$_3$), 26.1 (CH$_3$); one aliphatic and one aromatic CH hidden. HRMS (ES): m/z found 843.3021, $C_{44}H_{48}ClN_4O_{11}^+$ $[M+H]^+$ requires 843.3003.

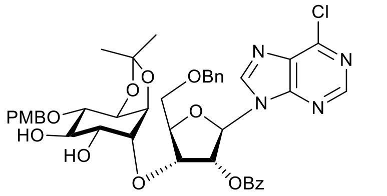

13: 6-Chloro-9-[2′-*O*-benzoyl-5′-*O*-benzyl-3′-*O*-(5″,6″-*O*-isopropylidene-4″-*O*-*p*-methoxybenzyl-1″-D-*chiro*-inosityl)-*β*-D-ribofuranosyl]purine.

Compound **14** (717 mg, 0.85 mmol) was dissolved in CH_2Cl_2 (34 mL) and stirred vigorously. Ethylene glycol (105 mg, 1.7 mmol) and *p*-TSA (32 mg, 0.17 mmol) were added. The reaction mixture was then stirred for 20 min at rt, diluted with CH_2Cl_2 (100 mL) and immediately washed with ammonia (2% in water, 100 mL). The organic layer was dried by filtration through solid NaCl and concentrated in vacuum to give compound **13** was a white foam (672 mg, 98%). $[\alpha]_D^{22}$ = +4.9° (c = 0.8; acetone). ^{1}H NMR (400 MHz, acetone-d$_6$): δ = 8.74 (1H, s, CH), 8.71 (1H, s, CH), 8.10-8.05 (2H, m, 2 x CH), 7.64 (1H, ddt, *J* = 7.9, 7.0, 1.3 Hz, CH), 7.52-7.46 (2H, m, 2 x CH), 7.44-7.39 (2H, m, 2 x CH), 7.39-7.33 (2H, m, 2 x CH), 7.33-7.27 3H, m, 3 x CH), 6.87 (2H, d, *J* = 8.7 Hz, 2 x CH), 6.60 (1H, d, *J* = 4.5 Hz), 6.15 (1H, t, *J* = 4.8 Hz), 5.16 (1H, t, *J* = 5.0 Hz), 4.76 (1H, d, *J* = 11.5 Hz), 4.72 (1H, d, *J* = 12.0 Hz), 4.68 (1H, d, *J* = 12.0 Hz), 4.64 (1H, d, *J* = 11.5 Hz), 4.57 (1H, dt, *J* = 4.9, 3.1 Hz), 4.44 (1H, dd, *J* = 6.6, 5.3 Hz), 4.25 (1H, dd, *J* = 8.1, 6.7 Hz), 4.09 (1H, D_2O exch, *J* = 4.1 Hz), 4.07 (1H, dd, *J* = 5.3, 2.5 Hz), 3.99 (1H, dd, *J* = 10.9, 2.9 Hz), 3.92 (1H, dd, *J* = 11.0, 3.4 Hz), 3.91 (1H, D_2O exch, d, *J* = 4.1 Hz), 3.82-3.77 (1H, m), 3.78 (3H, s, OCH$_3$), 3.74-3.68 (1H, m), 3.39 (1H, t, *J* = 8.1 Hz), 1.40 (3H, s, CH$_3$), 1.29 (3H, s, CH$_3$). ^{13}C NMR (100 MHz, acetone-d$_6$): δ = 166.3 (C), 160.0 (C), 152.7 (C), 152.6 (CH), 150.9 (C), 145.6 (CH), 139.0 (C), 134.3 (CH), 132.8 (C), 132.1 (C), 130.7 (CH), 130.3 (C), 130.0 (CH), 129.4 (CH), 129.2 (CH), 128.5 (CH), 114.2 (CH), 109.8 (C), 87.7 (CH), 84.2 (CH), 84.1 (CH), 79.8 (CH), 79.6 (CH), 78.3 (CH), 77.4 (CH), 76.5 (CH), 74.7 (CH), 74.2 (CH), 74.0 (OCH$_2$), 73.2 (OCH$_2$), 70.0 (OCH$_2$), 55.5 (OCH$_3$), 28.1 (CH$_3$), 25.8 (CH$_3$); one aromatic CH hidden. HRMS (ES): m/z found 803.2716, $C_{41}H_{44}ClN_4O_{11}^+$ $[M+H]^+$ requires 803.2690.

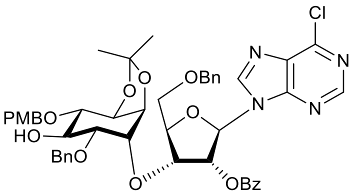

36: 6-Chloro-9-[2′-*O*-benzoyl-5′-*O*-benzyl-3′-*O*-(2″-*O*-benzyl-5″,6″-*O*-isopropylidene-4″-*O*-*p*-methoxybenzyl-1″-D-*chiro*-inosityl)-*β*-D-ribofuranosyl]purine. Compound **13** (200 mg, 0.25 mmol) was dissolved in acetonitrile (50 mL), di-n-butyltinoxide (125 mg, 0.5 mmol) was added and the reaction mixture was heated at reflux for 18 h (140 °C drysyn temperature) using a Soxhlet extractor with activated MS 3A. The reaction mixture was cooled to rt, the solvent removed in vacuum and the residue was dried in HV for 22 h. The resulting white solid was dissolved in DMF (2.0 mL) and powder of freshly activated MS 3A (100 mg) was added. Cesium fluoride (153 mg, 1.0 mmol), benzyl bromide (128 mg, 0.75 mmol) and tetra-*n*-butylammonium iodide (19 mg, 0.05 mmol) were added and the reaction mixture was stirred at rt for 72 h. The reaction mixture was then concentrated in vacuum and the residue partitioned between CH_2Cl_2 (100 mL) and water (100 mL). The aqueous layer was further extracted with CH_2Cl_2 (2 x 100 mL) and the combined organic layers were then dried by filtration through solid NaCl and concentrated in vacuum. Purification by flash column chromatography (PE/EtOAc 9:1 and 1% Et_3N to PE/EtOAc 1:4 and 1% Et_3N) afforded compound **36** as a colourless glass (58 mg, 26%). $[\alpha]_D^{22}$ = –3.1° (c = 1.0; acetone). ^{1}H NMR (400 MHz, acetone-d$_6$): δ = 8.74 (1H, s, CH), 8.71 (1H, s, CH), 8.04-7.99 (2H, m, 2 x CH), 7.61 (1H, ddt, *J* = 7.9, 7.0, 1.3 Hz, CH), 7.46-7.17 (14H, m, 14 x CH), 6.87 (2H, d, *J* = 8.7 Hz, 2 x CH), 6.62 (1H, d, *J* = 4.8 Hz), 6.10 (1H, t, *J* = 5.0 Hz), 5.10 (1H, t, *J* = 4.9 Hz), 4.76 (1H, d, *J* = 11.5 Hz), 4.71 (1H, d, *J* = 12.0 Hz), 4.67 (1H, d, *J* = 12.0 Hz), 4.65 (1H, d, *J* = 11.5 Hz), 4.65-4.61 (1H, m), 4.55-4.49 (3H, m), 4.30 (1H, D_2O exch, d, *J* = 4.1 Hz, OH), 4.28 (1H, dd, *J* = 8.7, 7.1 Hz), 4.06 (1H, dd, *J* = 6.4, 2.1 Hz), 3.99 (1H, dd, *J* = 10.9, 2.9 Hz), 3.90 (1H, dd, *J* = 10.9, 3.4 Hz), 3.86 (1H, dd, *J* = 7.0, 3.4 Hz), 3.77 (3H, s, OCH_3), 3.63 (1H, dd, *J* = 3.4, 2.2 Hz), 3.42 (1H, dd, *J* = 8.8, 7.0 Hz), 1.41 (3H, s, CH_3), 1.30 (3H, s, CH_3). ^{13}C NMR (100 MHz, acetone-d$_6$): δ = 166.0 (C), 160.0 (C), 152.7 (C), 152.6 (CH), 151.0 (C), 145.6 (CH), 139.6 (C), 139.0 (C), 134.3 (CH), 132.8 (C), 132.1 (C),

130.6 (CH), 130.0 (CH), 129.4 (CH), 129.2 (CH), 128.9 (CH), 128.5 (CH), 128.0 (CH), 114.2 (CH), 110.0 (C), 87.8 (CH), 84.4 (CH), 84.4 (CH), 83.1 (CH), 79.8 (CH), 79.1 (CH), 78.8 (CH), 78.1 (CH), 76.5 (CH), 74.4 (CH), 74.0 (OCH_2), 73.2 (OCH_2), 73.2 (OCH_2), 70.2 (OCH_2), 55.5 (OCH_3), 28.2 (CH_3), 25.6 (CH_3); two aromatic CH hidden. HRMS (ES): m/z found 893.3175, $C_{48}H_{50}ClN_4O_{11}^+$ (M+H)$^+$ requires 893.3159.

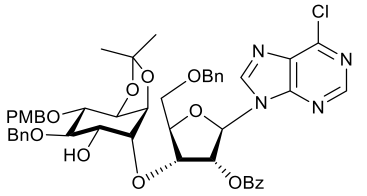

37: 6-Chloro-9-[2′-*O*-benzoyl-5′-*O*-benzyl-3′-*O*-(3″-*O*-benzyl-5″,6″-*O*-isopropylidene-4″-*O*-*p*-methoxybenzyl-1″-D-*chiro*-inosityl)-*β*-D-ribofuranosyl]purine. Additionally, compound **37** was isolated as a colourless glass (62 mg; 28%). $[\alpha]_D^{22}$ = +4.7° (c = 0.45; acetone). ^{1}H NMR (400 MHz, acetone-d$_6$): δ = 8.75 (1H, s, CH), 8.71 (1H, s, CH), 8.11-8.06 (2H, m, 2 x CH), 7.61 (1H, ddt, *J* = 7.9, 7.0, 1.3, CH), 7.49-7.44 (2H, m, CH), 7.42-7.22 (12H, m, 12 x CH), 6.84 (2H, d, *J* = 8.7 Hz, 2 x CH), 6.62 (1H, d, *J* = 4.3 Hz), 6.10 (1H, t, *J* = 4.7 Hz), 5.18 (1H, t, *J* = 5.2 Hz), 4.73 (1H, d, *J* = 11.4 Hz), 4.72 (1H, d, *J* = 12.0 Hz), 4.67 (1H, d, *J* = 12.0 Hz), 4.61 (1H, d, *J* = 11.4 Hz), 4.57 (1H, dt, *J* = 5.2, 3.0 Hz), 4.47 (1H, dd, *J* = 7.0, 5.9 Hz), 4.46 (1H, d, *J* = 11.6 Hz), 4.42 (1H, d, *J* = 11.6 Hz), 4.32 (1H, dd, *J* = 8.1, 7.2 Hz), 4.29 (1H, D_2O exch, d, *J* = 3.4 Hz, OH), 3.99 (1H, dd, *J* = 11.0, 2.7 Hz), 3.99-3.95 (2H, m), 3.92 (1H, dd, *J* = 10.9, 3.4 Hz), 3.76 (3H, s, OCH_3), 3.53 (1H, dd, *J* = 7.4, 3.4 Hz), 3.48 (1H, dd, *J* = 8.2, 7.5 Hz), 1.41 (3H, s, CH_3), 1.29 (3H, s, CH_3). ^{13}C NMR (100 MHz, acetone-d$_6$): δ = 166.2 (C), 160.0 (C), 152.7 (C), 152.6 (CH), 151.0 (C), 145.6 (CH), 139.7 (C), 139.0 (C), 134.4 (CH), 132.8 (CH), 132.0 (C), 130.6 (CH), 130.3 (C), 130.1 (CH), 129.5 (CH), 129.2 (CH), 128.9 (CH), 128.5 (CH), 128.4 (CH), 128.1 (CH), 114.2 (CH), 110.0 (C), 87.9 (CH), 84.2 (CH), 83.8 (CH), 83.2 (CH), 80.7 (CH), 79.4 (CH), 78.4 (CH), 77.9 (CH), 76.6 (CH), 74.0 (OCH_2), 73.3 (OCH_2), 73.0 (OCH_2), 72.3 (CH), 70.0 (OCH_2), 55.5 (OCH_3), 28.1 (CH_3), 25.6 (CH_3). HRMS (ES): m/z found 893.3178, $C_{48}H_{50}ClN_4O_{11}^+$ [M+H]$^+$ requires 893.3159.

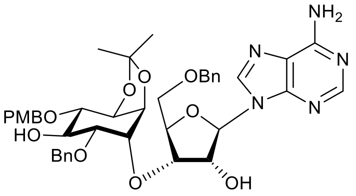

12: 3′-*O*-(2″-*O*-Benzyl-5″,6″-*O*-isopropylidene-4″-*O*-*p*-methoxybenzyl-1″-D-*chiro*-inosityl)-5′-*O*-benzyladenosine. Compound **36** (125 mg, 0.14 mmol) was dissolved in EtOH (8 mL) and the solution placed into a sealed tube equipped with a magnetic stirring bar. The mixture was cooled to 0 °C and ammonia was slowly bubbled into the solution via a glass pipette for about 30 min. The tube was sealed and the mixture was stirred at 70 °C for 18 h. The reaction mixture was cooled to rt and the vessel very carefully opened in order to release the excess ammonia. The remaining solvent was evaporated and the residue purified by flash column chromatography CH_2Cl_2 and 1% Et_3N to CH_2Cl_2/MeOH 19:1 and 1% Et_3N) to afford compound **12** as colourless glass (75 mg; 69%). $[\alpha]_D^{22}$ = +5.0° (c = 0.7; MeOH). ^1H NMR (400 MHz, acetone-d_6): δ = 8.22 (1H, s, CH), 8.21 (1H, s, CH), 7.43-7.21 (12H, m, 12 x CH), 6.89 (2H, d, *J* = 8.7 Hz, 2 x CH), 6.72 (2H, s, br, NH_2), 6.05 (1H, d, *J* = 3.6 Hz), 4.89 (1H, d, *J* = 11.7 Hz), 4.82 (1H, d, *J* = 3.7 Hz), 4.79 (1H, d, *J* = 4.1 Hz), 4.76 (1H, t, *J* = 4.1 Hz), 4.72 (1H, d, *J* = 11.5 Hz), 4.68 (1H, dd, *J* = 5.6, 5.0 Hz), 4.65 (1H, d, *J* = 12.0 Hz), 4.59 (1H, d, *J* = 12.0 Hz), 4.39 (1H, dd, *J* = 5.8, 3.9 Hz), 4.28 (1H, dt, *J* = 5.8, 3.5 Hz), 4.24 (1H, dd, *J* = 7.2, 5.9 Hz), 4.21 (1H, t, *J* = 3.3 Hz), 4.03 (1H, t, *J* = 8.4 Hz), 3.85 (1H, dd, *J* = 10.9, 3.3 Hz), 3.80-3.76 (1H, m), 3.78 (3H, s, OCH_3), 3.74 (1H, dd, *J* = 8.1, 2.9 Hz), 3.49 (1H, dd, *J* = 8.6, 7.5 Hz), 1.40 (3H, s, CH_3), 1.29 (3H, s, CH_3); two OH not visible. ^{13}C NMR (100 MHz, acetone-d_6): δ = 160.0 (C), 157.0 (C), 153.8 (CH), 150.6 (C), 140.0 (CH), 139.3 (C), 139.1 (C), 132.1 (C), 130.2 (CH), 129.2 (CH), 129.1 (CH), 128.9 (CH), 128.5 (CH), 128.4 (CH), 128.4 (CH), 120.4 (C), 114.2 (CH), 110.1 (C), 89.4 (CH), 84.6 (CH), 82.3 (CH), 80.2 (CH), 80.0 (CH), 79.7 (CH), 78.1 (CH), 76.4 (CH), 74.4 (OCH_2), 74.3 (CH), 73.9 (OCH_2), 73.5 (OCH_2), 72.6 (CH), 70.1 (OCH_2), 55.5 (OCH_3), 28.2 (CH_3), 26.2 (CH_3). HRMS (ES): m/z found 770.3375, $C_{41}H_{48}N_5O_{10}^+$ $[M+H]^+$ requires 770.3396.

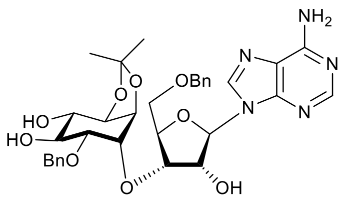

11: 3′-*O*-(2″-*O*-Benzyl-5″,6″-*O*-isopropylidene-1″-D-*chiro*-inosityl)-5′-*O*-benzyladenosine. Compound **12** (70 mg, 0.09 mmol were dissolved in super dry CH_2Cl_2 (0.75 mL). Then TFA (~170 mg, 1.5 mmol) in super dry CH_2Cl_2 (0.75 mL) was added and the reaction mixture was stirred for 5 min at rt. The reaction mixture diluted with super dry CH_2Cl_2 (10 mL) and ammonia (7M in MeOH, 1.0 mL) was added via syringe. The reaction mixture was then further diluted with CH_2Cl_2 (100 mL) and washed with water (100 mL). The organic layer was dried by filtration through solid NaCl and concentrated in vacuum. Purification by flash column chromatography (CH_2Cl_2 and 1% Et_3N to CH_2Cl_2/MeOH 19:1 and 1% Et_3N to CH_2Cl_2/MeOH 9:1 and 1% Et_3N) to afford compound **11** as colourless glass (37 mg; 63%). A small amount was recrystallized from acetone to afford **11** as a white solid (7.5 mg; 13%). $[\alpha]_D^{22}$ = +10.9° (c = 0.44; acetone). ^{1}H NMR (400 MHz, acetone-d_6): δ = 8.21 (2H, s, CH), 7.44-7.21 (10H, m, 10 x CH), 6.68 (2H, D_2O exch, s, br, NH_2), 6.05 (1H, d, *J* = 3.6 Hz), 4.89 (1H, d, *J* = 11.8 Hz), 4.80 (1H, d, *J* = 11.8 Hz), 4.76 (1H, dd, *J* = 4.4, 4.0 Hz), 4.69 (1H, dd, *J* = 5.6, 5.2 Hz), 4.66 (1H, d, *J* = 12.4 Hz), 4.60 (1H, d, *J* = 12.4 Hz), 4.59 (1H, D_2O exch, d, *J* = 4.4 Hz, OH), 4.55, (1H, D_2O exch, s, br, OH), 4.43 (1H, D_2O exch, s, br, OH), 4.37 (1H, dd, *J* = 5.7, 3.6 Hz), 4.27 (1H, dt, *J* = 5.7, 3.5 Hz), 4.21 (1H, t, *J* = 3.4 Hz), 4.10 (1H, dd, *J* = 7.5, 5.8 Hz), 3.90 (1H, t, *J* = 8.6 Hz), 3.85 (1H, dd, *J* = 10.8, 3.3 Hz), 3.77 (1H, dd, *J* = 10.8, 3.7 Hz), 3.70 (1H, dd, *J* = 8.4, 2.9 Hz), 3.56 (1H, dd, *J* = 8.6, 7.8 Hz), 1.39 (3H, s, CH_3), 1.27 (3H, s, CH_3). ^{13}C NMR (100 MHz, acetone-d_6): δ = 157.1 (C), 153.7 (CH), 150.6 (C), 139.9 (CH), 139.3 (C), 139.1 (C), 129.2 (CH), 129.1 (CH), 128.9 (CH), 128.5 (CH), 128.4 (CH), 128.4 (CH), 120.5 (C), 109.9 (C), 89.4 (CH), 82.3 (CH), 80.2 (CH), 80.1 (CH), 79.9 (CH), 78.2 (CH), 77.1 (CH), 76.3 (CH), 74.5 (CH), 74.4 (OCH_2), 73.9 (OCH_2), 73.2 (CH), 70.1 (OCH_2), 28.3 (CH_3), 26.2 (CH_3). HRMS (ES): m/z found 672.2623, $C_{33}H_{39}NaN_5O_9^+$ [M+Na]$^+$ requires 672.2640.

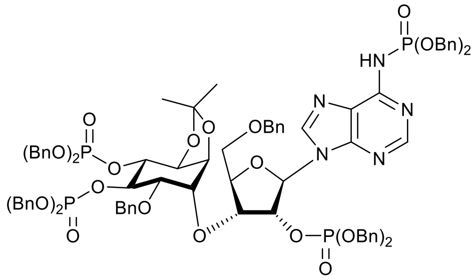

38: 5′-*O*-Benzyl-2′-*O*-dibenzylphosphoryl-6-*N*-dibenzylphosphoryl-3′-*O*-(2″-*O*-benzyl-3″,4″-dibenzylphosphoryl-5″,6″-*O*-isopropylidene-1″-D-*chiro*-inositol)-adenosine. Compound **11** (7.5 mg, 11.5 µmol) and 5-phenyl-*1H*-tetrazole (20 mg, 0.137 mmol) were placed in a 10 mL round-bottom flask and super dry CH_2Cl_2 (0.3 mL) was added. The dispersion was stirred for 5 min at rt. Dibenzyl diisopropylphosphoramidite (40 mg, 0.124 mmol) was added and the reaction mixture was stirred for 30 min. After the conversion was complete (by ^{31}P NMR), *tert*-butyl hydroperoxide (70% in water, 75 mg, 0.58 mmol) was added and the mixture stirred for another 30 min at rt. The mixture was then diluted with water (40 mL) and extracted with CH_2Cl_2 (2 x 40 mL). The combined organic layers were washed with Na_2SO_3 (sat., 10 mL), $NaHCO_3$ (sat., 10 mL), dried by filtration through solid NaCl and concentrated in vacuum. Purification by flash column chromatography (CH_2Cl_2 to CH_2Cl_2/MeOH 19:1) afforded compound **38** as a colourless oil (13.5 mg; 69%). $[\alpha]_D^{22}$ = –3.7° (c = 0.72; acetone). ^{1}H NMR (400 MHz, acetone-d_6): δ = 8.89 (1H, s, br, NH), 8.50 (1H, s, CH), 8.46 (1H, s, CH), 7.47-7.15 (50H, m, 50 x CH), 6.40 (1H, d, *J* = 4.7 Hz), 5.63 (1H, dt, *J* = 8.0, 4.7 Hz), 5.33-5.22 (4H, m), 5.19-5.12 (4H, m), 5.12 (1H, d, *J* = 4.0 Hz), 5.11 (1H, d, *J* = 4.1 Hz), 5.09 (1H, d, *J* = 1.5 Hz), 5.07 (1H, d, *J* = 2.1 Hz), 5.00 (1H, d, *J* = 1.9 Hz), 4.98 (1H, d, *J* = 1.8 Hz), 4.93 (1H, d, *J* = 2.6 Hz), 4.91 (1H, d, *J* = 2.2 Hz), 4.83 (1H, t, *J* = 4.7 Hz), 4.80-4.68 (4H, m), 4.68-4.59 (3H, m), 4.51 (1H, dd, *J* = 9.3, 7.1 Hz), 4.46 (1H, dt, *J* = 4.6, 2.9 Hz), 4.43 (1H, s), 4.22 (1H, dd, *J* = 6.7, 2.0 Hz), 3.90 (1H, dd, *J* = 10.9, 2.6 Hz), 3.82 (1H, dd, *J* = 10.9, 3.3 Hz), 1.41 (3H, s, CH_3), 1.25 (3H, s, CH_3). ^{31}P NMR (162 MHz, 1H-decoupled, acetone-d_6): δ = -0.9, -1.2, -1.5, -1.5. HRMS (ES): m/z found 1690.5183, $C_{89}H_{92}N_5O_{21}P_4^+$ $[M+H]^+$ requires 1690.5230.

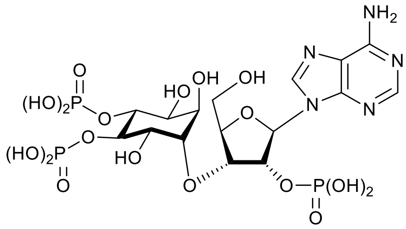

10: 3′-*O*-(1″-D-*chiro*-InositGyl)-adenosine 2′,3″,4″-trisphosphate, [D-*chiro*-Inositol adenophostin]. Compound **38** (13.5 mg, 8.0 µmol) was dissolved in MeOH (3.5 mL). Then water (0.5 mL), cyclohexene (0.5 mL, ~5 mmol) and $Pd(OH)_2$ (42 mg, 20% Pd on charcoal, 50% water, ~40 µmol) were added and the reaction mixture was stirred at 70 °C for 18 h. The reaction mixture was cooled to rt, filtered through PTFE filter and concentrated in vacuum. The residue was then treated with hot MeOH (~3 mL) affording a small amount of white solid (~1.5-2 mg) that was further purified by semi-preparative HPLC. Compound **10** was afforded as a TEAB salt (~550 nmol by UV quantification, 7%, >99% pure by analytical HPLC; on other occasions up to 37% yield were achieved). ^{1}H NMR (400 MHz, ^{31}P-coupled, methanol-d_4): δ = 8.34 (1H, s, CH), 8.18 (1H, s, CH), 6.23 (1H, d, *J* = 6.0 Hz, H-1′), 5.30 (1H, dt, *J* = 7.8, 5.6 Hz, H-2′), 4.86 (1H, hidden, detectable by COSY, H-3′), 4.60 (1H, q, *J* = 9.3 Hz), 4.28 (1H, q, *J* = 9.1 Hz), 4.24-4.21 (1H, m, H-4′), 4.06-4.01 (2H, m), 4.00-3.97 (2H, m), 3.86 (1H, dd, *J* = 12.5, 3.0 Hz, H-5a′), 3.75 (1H, dd, *J* = 12.5, 3.1 Hz, H-5b′). ^{31}P NMR (202 MHz, ^{1}H-decoupled, methanol-d_4): δ = 2.80, 1.63, 0.61. ^{31}P NMR (202 MHz, ^{1}H-coupled, methanol-d_4): δ = 2.80 (d, *J* = 8.8 Hz), 1.63 (d, *J* = 8.8 Hz), 0.61 (d, *J* = 6.3 Hz). HRMS (ES): m/z found 668.0415, $C_{16}H_{25}N_5O_{18}P_3^-$ $[M–H]^-$ requires 668.0413.

^{1}H and ^{13}C NMR spectra.

Compound 15.

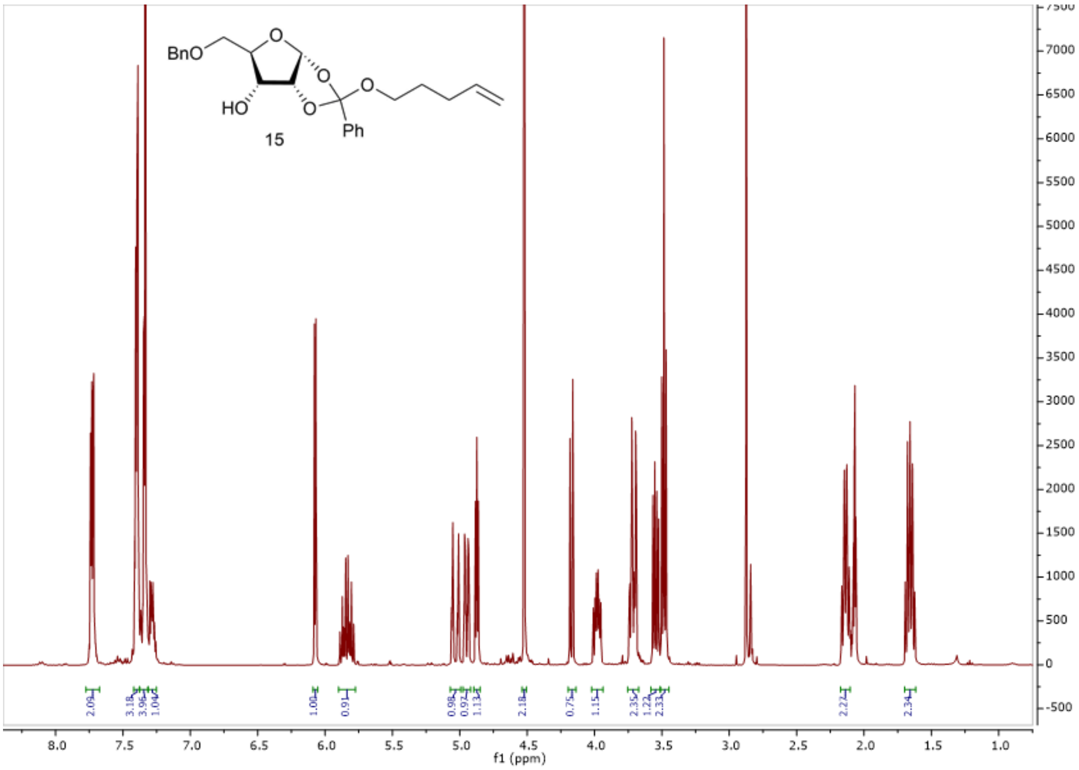

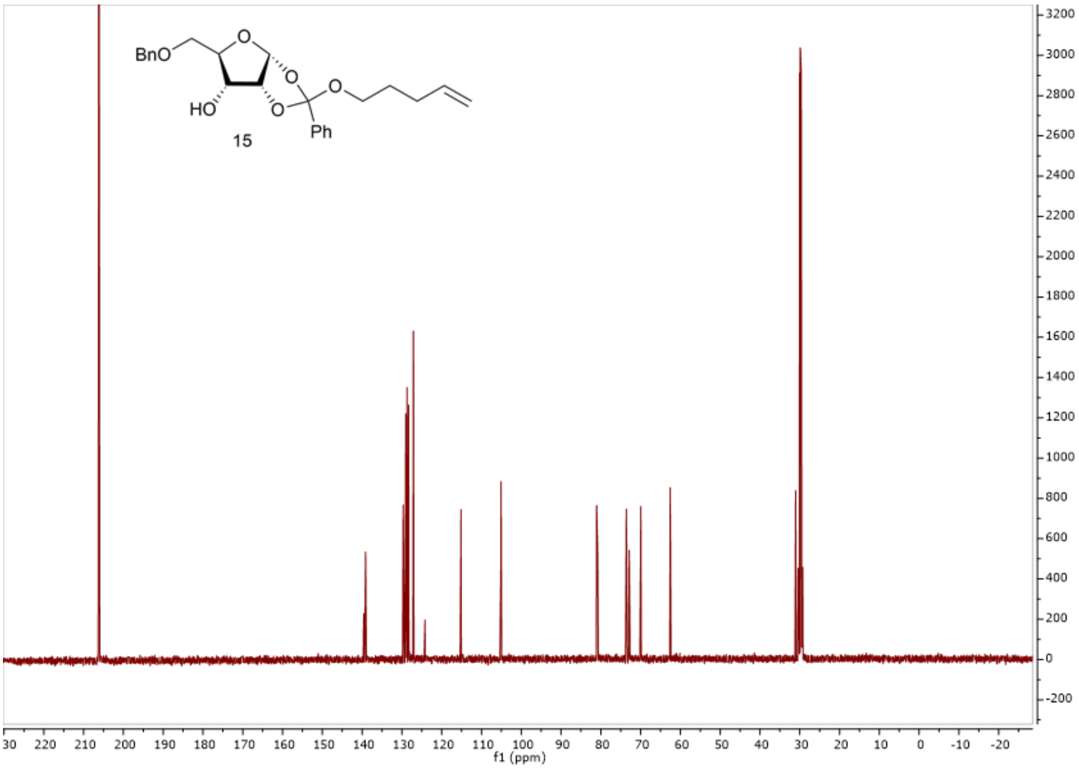

Compound 25.

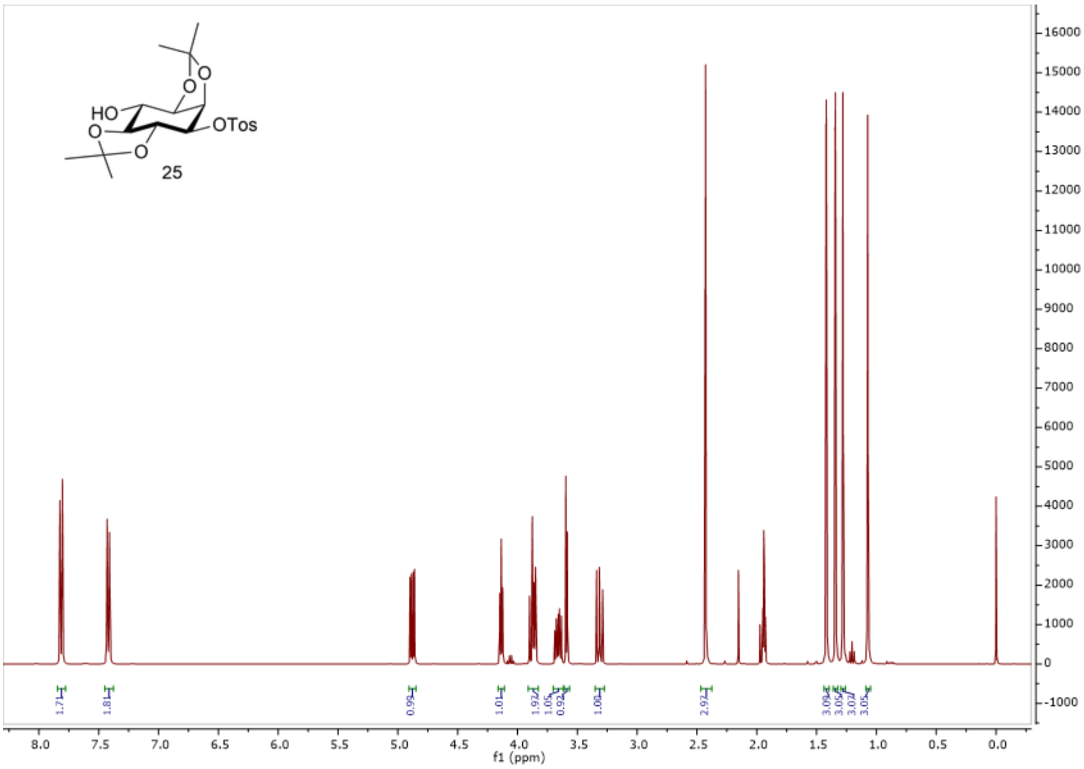

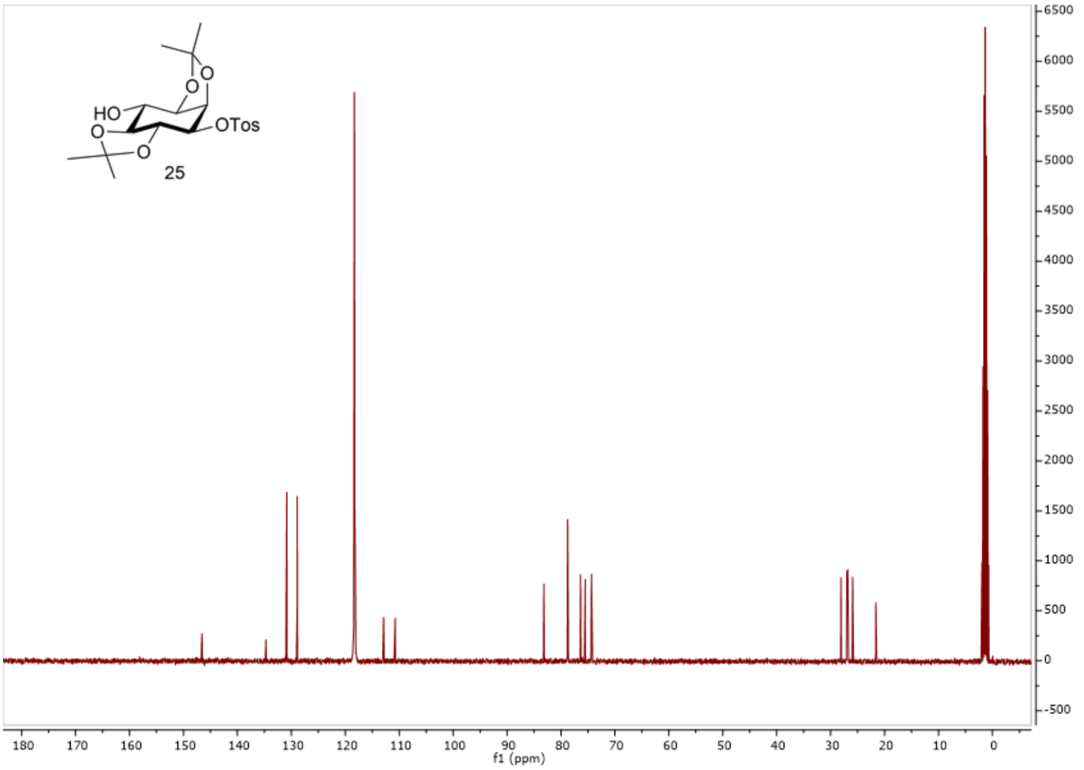

Compound 27.

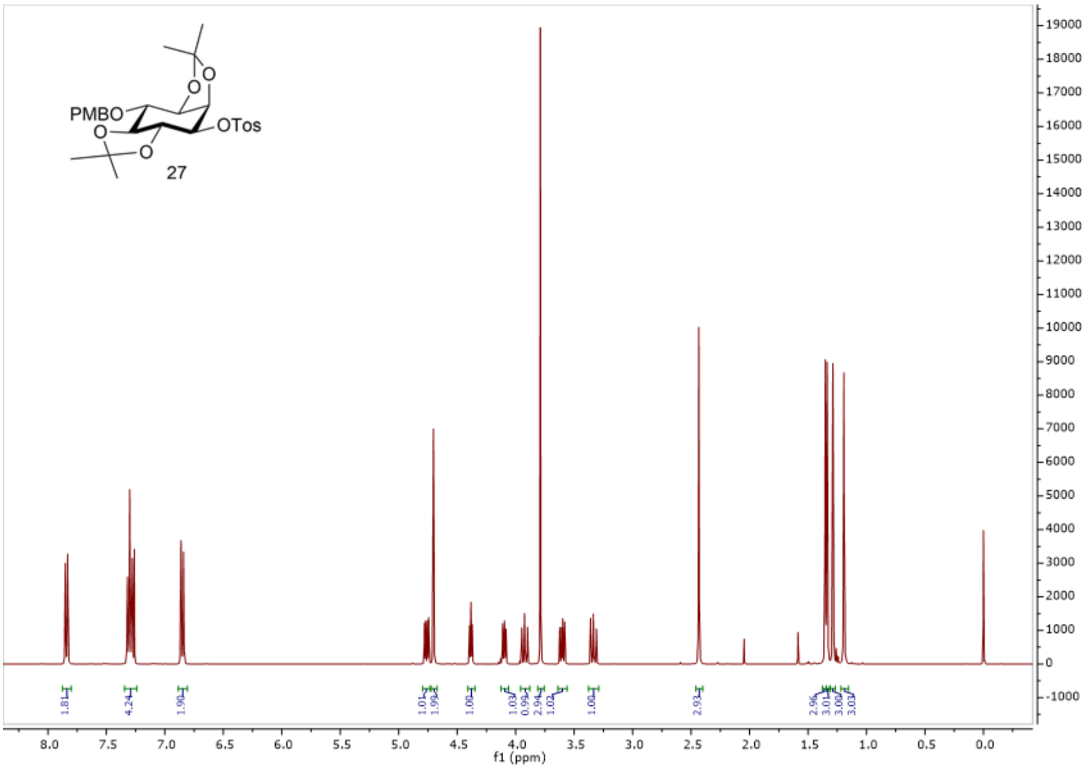

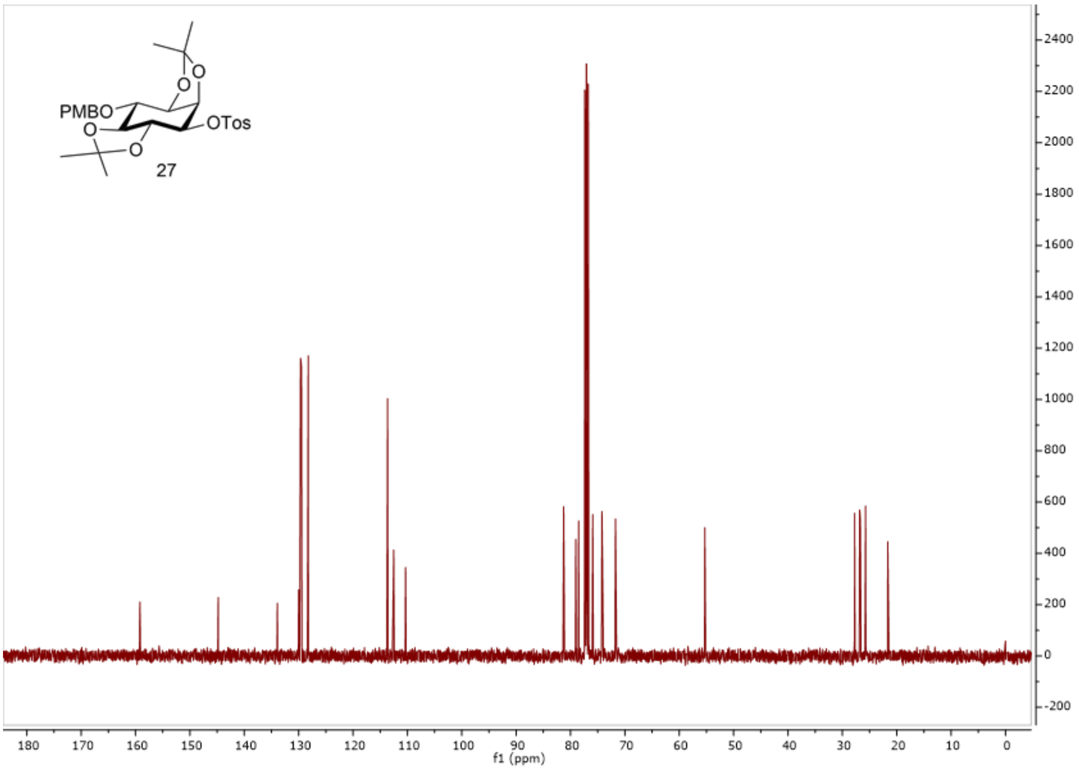

Compound 28.

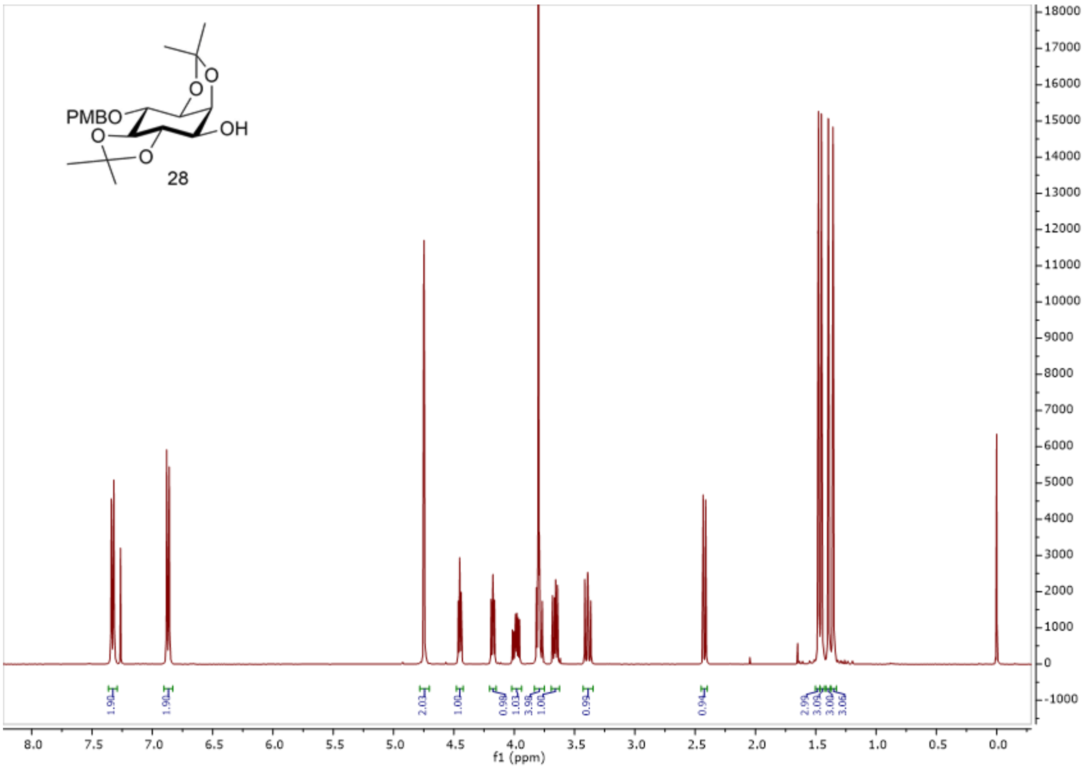

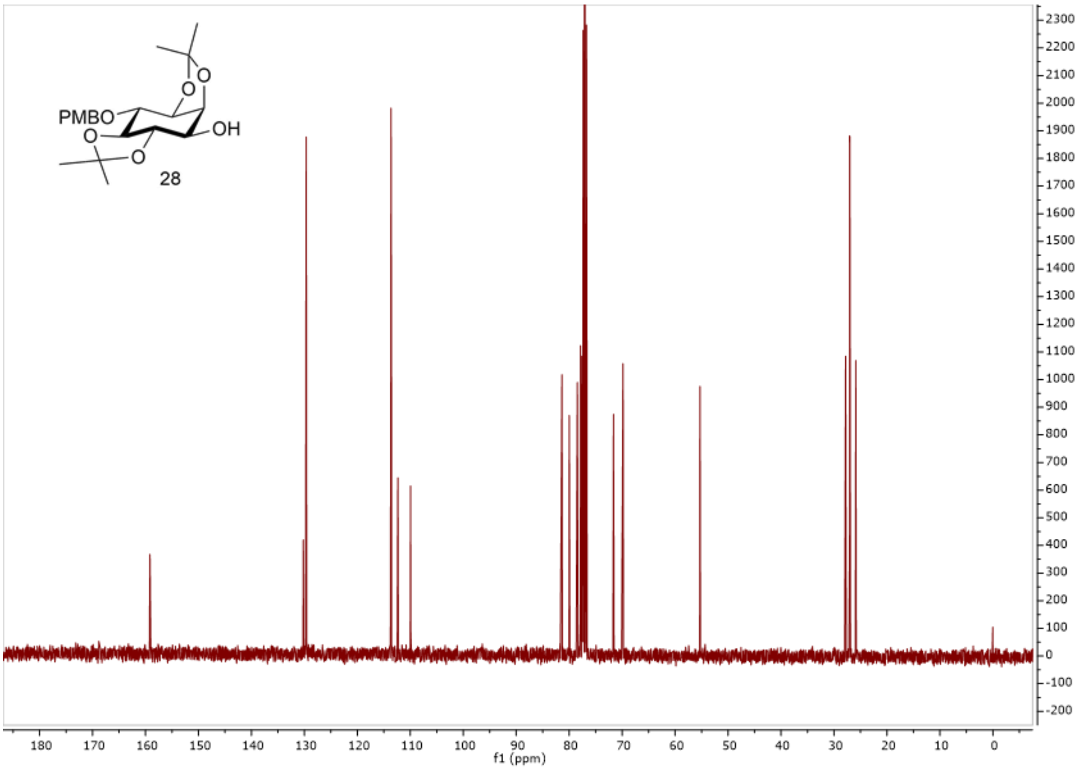

Compound 29.

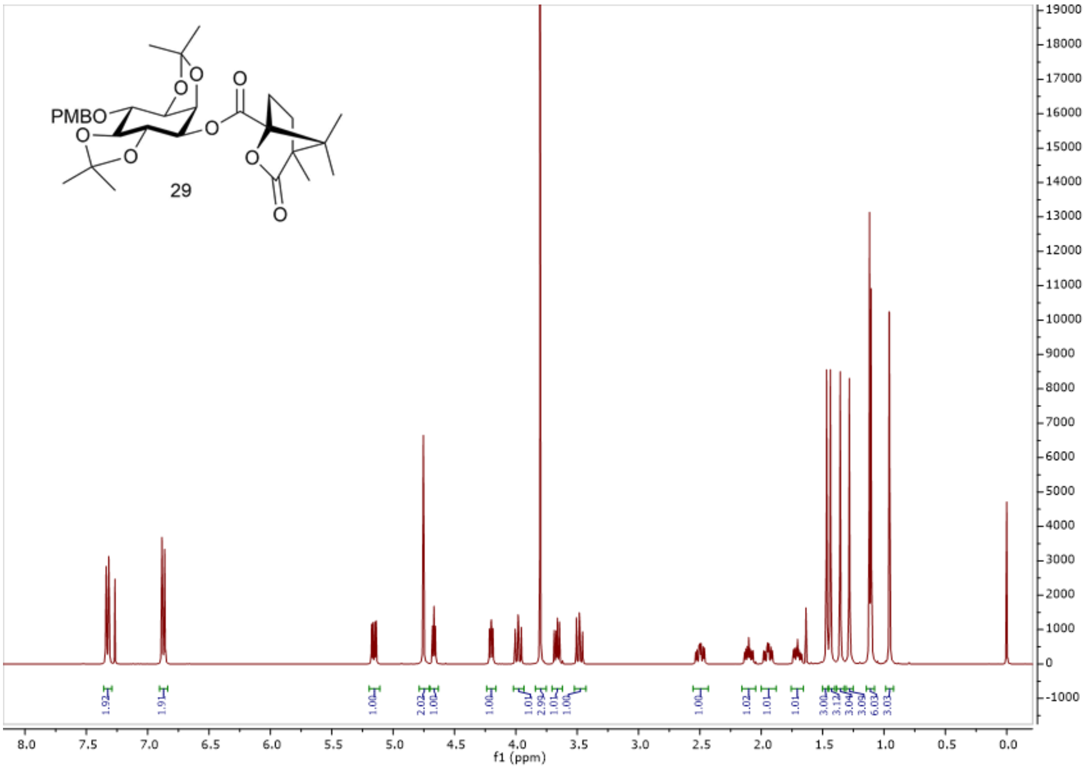

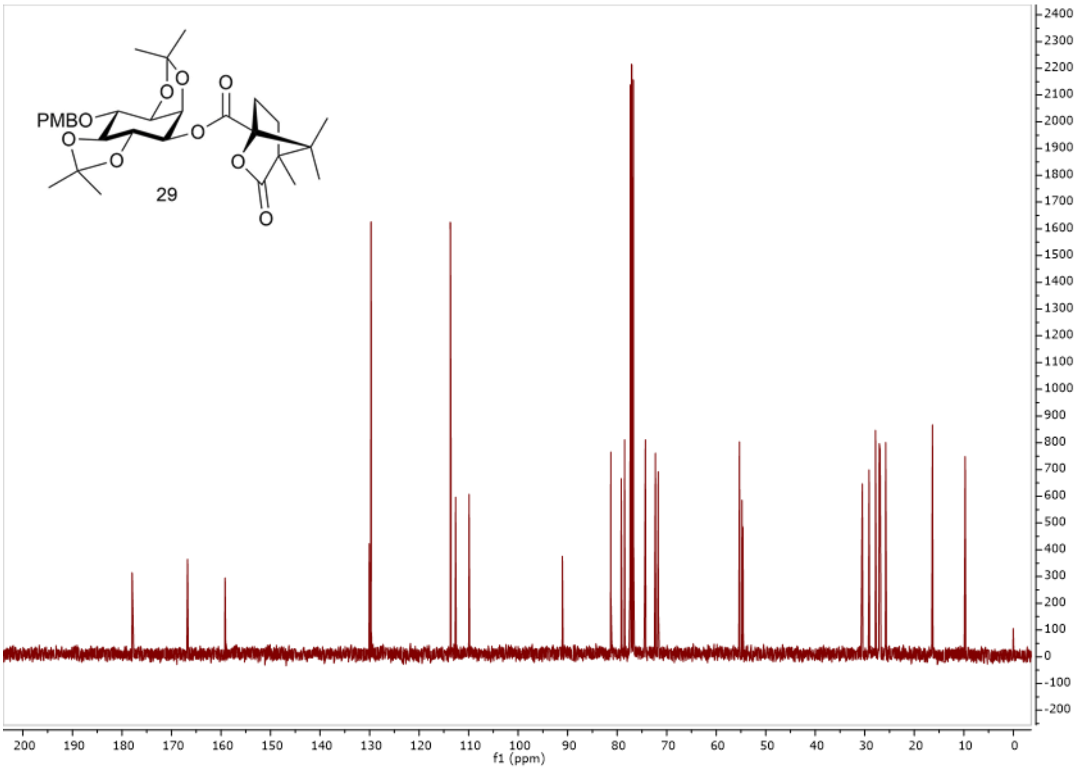

Compound 30.

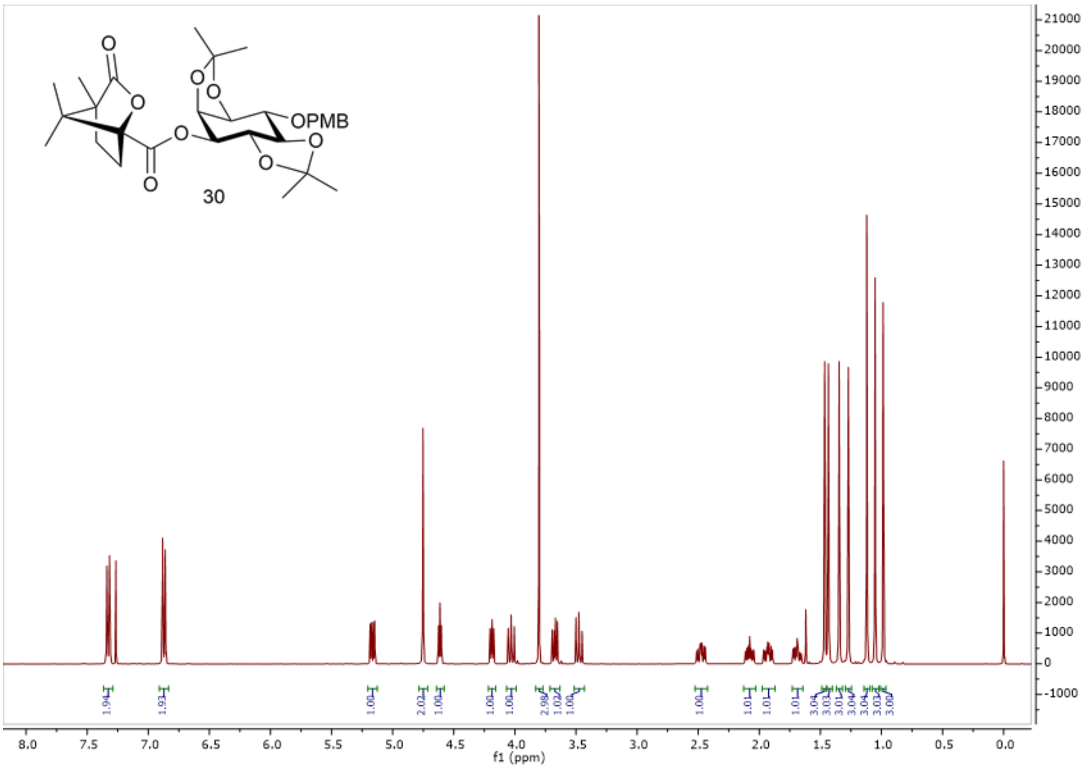

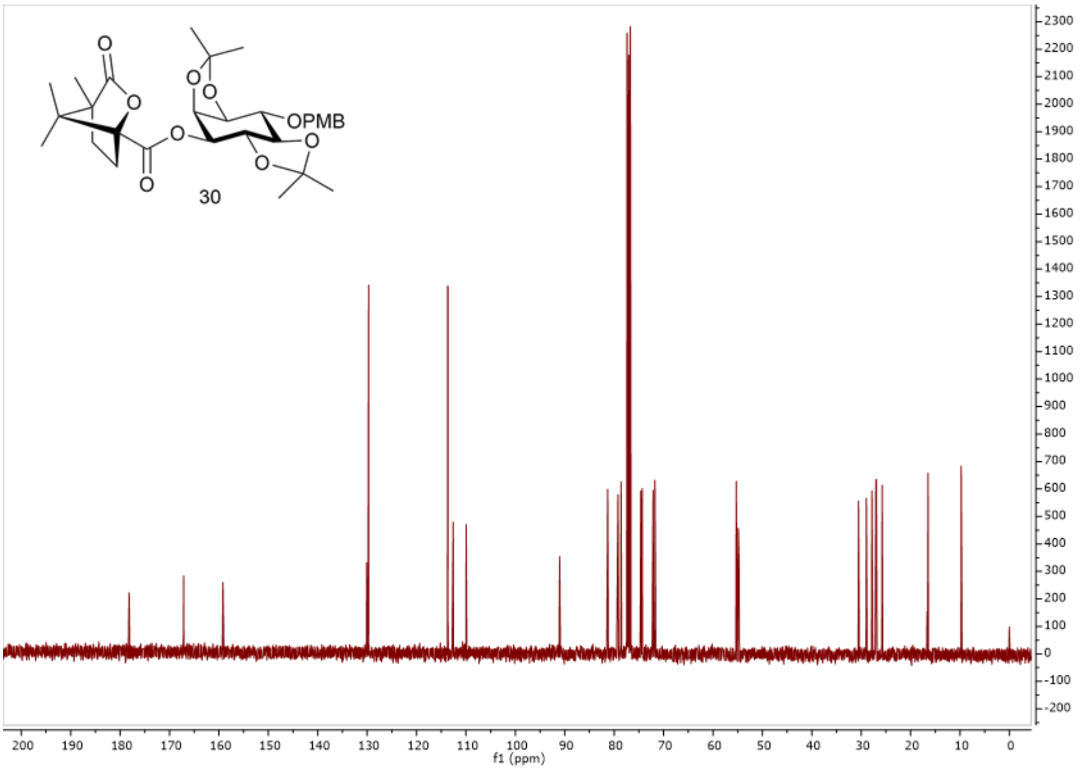

Compound 31.

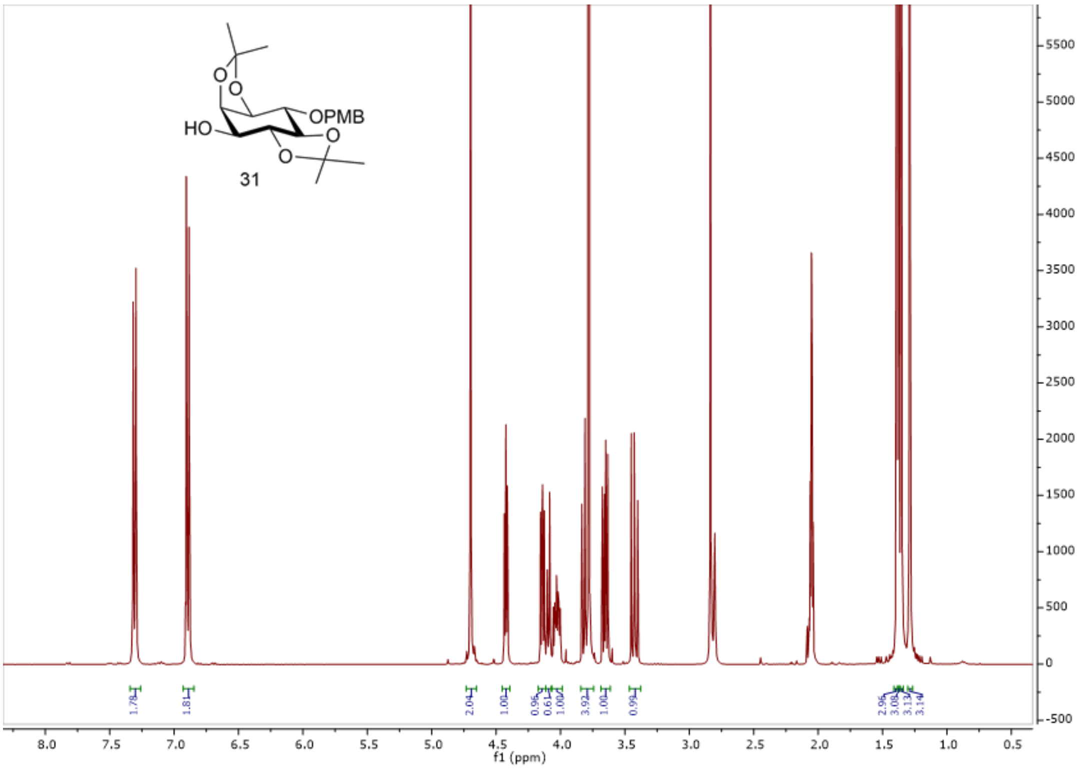

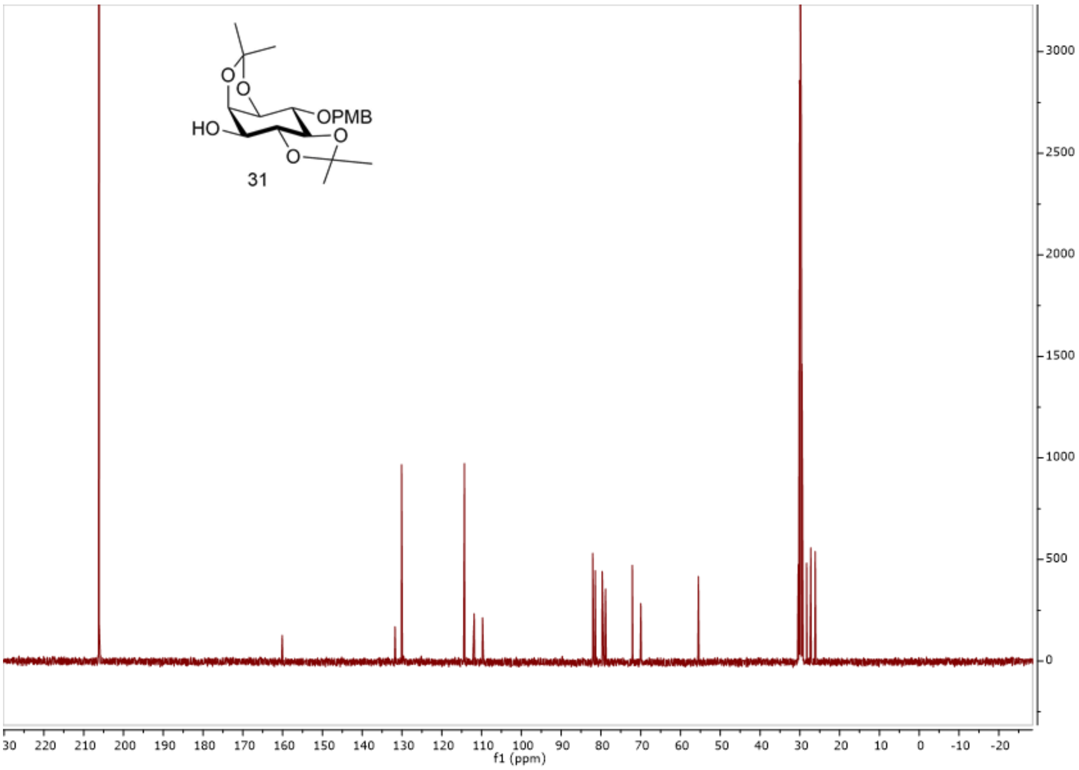

Compound 32.

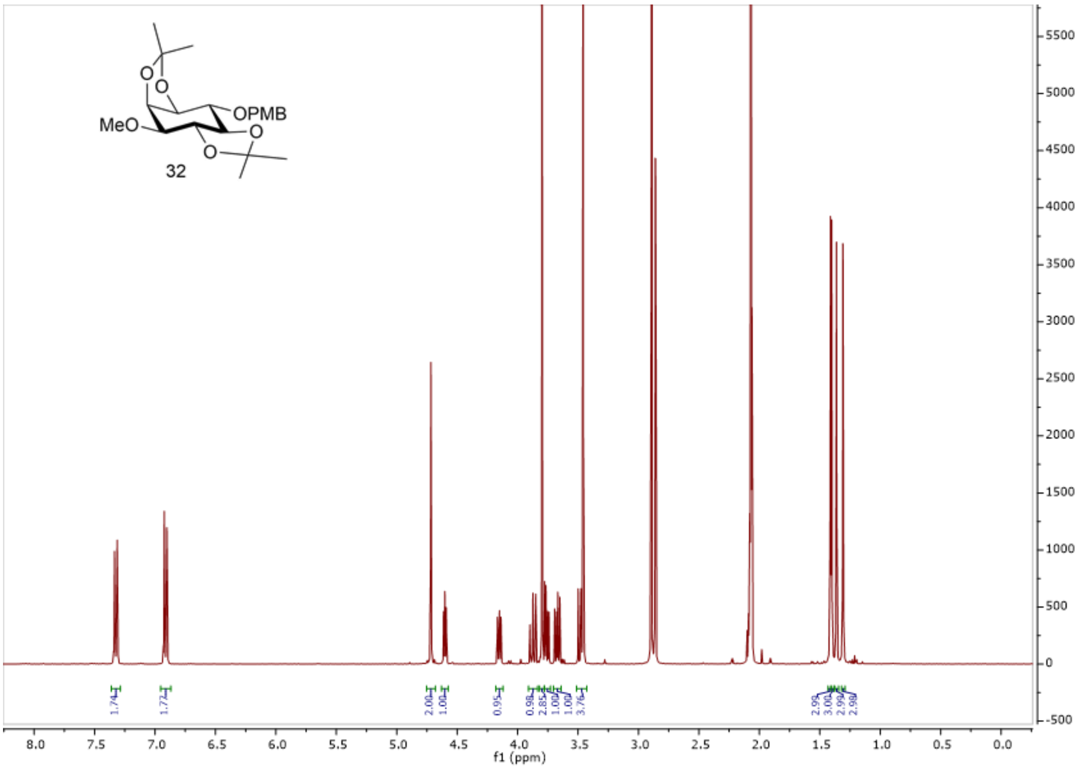
OPMB
MeO
32
1.74
1.72
2.00
1.00
0.95
0.98
2.85
1.00
1.00
3.76
2.99
3.00
2.99
2.98
f1 (ppm)

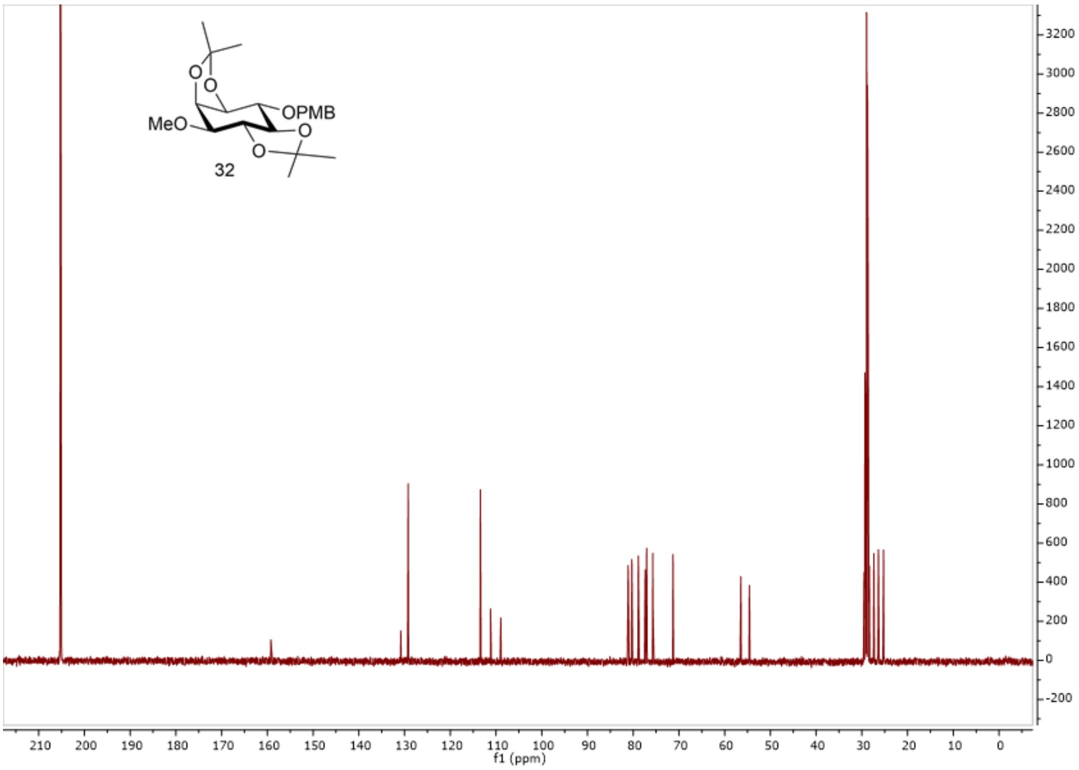
OPMB
MeO
32
f1 (ppm)

Compound 33.

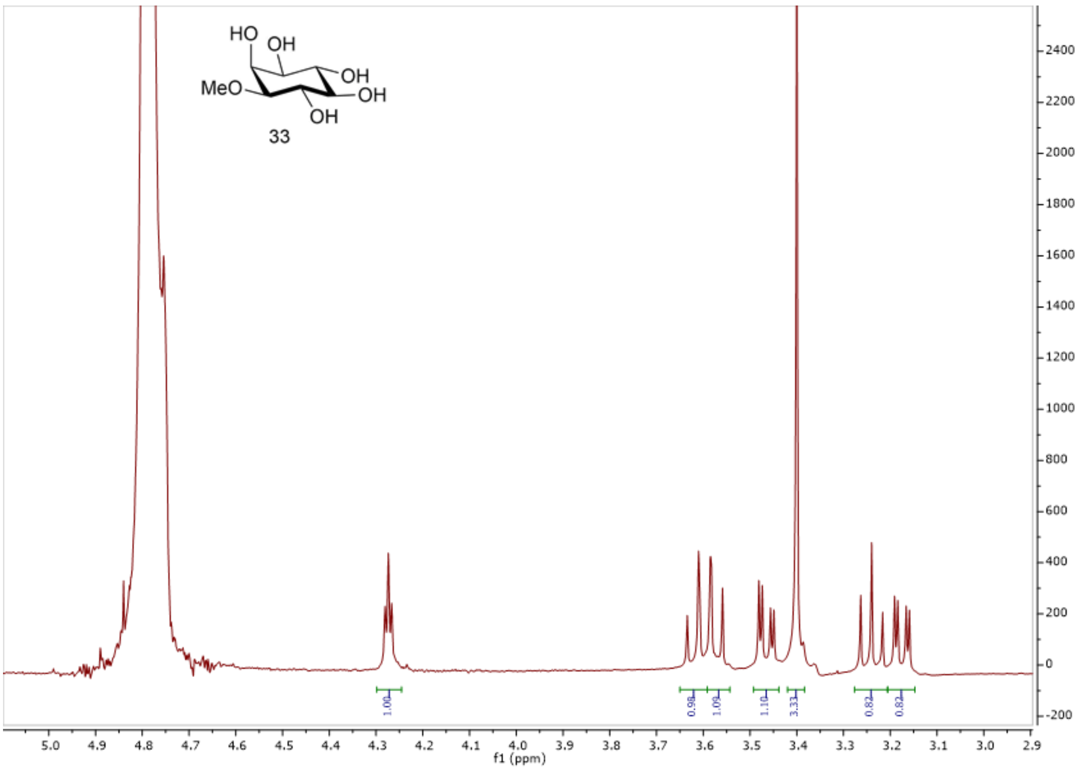

HO
OH
OH
MeO
OH
OH
33
1.00
0.98
1.09
1.10
3.33
0.82
0.82
f1 (ppm)

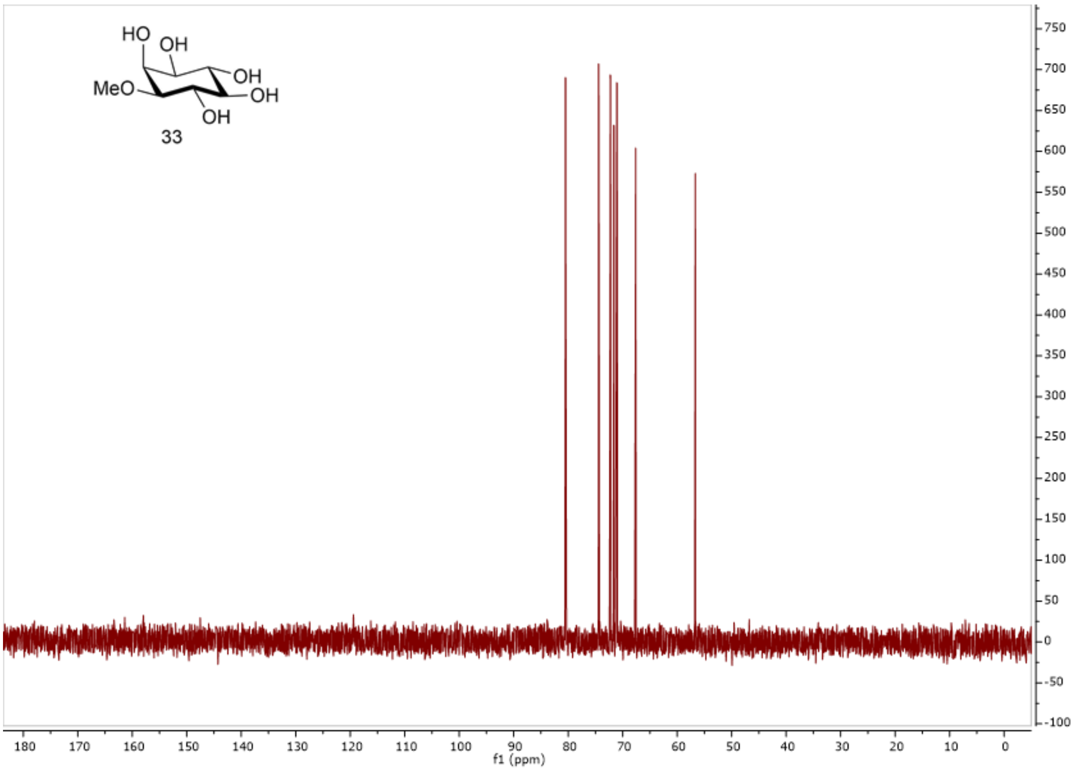

HO
OH
OH
MeO
OH
OH
33
f1 (ppm)

Compound 34.

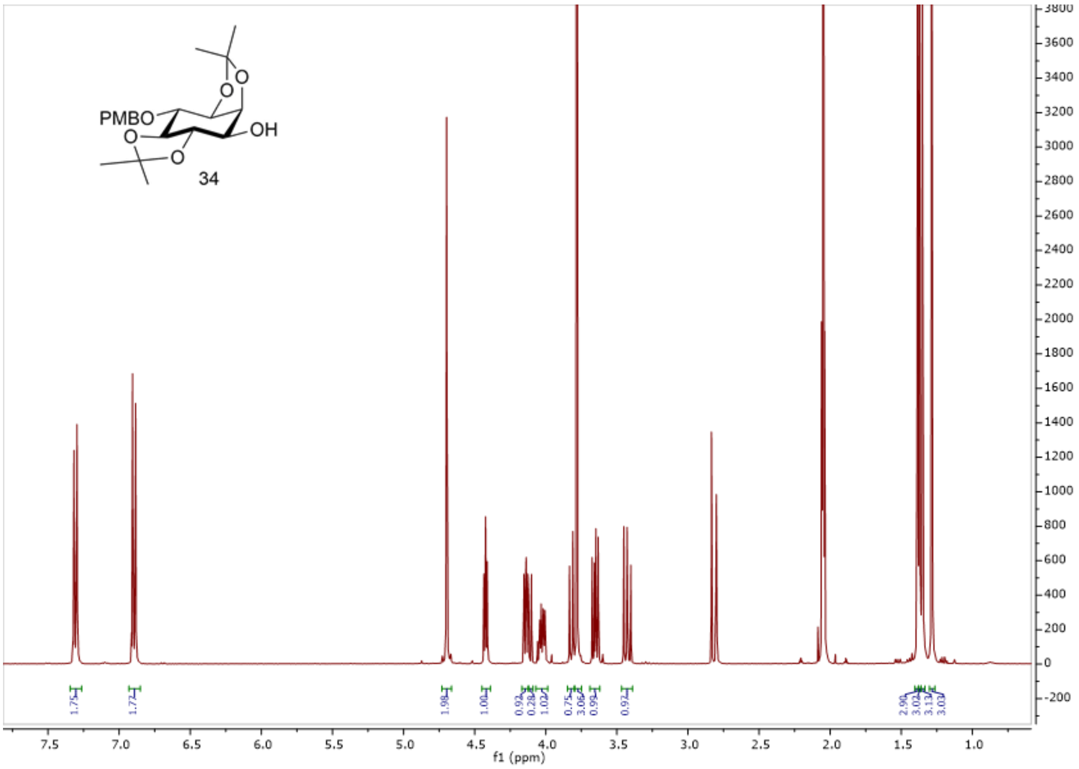

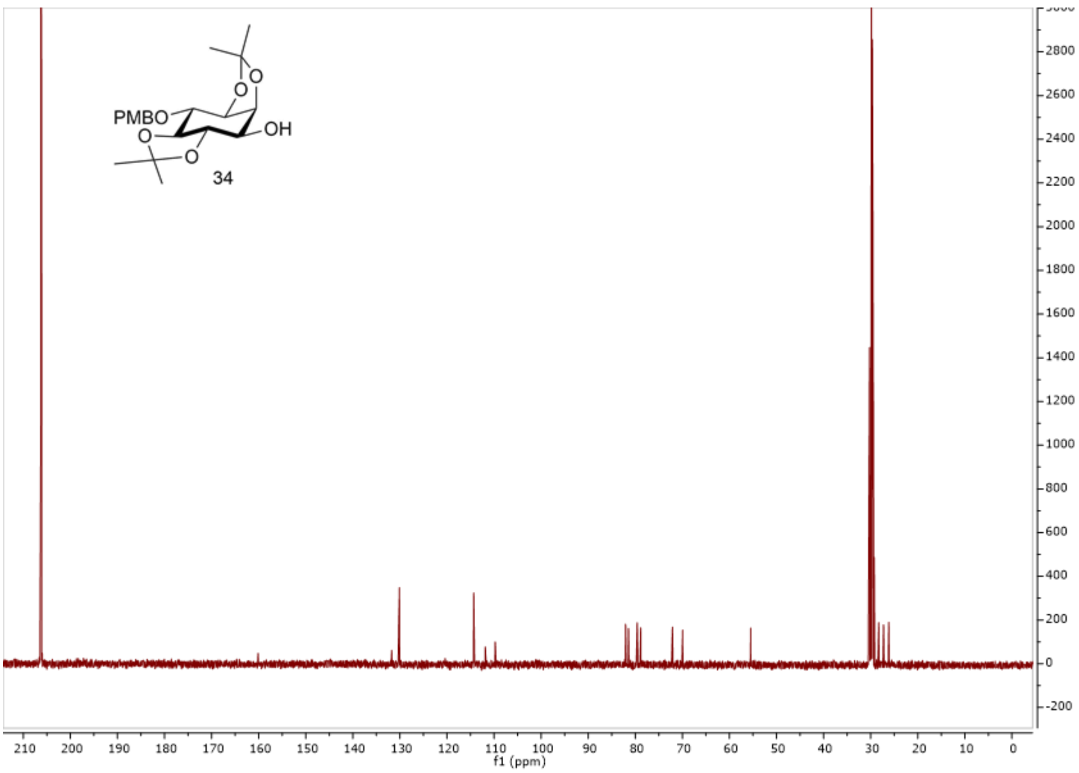

Compound 16.

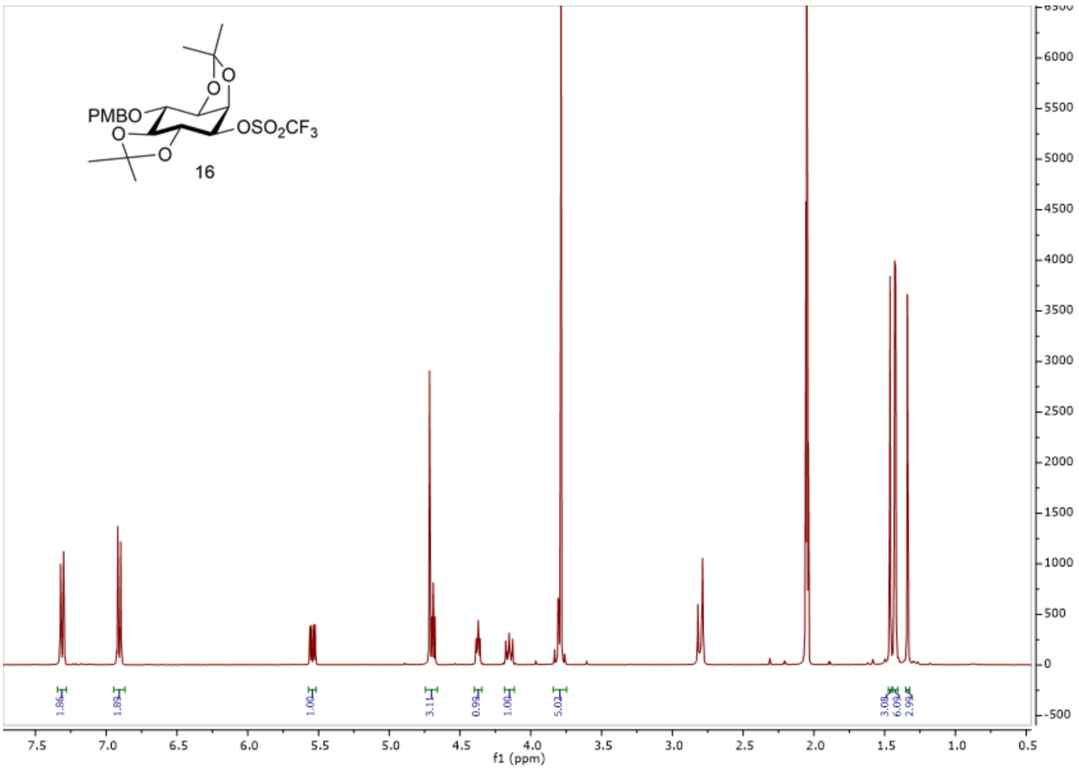

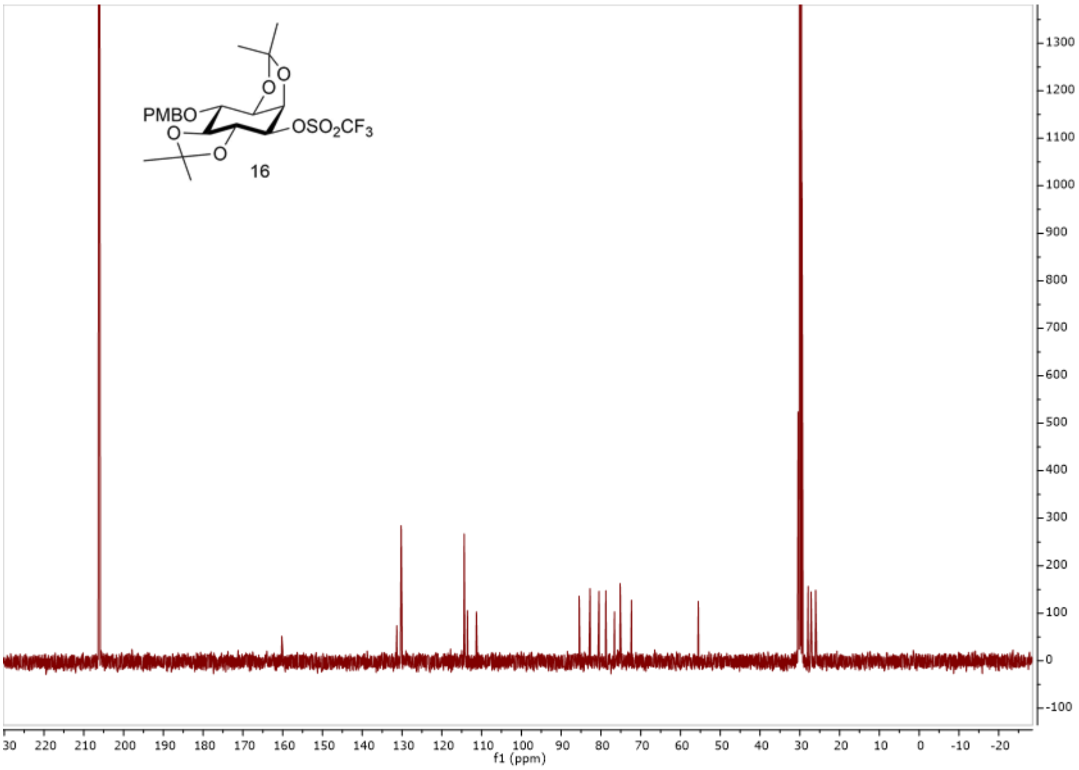

Compound 35.

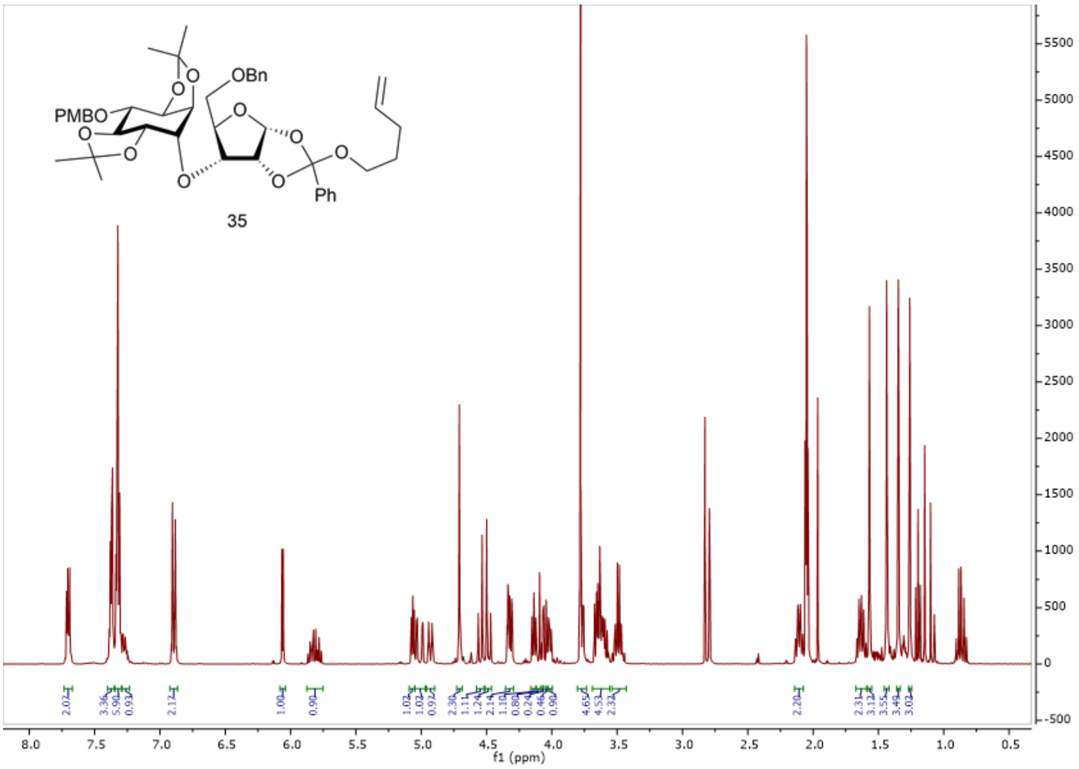

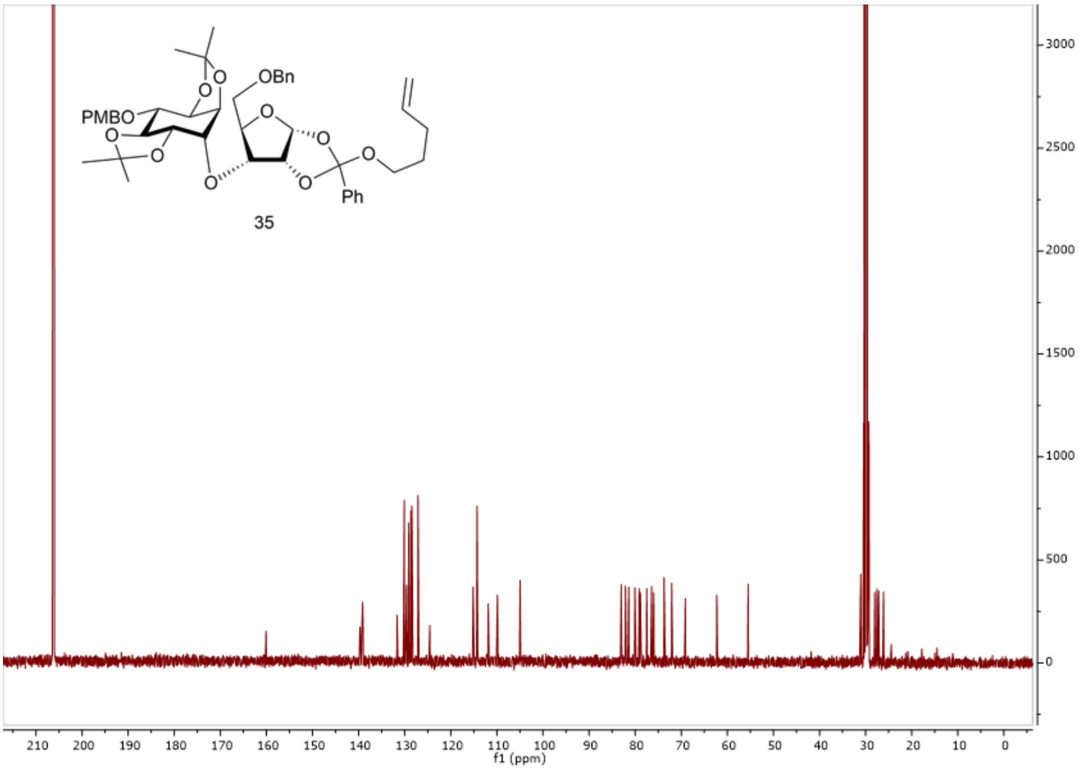

Compound 14.

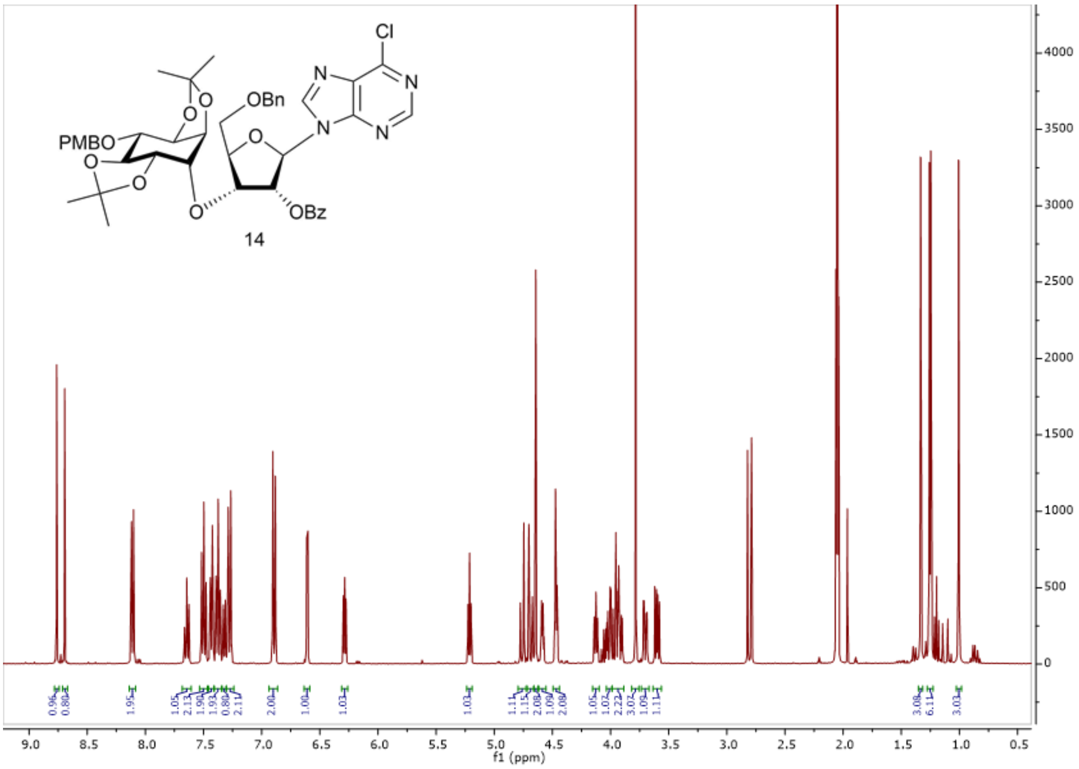
Cl
N
N
N
N
OBn
O
PMBO
O
O
O
O
O
OBz
14
4000
3500
3000
2500
2000
1500
1000
500
0
9.0
8.5
8.0
7.5
7.0
6.5
6.0
5.5
5.0
4.5
4.0
3.5
3.0
2.5
2.0
1.5
1.0
0.5
f1 (ppm)

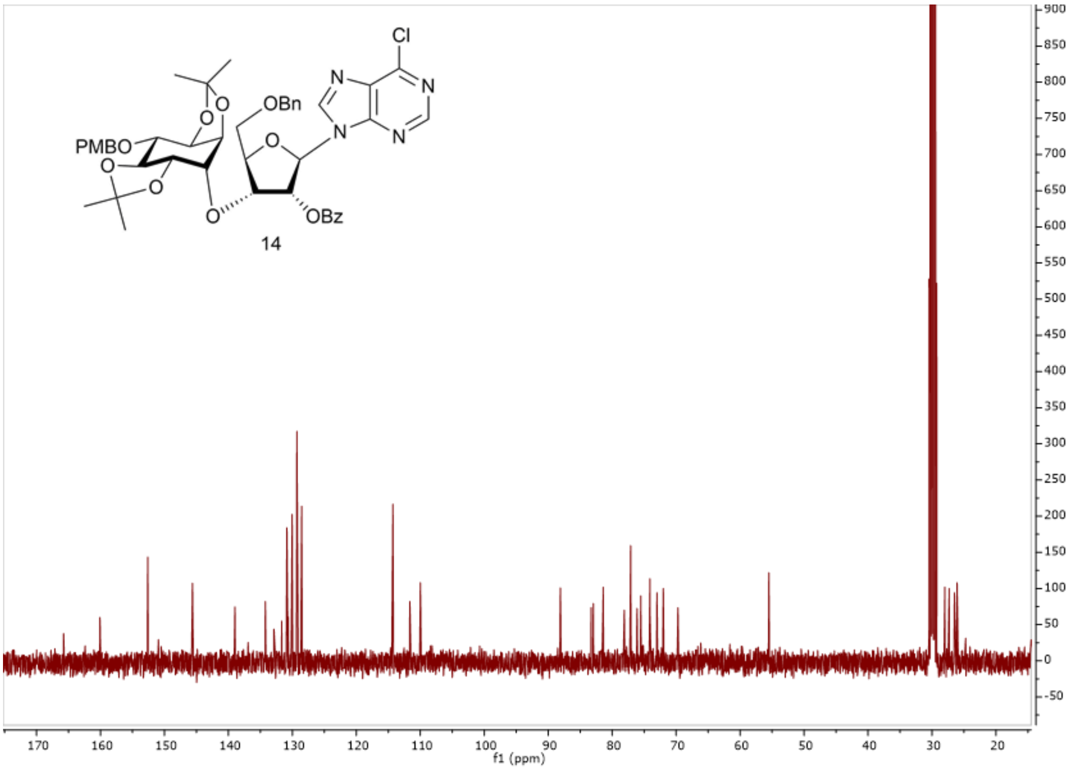
Cl
N
N
N
N
OBn
O
PMBO
O
O
O
O
O
OBz
14
900
850
800
750
700
650
600
550
500
450
400
350
300
250
200
150
100
50
0
-50
170
160
150
140
130
120
110
100
90
80
70
60
50
40
30
20
f1 (ppm)

Compound 13.

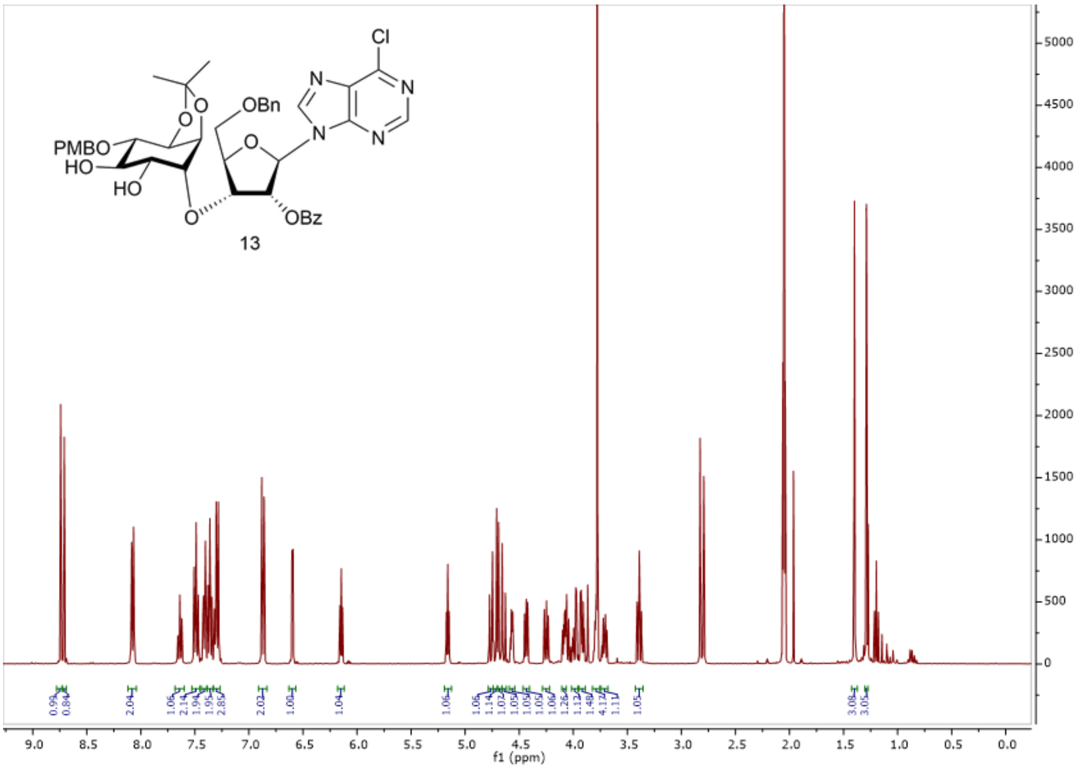

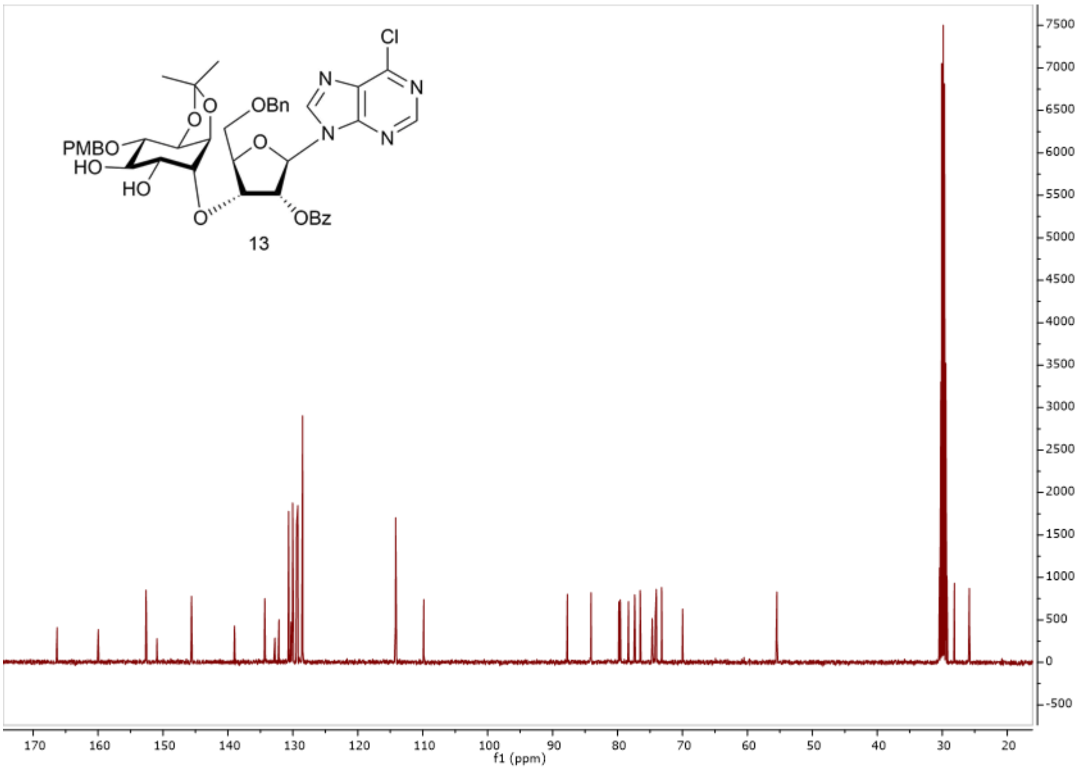

Compound 36.

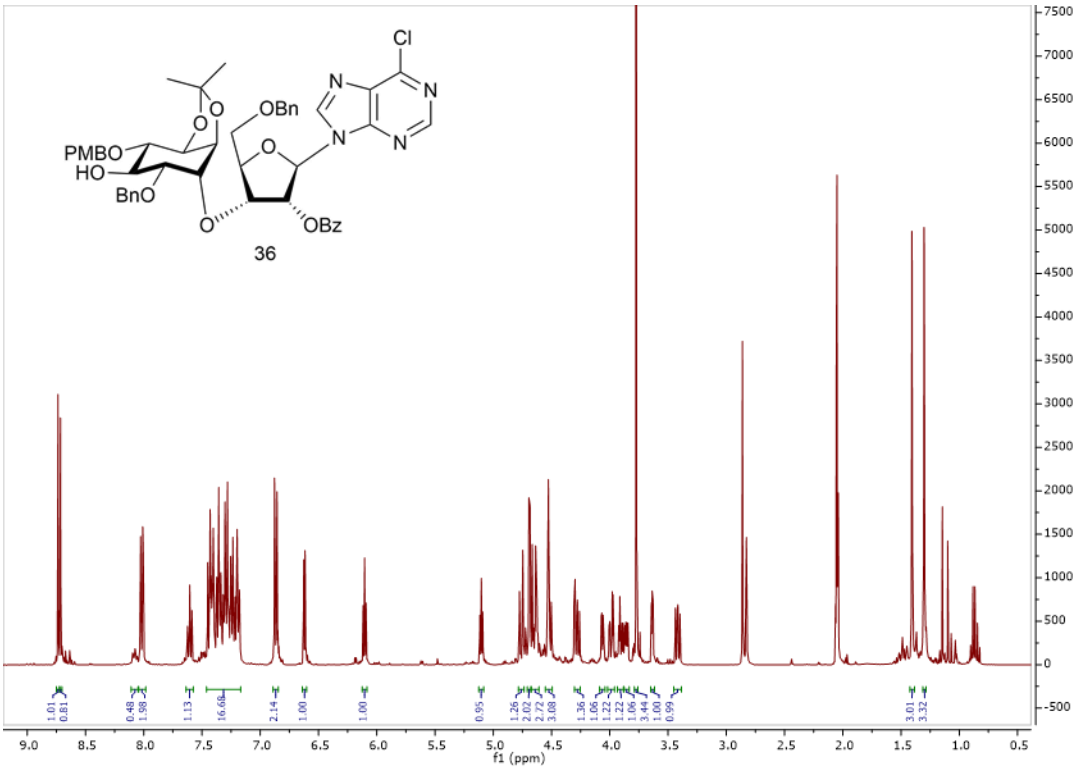

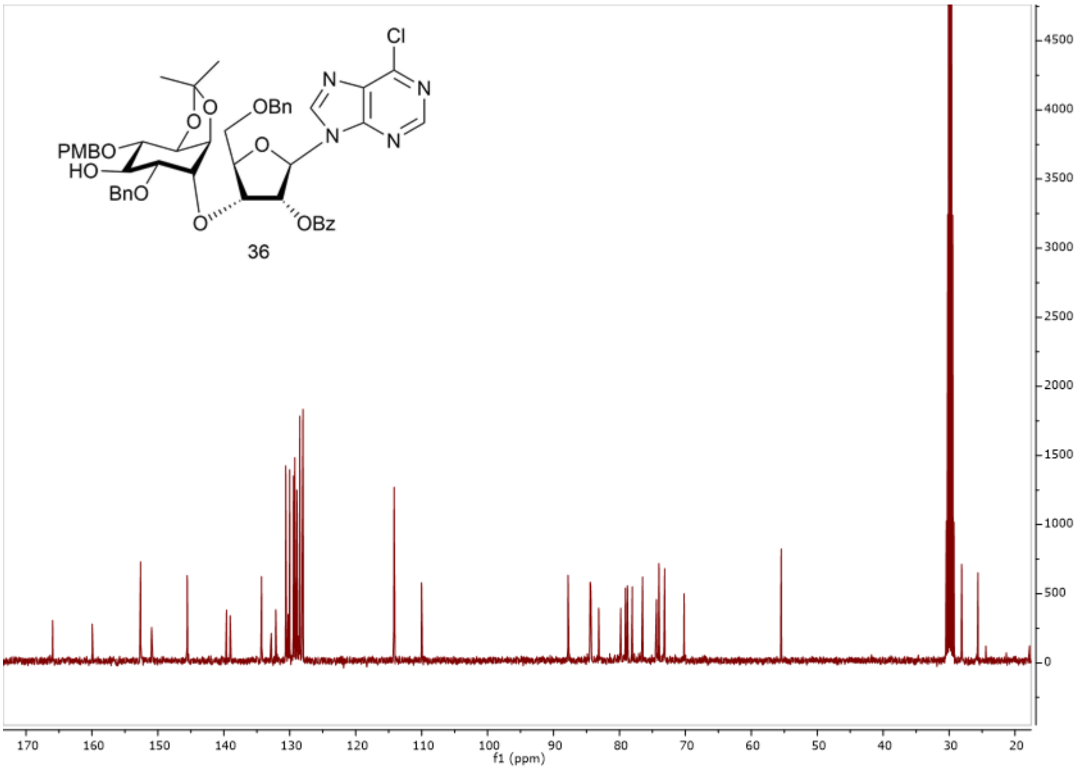

Compound 37.

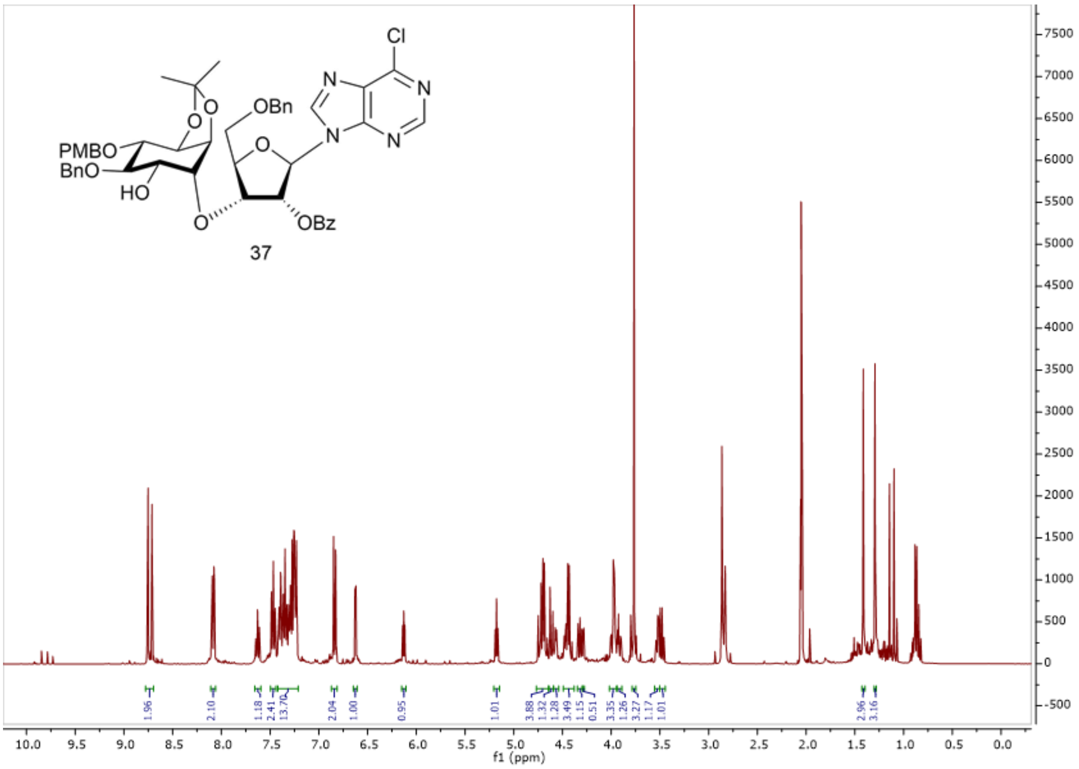

Cl
N
N
OBn
O
O
PMBO
BnO
HO
O
N
N
OBz
37
f1 (ppm)

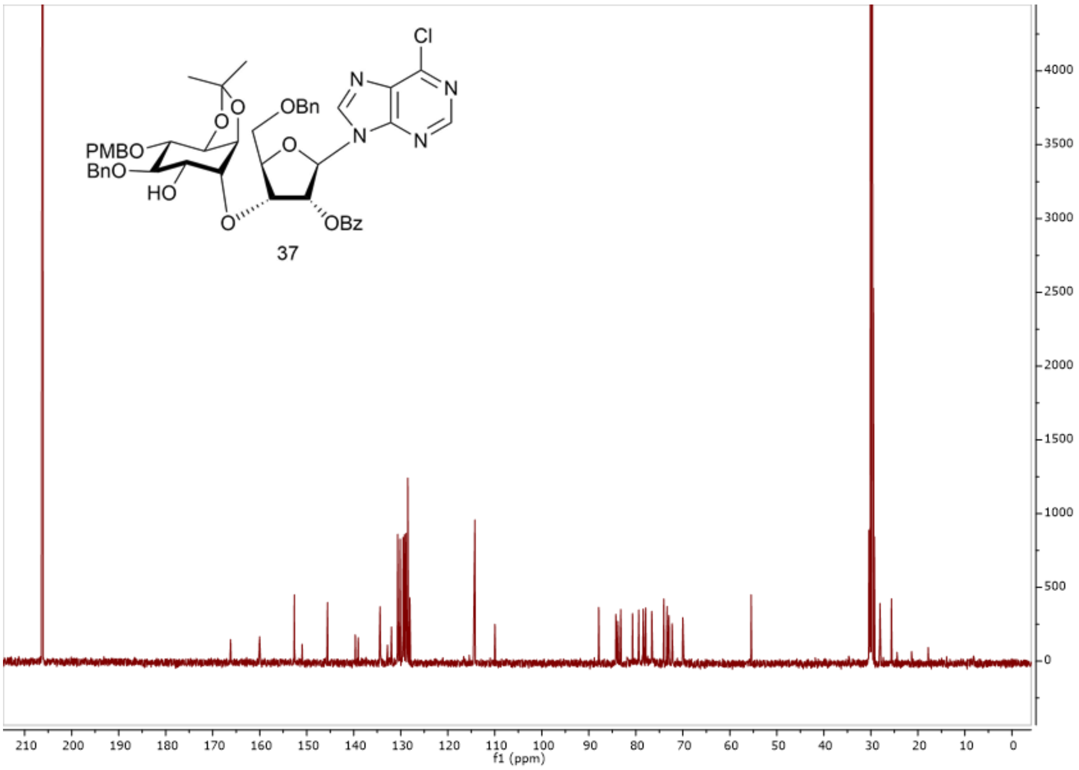

Cl
N
N
OBn
O
O
PMBO
BnO
HO
O
N
N
OBz
37
f1 (ppm)

Compound 12.

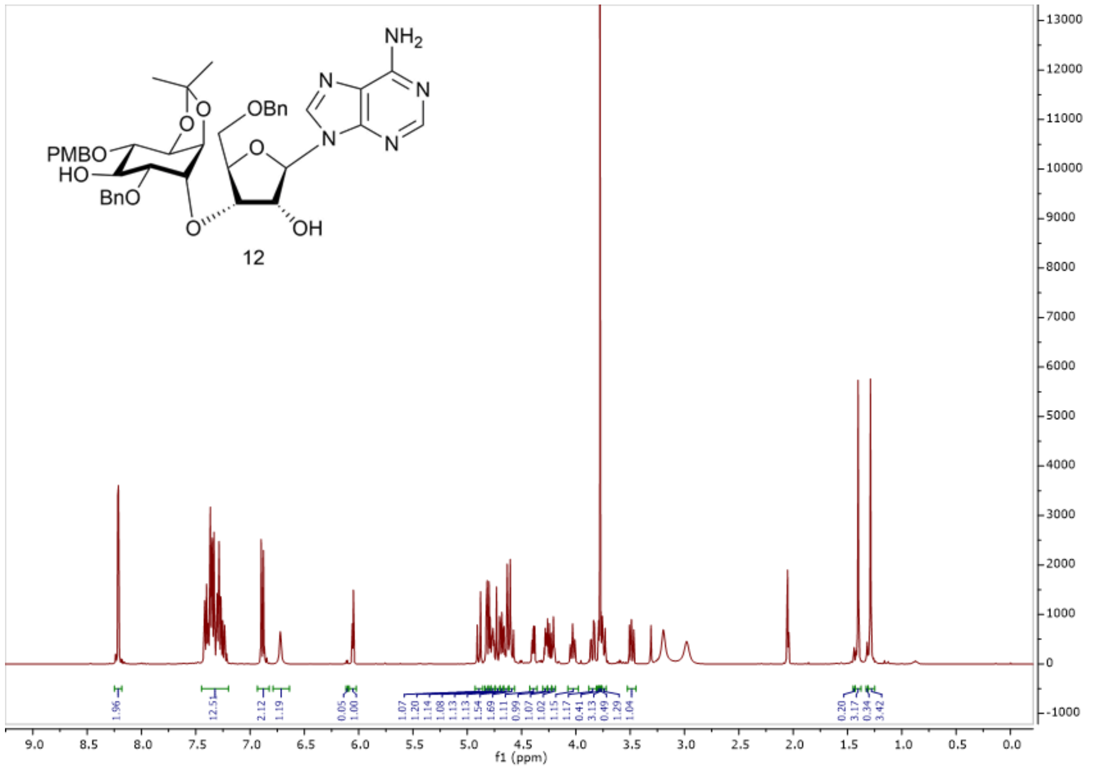

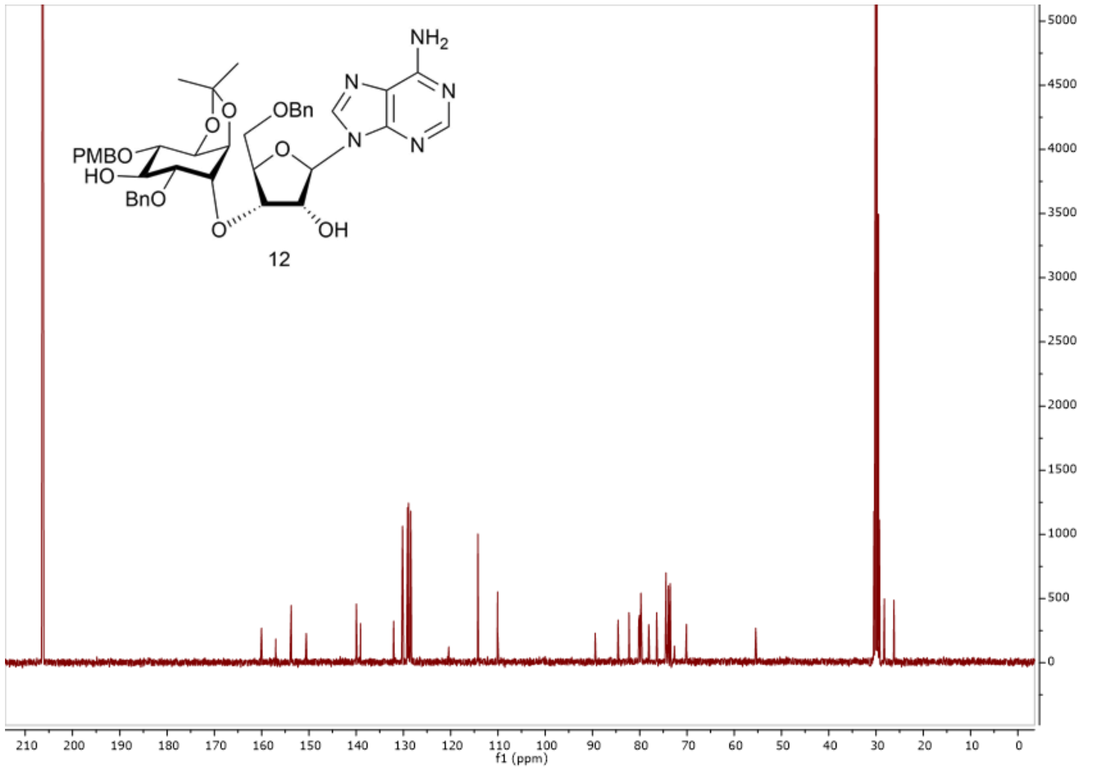

Compound 11.

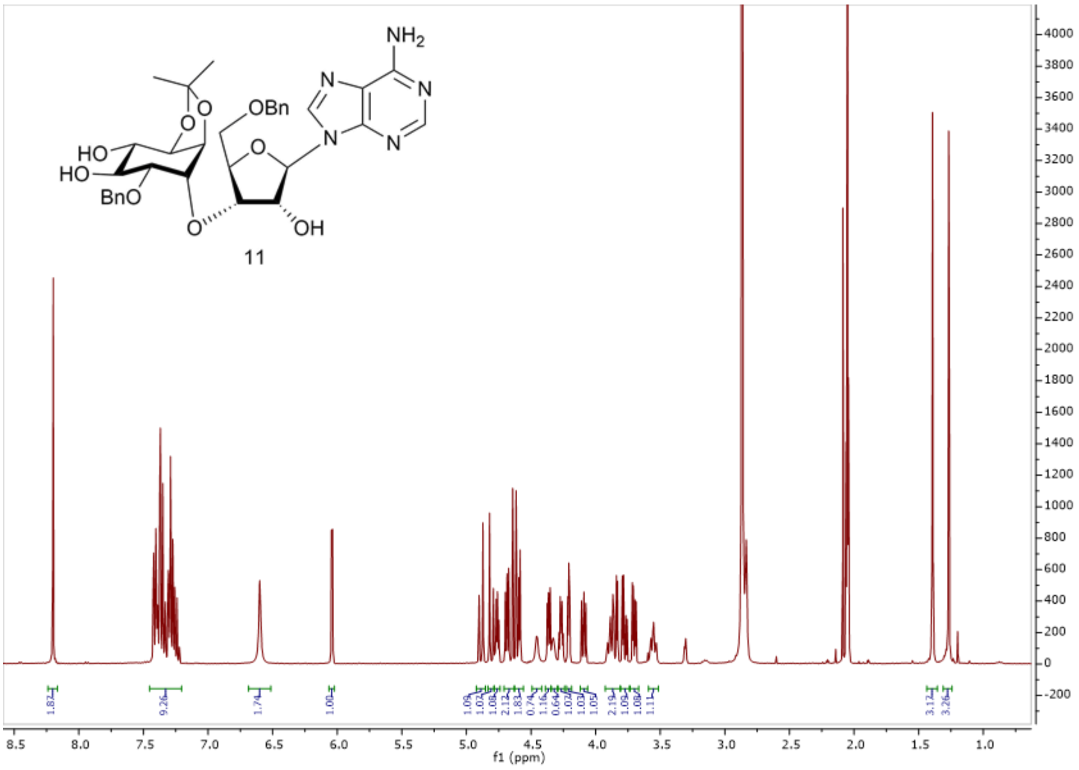

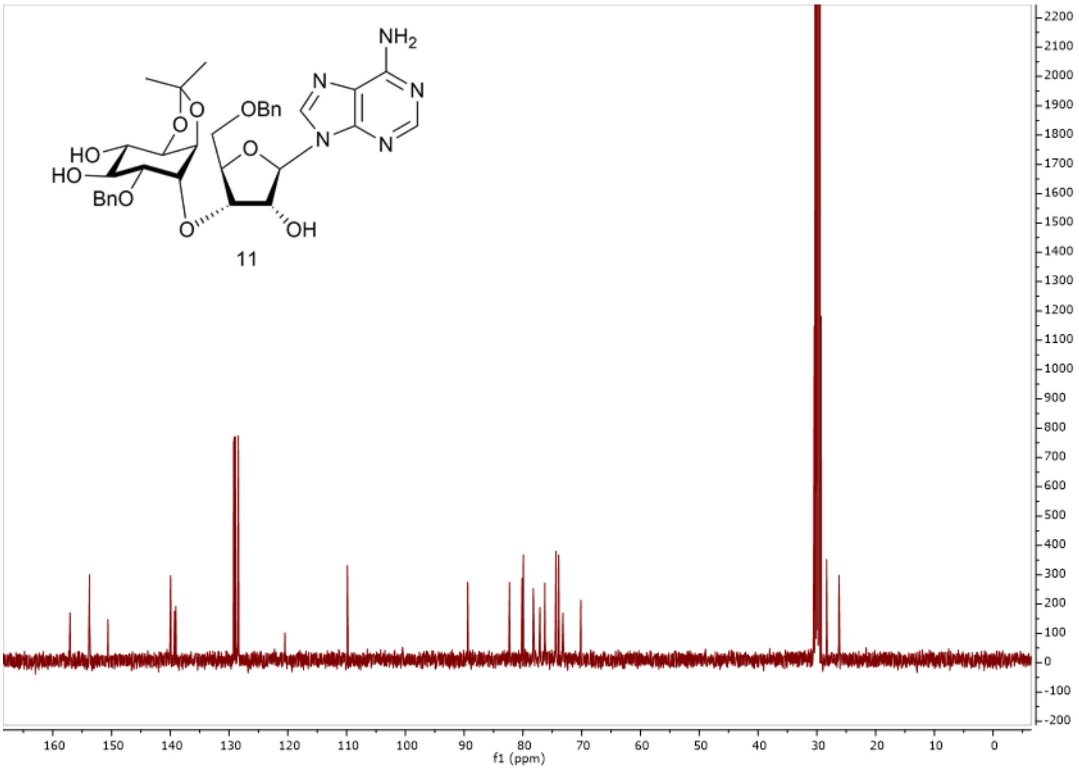

^1H and ^{31}P NMR spectra.

Compound 38.

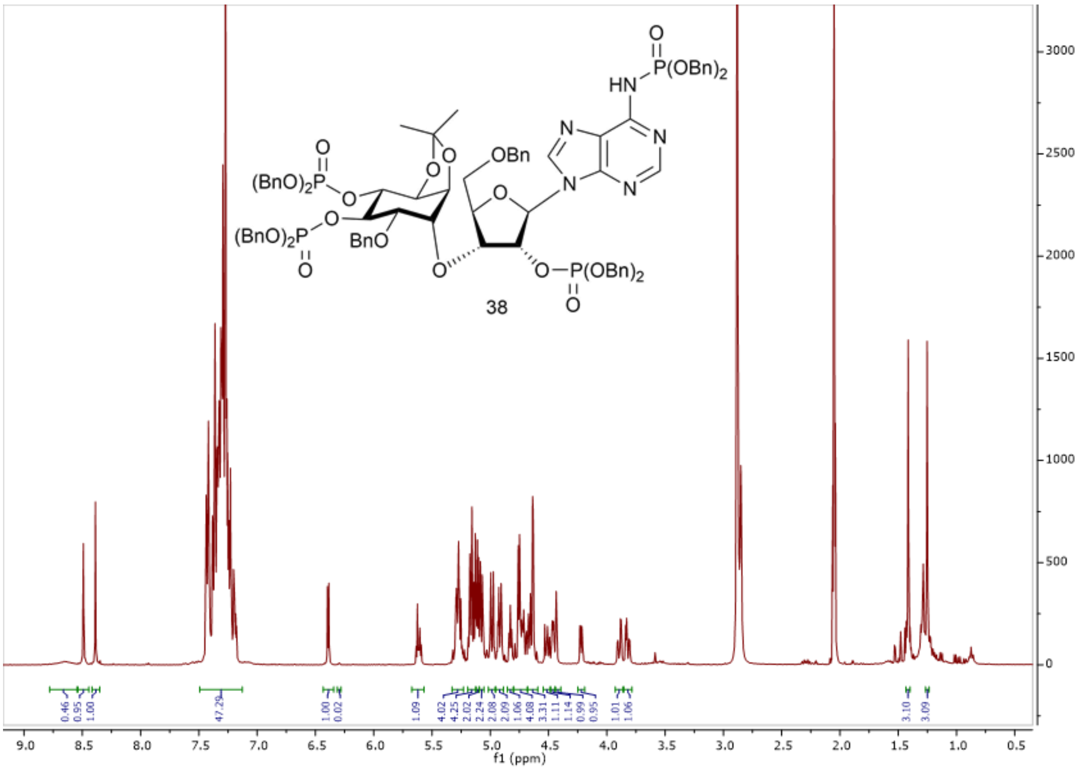

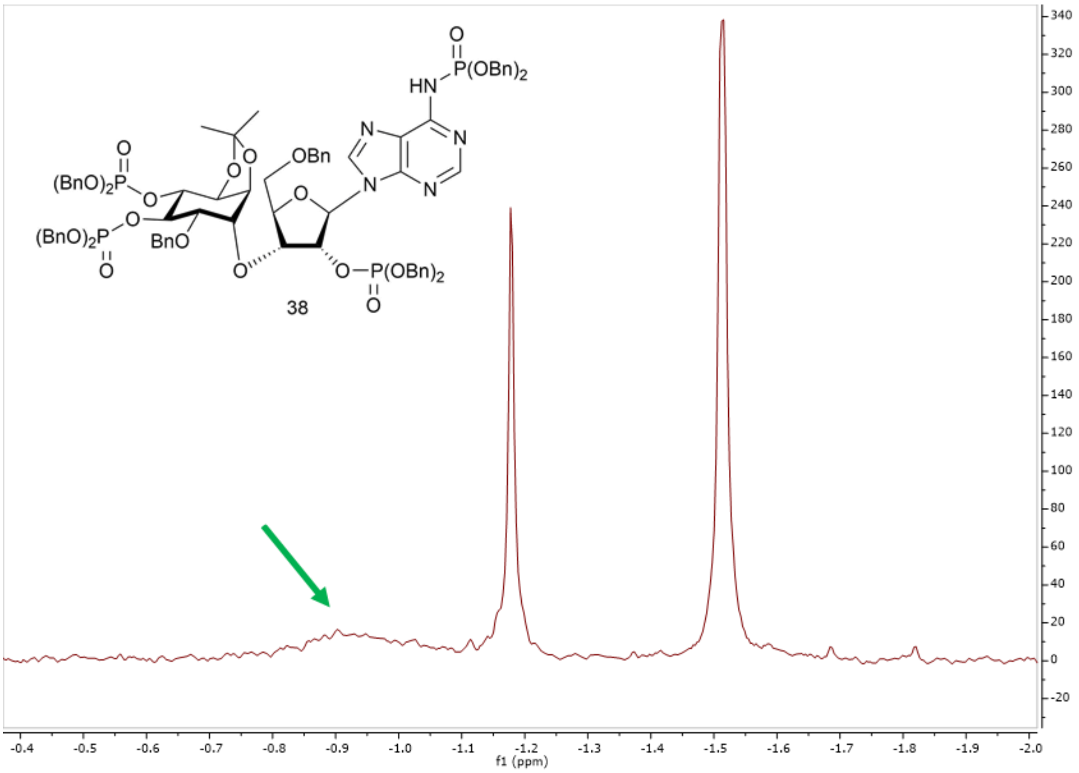

Compound 10.

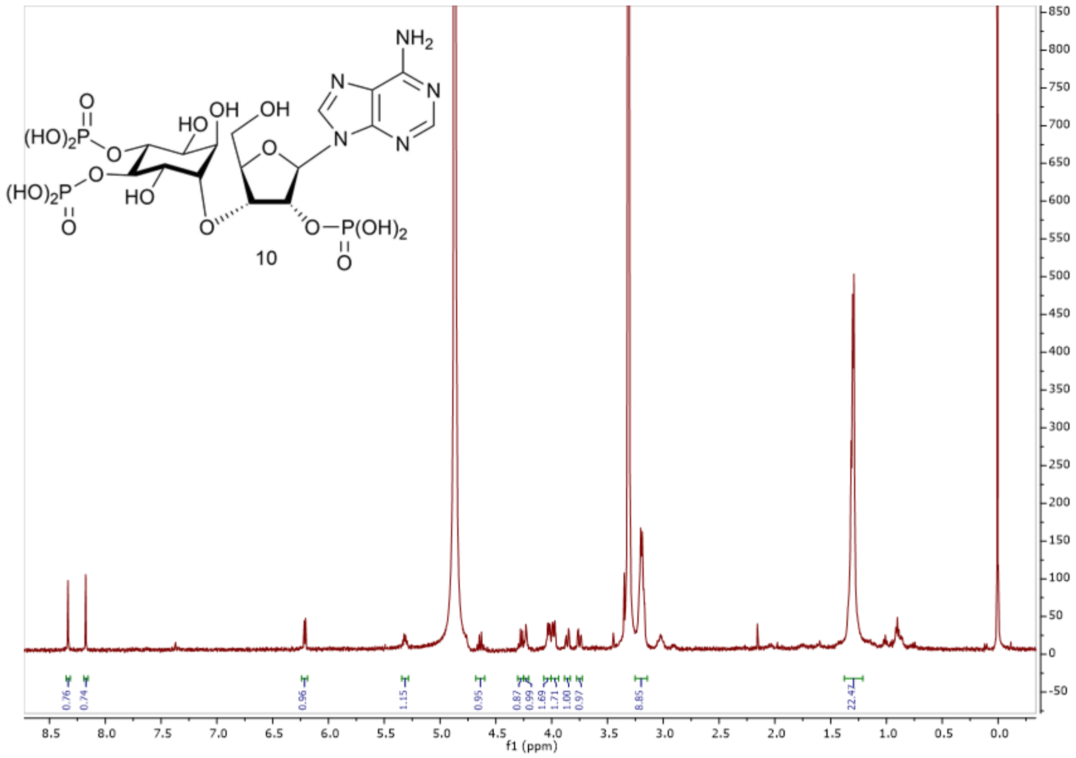

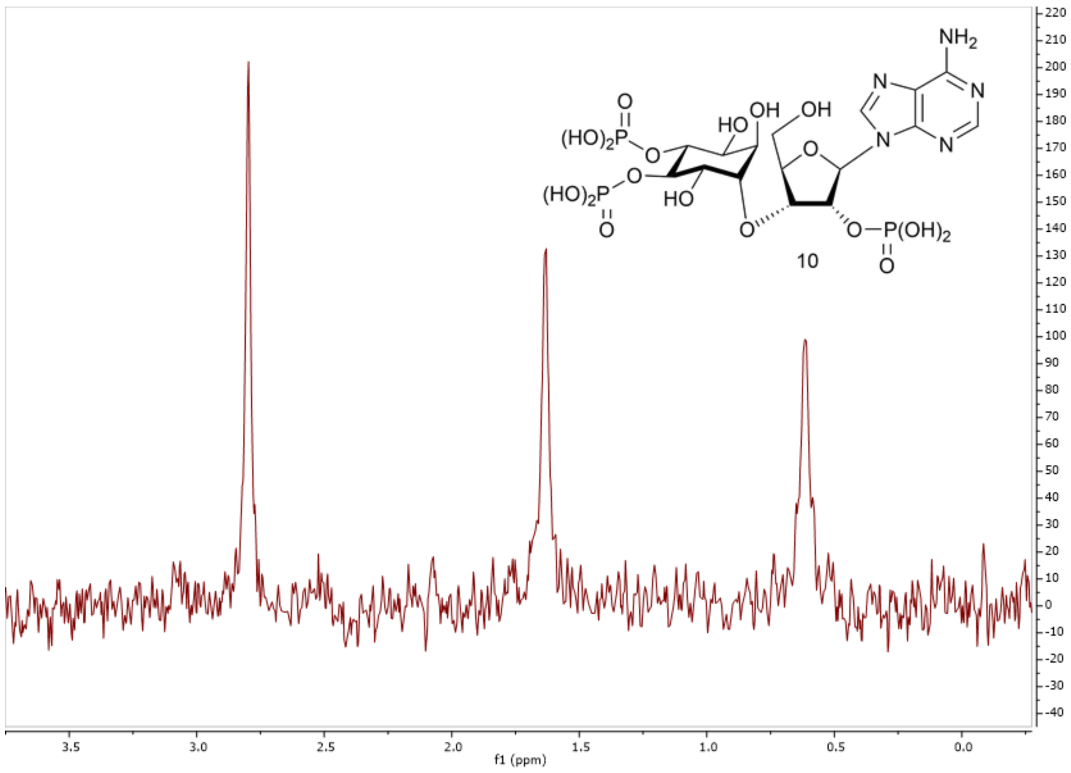

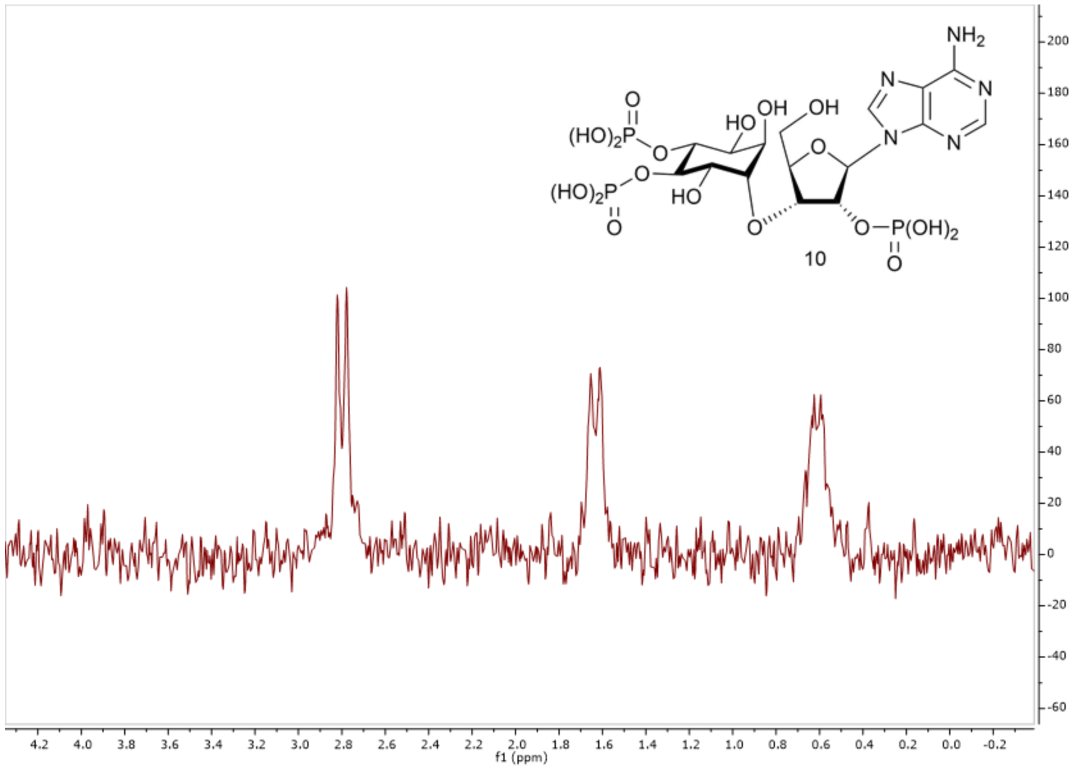

10
f1 (ppm)

^{1}H-^{31}P correlation and COSY NMR spectra of compound 10.

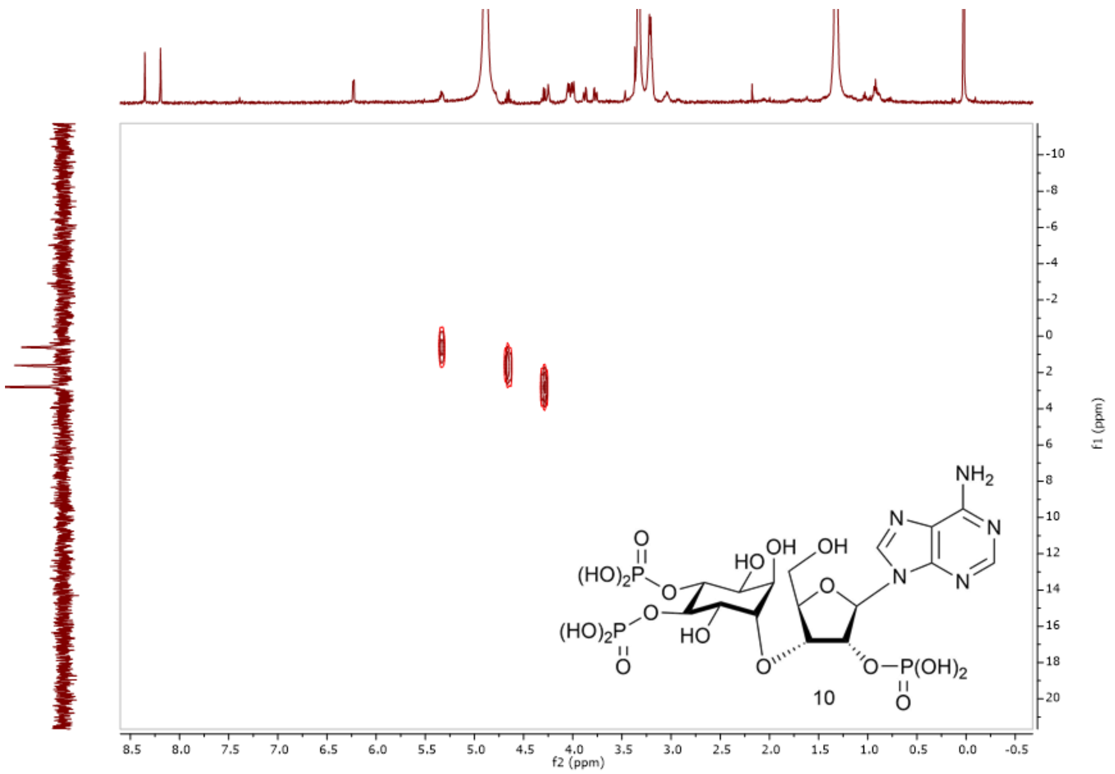

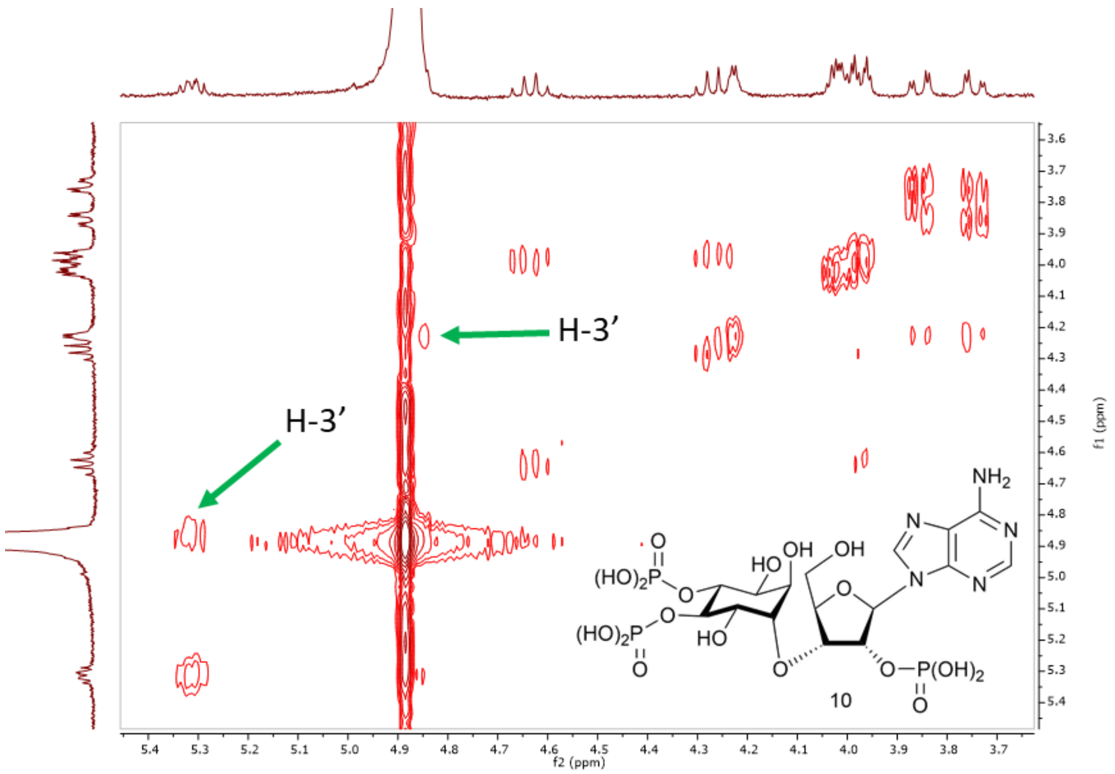

Biology

Materials. HEK-293 cells in which the genes for all three IP_3R subtypes had been disrupted using CRISPR-Cas9 technology (HEK-3KO)[12] were from Kerafast (Boston, USA). Dulbecco's modified Eagle's medium/nutrient mixture F-12 with GlutaMAX (DMEM/F-12 GlutaMAX) and Mag-fluo-4 AM were from ThermoFisher. TransIT-LT1 transfection reagent was from Geneflow (Elmhurst, Lichfield, UK). Most chemicals and foetal bovine serum (FBS) were from Sigma-Aldrich (Gillingham, UK). C*yclopiazonic acid* (CPA) was from Tocris (Bristol, UK). G418 was from Formedium (Norfolk, UK). Half-area 96-well black-walled plates were from Greiner. D-*myo*-Inositol 1,4,5- trisphosphate (IP_3) was from Enzo (Exeter, UK).

Cell Culture and Transfection. HEK cells were cultured in DMEM/F-12 GlutaMAX medium supplemented with 10% FBS at 37°C in 95% air and 5% CO_2. Cells were passaged or used for experiments when they reached confluence. HEK cells expressing only IP_3R1 (HEK-IP_3R1) were generated by transfecting HEK-3KO cells with the gene encoding rat IP_3R1 (lacking the S1 splice site)[13] cloned into pcDNA3.1(-)/Myc-His B plasmid[14] using TransIT-LT1 reagent following the manufacturer's instructions. To generate stable cell lines, cells were passaged 48 h after transfection in medium with G418 (1 mg/mL). Selection was maintained for 2 weeks, and medium was changed every 3 days. Monoclonal cell lines were selected by plating cells (~1 cell/well) into 96-well plates in medium containing G418 (1 mg/mL). After 4 days, wells with only one cell were identified and the cells were allowed to reach confluence. These cell lines were then expanded and their expression of IP_3R1 was confirmed by western blot using an antibody specific for IP_3R1.[13]

Ca^{2+} Release Assays. To monitor the free [Ca^{2+}] within the lumen of the endoplasmic reticulum (ER), the low-affinity Ca^{2+} indicator Mag-fluo-4 was used.[15] The ER of HEK-IP_3R1 cells was loaded with the Ca^{2+} indicator by incubating cells (2.4×10^7 cells/mL, 1 h, 22 °C) in HEPES-buffered saline (HBS; 135 mM NaCl, 5.9 mM KCl, 11.6 mM HEPES, 1.5 mM $CaCl_2$, 11.5 mM glucose, 1.2 mM $MgCl_2$, pH 7.3) supplemented with BSA (1 mg/mL), Pluronic F127 (0.4

mg/mL) and Mag-fluo-4 AM (20 μM). Cells were then suspended in Ca^{2+}-free cytosol-like medium (CLM: 20 mM NaCl, 140 mM KCl, 1 mM EGTA, 20 mM Pipes, 2 mM $MgCl_2$, pH 7.0) and permeabilized with saponin (10 µg/mL, 3 min, 37 °C). Permeabilized cells were centrifuged (650 x*g*, 3 min), and incubated in CLM (10 min, 37 °C) to allow the Ca^{2+} stores to empty. Cells were then centrifuged (650 x*g*, 3 min) and re-suspended in CLM without Mg^{2+}, but supplemented with 375 μM $CaCl_2$ to give a final free [Ca^{2+}] of 220 nM after addition of 1.5 mM MgATP. Cells (~4 x 10^5/well) were added to poly-L-lysine-coated half-area 96-well black-walled plates. Fluorescence was recorded at 20 °C at intervals of 1.44 s using a FlexStation III plate-reader (Molecular Devices, Sunnyvale, CA, USA) with excitation and emission wavelengths of 485 nm and 520 nm, respectively. MgATP (1.5 mM) was added to initiate Ca^{2+} uptake, and when the ER had loaded to steady-state (~2.5 min), *cyclopiazonic acid* (*CPA, 10* µM) was added to inhibit the ER Ca^{2+} pump. IP_3 or other ligands were added after a further 60 s. The amount of Ca^{2+} released was calculated as a percentage of the fluorescence signal from fully loaded stores (F_{full}) minus the signal from non-loaded stores ($F_{full} - F_{empty}$).

Data Analysis. Concentration-effect relationships were fitted to Hill equations (GraphPad Prism, version 5) from which pEC_{50} values (-log of the half-maximally effective ligand concentration) were obtained. The pEC_{50} values are presented as means ± SEM. Statistical analysis used repeated measures ANOVA followed by Bonferroni's Multiple Comparison Test (GraphPad Prism, version 5). $^*P < 0.05$ was considered significant.

References

1. D. B. G. Williams, and M. Lawton, *J. Org. Chem.*, 2010, **75**, 8351.
2. J. Gigg, R. Gigg, S. Payne and R. Conant, *Carbohydr. Res.*, 1985, **142**, 132.
3. S.-K. Chung and Y. Ryu, *Carbohydr. Res.*, 1994, **258**, 145.
4. G. Reyes-Rangel, J. Vargas-Caporali and E. Juaristi, *Tetrahedron*, 2016, **72**, 379.
5. H. Gerlach, *Helv. Chim. Acta*, 1985, **68**, 1815.
6. L. D. Nord, N. K. Dalley, P. A. McKernan and R. K. Robins, *J. Med. Chem.*, 1987, **30**, 1044.
7. C. V. S. Ramamurty, P. Ganney, C. S. Rao and B. Fraser-Reid, *J. Org. Chem.*, 2011, **76**, 2245.
8. B. Fraser-Reid, P. Ganney, C. V. S. Ramamurty, A. M. Gómez and J. C. López, *Chem Commun.*, 2013, **49**, 3251.
9. V. Salazar-Pereda, F. J. Martinez-Martinez, R. Contreras and A. Flores-Parra, *J. Carbohydr. Chem.*, 1997, **16**, 1479.
10. J. Gigg, R. Gigg, S. Payne and R. Conant, *J. Chem. Soc. Perkin Trans. I*, 1987, 1757.
11. A. M. Riley, H. Wang, S. B. Shears and B. V. L. Potter, *Chem. Commun.*, 2015, **51**, 12605.
12. K. J. Alzayady, L. Wang, R. Chandrasekhar, L. E. Wagner II, F. Van Petegem and D. I. Yule, *Sci. Signal.*, 2016, **9**, ra35.
13. A. M. Rossi, A. M. Riley, S. C. Tovey, T. Rahman, O. Dellis, E. J. A. Taylor, V. G. Veresov, B. V. L. Potter and C. W. Taylor, *Nat. Chem. Biol.*, 2009, **5**, 631.
14. F. Guo, M. Y. Chiang, Y. Wang and Y. Z. Zhang, *Biotechnol. J.*, 2008, **3**, 370.
15. S. C. Tovey, Y. Sun and C. W. Taylor, *Nat. Prot.*, 2006, **1**, 259.
